# Genomic and Epidemiological Analysis of SARS-CoV-2 Viruses in Sri Lanka

**DOI:** 10.3389/fmicb.2021.722838

**Published:** 2021-09-16

**Authors:** Chandima Jeewandara, Deshni Jayathilaka, Diyanath Ranasinghe, Nienyun Sharon Hsu, Dinuka Ariyaratne, Tibutius Thanesh Jayadas, Deshan Madhusanka Panambara Arachchige, Benjamin B. Lindsey, Laksiri Gomes, Matthew D. Parker, Ananda Wijewickrama, Malika Karunaratne, Graham S. Ogg, Thushan I. de Silva, Gathsaurie Neelika Malavige

**Affiliations:** ^1^Allergy, Immunology and Cell Biology Unit, Department of Immunology and Molecular Medicine, University of Sri Jayewardenepura, Nugegoda, Sri Lanka; ^2^Department of Infection, Immunity and Cardiovascular Diseases, Medical School, University of Sheffield, Sheffield, United Kingdom; ^3^Sheffield Bioinformatics Core, The University of Sheffield, Sheffield, United Kingdom; ^4^National Institute of Infectious Diseases, Angoda, Sri Lanka; ^5^MRC Weatherall Institute of Molecular Medicine, University of Oxford, Oxford, United Kingdom

**Keywords:** SARS-CoV-2, genomic sequencing, pangolin lineages, B.1.411, B.1.1.7, molecular epidemiology

## Abstract

**Background:** In order to understand the molecular epidemiology of severe acute respiratory syndrome coronavirus-2 (SARS-CoV-2) in Sri Lanka, since March 2020, we carried out genomic sequencing overlaid on available epidemiological data until April 2021.

**Methods:** Whole genome sequencing was carried out on diagnostic sputum or nasopharyngeal swabs from 373 patients with COVID-19. Molecular clock phylogenetic analysis was undertaken to further explore dominant lineages.

**Results:** The B.1.411 lineage was most prevalent, which was established in Sri Lanka and caused outbreaks throughout the country until March 2021. The estimated time of the most recent common ancestor (tMRCA) of this lineage was June 1, 2020 (with 95% lower and upper bounds March 30 to July 27) suggesting cryptic transmission may have occurred, prior to a large epidemic starting in October 2020. Returning travellers were identified with infections caused by lineage B.1.258, as well as the more transmissible B.1.1.7 lineage, which has replaced B.1.411 to fuel the ongoing large outbreak in the country.

**Conclusions:** The large outbreak that started in early October, is due to spread of a single virus lineage, B.1.411 until the end of March 2021, when B.1.1.7 emerged and became the dominant lineage.

## Introduction

The severe acute respiratory syndrome coronavirus-2 (SARS-CoV-2) has emerged as the leading cause of mortality in several countries in the world. As of May 24, 2021, 167 million cases and 3.45 million deaths have been reported worldwide (John Hopkins University, 2021). Due to the emergence of variants of concern, the World Health Organization has recommended whole genomic sequencing of the SARS-CoV-2 viruses within countries regularly and systematically for early identification of such variants (World Health Organization, 2021).

The first patient infected with SARS-CoV-2 in Sri Lanka was reported on the January 27, 2020, who was a foreign national, with the first Sri Lankan patient reported on the of March 10, 2020 (Ministry of Health, 2020a). In the following 6 months (March–September), the spread of the virus was largely contained with only 3,111 reported cases, of which 38.8% were imported (Ministry of Health, 2020b). However, there was a surge in the number of cases with discovery of a new cluster in early October 2020 in a clothing factory in the district adjacent to Colombo (Gampaha). This was followed by rapid spread of SARS-CoV-2 within the Colombo Municipality region (CMC), fish markets and subsequently to the whole country. This outbreak continued to evolve, with infections being reported in all regions of the country, but the numbers gradually declined by end of March 2021, with the number of daily cases been reported falling to 200–300 cases per day (Ministry of Health, 2021a). The number of daily cases remained <250 cases/day until mild-April 2021, when a gradual and steep rise in the number of cases was seen with daily infections rising over 1,000/day by the last week of April to over 2,500/day by mid-May (Ministry of Health, 2021b).

We carried out SARS-CoV-2 sequencing from isolates collected throughout the different phases of the pandemic in order to determine the molecular epidemiology of SARS-CoV-2 in Sri Lanka, including current circulation of viruses with mutations that may confer greater transmissibility and/or threaten the efficacy of vaccines.

## Materials and Methods

### Patients

Initial real-time quantitative PCR (RT-PCR) of diagnostic sputum or nasopharyngeal swabs were carried out using TaqPath COVID-19 CE-IVD RT-PCR kit (Thermo Fisher Scientific, United States) according to the manufacturer’s instructions. 373 positive samples with cycle threshold (Ct) values <30 was directed to whole genome sequencing. Ethical approval for the study was obtained by the Ethics Review Committee of the University of Sri Jayewardenepura.

### Viral RNA Extraction

Viral RNA was extracted using QIAamp viral RNA mini kit (Qiagen, United States), SpinStarTM Viral Nucleic Acid Extraction kit 1.0 (ADT Biotech, Malaysia) or FastGene RNA Viral Kit (Nippon Genetics, Germany), according to manufacturer’s instructions. Presence of ORF1ab gene and N gene of SARS-CoV-2 was detected with Novel Coronavirus (2019-Ncov) Nucleic Acid Diagnostic Kit (Sansure Biotech) and S gene was detected by Taqpath COVID-19 RT PCR kit (Applied Biosystems) by real time RT PCR in ABI 7500 real time PCR system (Applied Biosystems, United States).

### Library Preparation and Next Generation Sequencing

Library preparation was attempted using either the TruSeq Stranded Total RNA Library Prep Gold (Illumina, San Diego, CA, United States) or The AmpliSeq for Illumina SARS-CoV-2 Community Panel, in combination with AmpliSeq for Illumina library prep, index, and accessories (Illumina, San Diego, CA, United States). Shotgun metagenomic sequencing workflow was used for four initial samples, while for the remainder (*n* = 369) a targeted RNA/cDNA amplicon assay was used (Supplementary Methods).

### Phylogenetic Analysis of Severe Acute Respiratory Syndrome Coronavirus-2 Sequences

Sequences (*n* = 373) with ≥10× median read depth and ≥86% genome coverage were taken forward for further analysis. Their GISAID accession numbers, location, and the clades of sequences used are shown in Supplementary Table 1. The 373 Sri Lanka sequences were combined with 3,200 representative global sequences to evaluate the phylogenetic relationships of the viruses circulating in Sri Lanka in relation to the global phylogenetic population. The representative sample of global sequences was obtained in two steps using all available data on GISAID up until May 12, 2021. The first step included randomly selecting one sequence per country per epidemiological week (week 0 refers to December 22–28, 2019, when the first Wuhan genome was collected). This was then followed by a random sampling of the remaining sequences to generate a sample of 3,200 sequences. All sequences were then aligned to the SARS-CoV-2 reference strain MN908947.3 using MAFFT version 7.477 (Katoh et al., 2002). We masked alignment positions that have been previously flagged as problematic^1^ and manually removed obvious sequencing errors and potential homoplasic positions. A maximum likelihood tree was constructed using IQ-TREE2 version 2.1.2 (Minh et al., 2020). Following Rambaut et al. (2020) the IQ-TREE2 analysis was performed using the GTR+G model of nucleotide substitution (Tavaré, 1986; Yang, 1994) and 1,000 replicates of ultrafast bootstrapping (−B 1000) and SH-aLRT branch test (−alrt 1000).

The time-scaled phylogeny was undertaken to estimate the emergence time of B.1.411 and the introduction time of B.1.1.7 in Sri Lanka. The time-scale phylogeny were reconstructed using both a Bayesian approach (BEAST) and a maximum likelihood-based method (Sagulenko et al., 2018). The analyses were performed using sequences from Sri Lanka only. The alignment and maximum likelihood tree construction were performed using MAFFT and IQ-TREE2 as described above. TempEst (v1.5.3) (Rambaut et al., 2016) was used to examine the temporal signal of the sequences, as well as to identify outlier sequences in the tree. 18 sequences were strong outlier and removed from the analysis, leaving a total of 355 sequences in the final dataset. Repeating the above steps, we confirmed that there was a strong temporal signal in the final dataset with *R*^2^ of 0.49 and correlation coefficient of 0.70 (using the correlation best-fitting root function). No further outliers were identified from the final dataset.

Using a BEAST implemented in BEAST v1.10.4 (Suchard et al., 2018), time-scaled phylogenetic reconstruction was performed to estimate the timing and the spread of SARS-CoV-2 viruses in Sri Lanka. The HKY model was used to model nucleotide evolution (Hasegawa et al., 1985) with a relaxed molecular clock. Following the previous estimation by Duchene et al. (2020), the initial evolutionary clock rate was set to 0.001 substitutions per site per year. An exponential growth coalescent tree prior was used to account for the characteristics of early stages of SARS-CoV-2 outbreak (Fountain-Jones et al., 2020; Volz et al., 2021a). The BEAST analysis was ran with the MCMC chain length of 40 million and sampling frequency of 2000. Convergence was assessed using Tracer (Rambaut et al., 2018). All model parameters had effective sample size (ESS) values >200, confirming sufficient mixing and convergence to stationary. The maximum clade credibility tree was generated using TreeAnnotator (v1.10.4), with 20% removed as burn-in. The molecular clock phylogeny was also inferred using a maximum likelihood-based method TreeTime (Sagulenko et al., 2018), shown in Supplementary Figure 2. Visualisation of the phylogenetic trees were produced in R (v4.0.1), using ggtree package. Lineages were assigned using Pangolin (version v2.4.2, lineages version 2021-04-28). Code to reproduce the BEAST analysis is provided in Supplementary File 2. R code to reproduce the tree visualisation is provided in Supplementary File 3.

Two proportional symbol maps of Sri Lanka were plotted with GPS coordinates of the sampling locations of B.1.411 and B.1.17 sequences using R (v4.0.1). Each sampling location was indicated by a coloured bubble proportionate to the number of sequences sampled within. Colombo district was zoomed into a sub map (longitude: 79.80–79.98, latitude: 6.80–6.98) in order to visualize the suburbs as Colombo had the highest sampling density. R code to reproduce the map visualisation is provided in Supplementary File 4.

## Results

**FIGURE 1:**
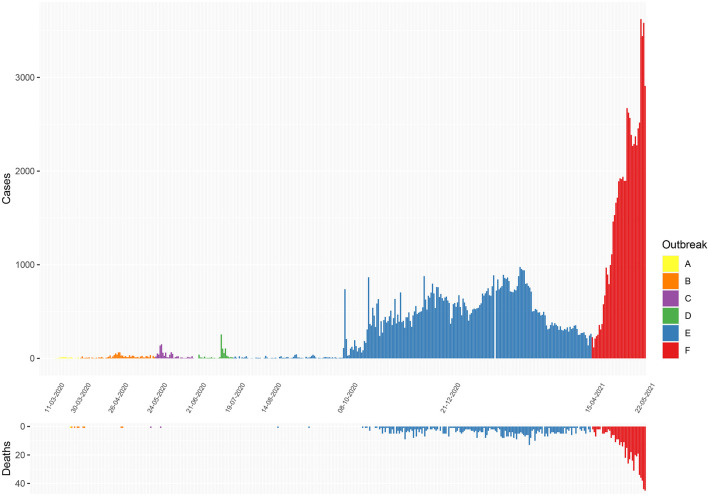
Epidemiological curves of COVID-19 cases and deaths reported in Sri Lanka from March 11, 2020 to May 22, 2021. (Data from Epidemiology Unit, Ministry of Health, Sri Lanka) (Ministry of Health, 2020a). A, initial outbreak from overseas returned; B, closed outbreak in a the Colombo Municipality area; C, returnees from Middle East; D, outbreak at the drug rehabilitation centre; E, the large outbreak that initially began in a clothing factory; and F, the current ongoing outbreak.

**FIGURE 2:**
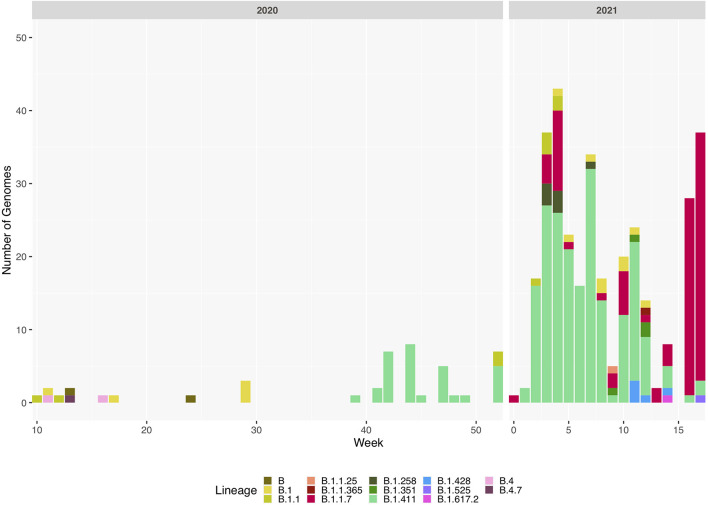
Sri Lanka sequence lineage distribution since the detection of SARS-CoV-2 in Sri Lanka. Lineage breakdown of 373 Sri Lanka samples across time. B.1.411 was first detected in week 39 in 2020 and since B.1.411 has dominated the outbreak in Sri Lanka until week 12, 2021. B.1.1.7 was detected at the beginning of 2021 and its prevalence has rapidly increased in Sri Lanka.

Of six samples collected in March 2020 from returning travellers and their contacts (period A, Figure 1: four from Colombo district, two from Kalutara district), two belonged to lineage B.4, two to B 1.1, one to B.1 and one to B (Figure 2 and Supplementary Table 1). During early April, SARS-CoV-2 spread within closed community clusters in the CMC region (period B, Figure 1). Two viruses from these clusters belonged to lineages B.4 and B.1 (Supplementary Table 1). Period C in Figure 1 was thought to be due to an outbreak initiated by the returning workforce from the Middle East. A sequence was obtained from only one virus, which belonged to lineage B. Again, due to detection of infected patients at the airport and mandatory quarantine of all individuals for at least 14 days, during May to June, cases appeared not to spill over to the community. However, there was a sudden surge in the number of cases in mid-July in a drug rehabilitation centre (DRC), in the North Central Province (period D, Figure 1). The origin of this outbreak was not known and three lineage B.1 sequences obtained, formed a cluster separate from earlier B.1 sequences from Sri Lanka. This outbreak was also subsequently controlled.

Sri Lanka then had a period of approximately 2 months during August and September, where no locally acquired infections were reported (Ministry of Health, 2020a). In early October, an outbreak occurred in a clothing factory that heralded a wave of infections, which continued to the end of March 2021. This outbreak in early October was soon followed by widespread transmission in many factories, fish markets island wide, in the highly populated CMC region in Colombo and subsequently to many areas in the country (period E, Figure 1). Following a period of intense spread in the CMC for 4 months, the number of cases gradually declined from January 2021 to early April 2021. However, a rapid rise in the number of cases were seen from mid-April 2021 (period F, Figure 1), with the numbers exponentially increasing as of May 24, 2021.

### Outbreak With the B.1.411 Lineage

**FIGURE 3:**
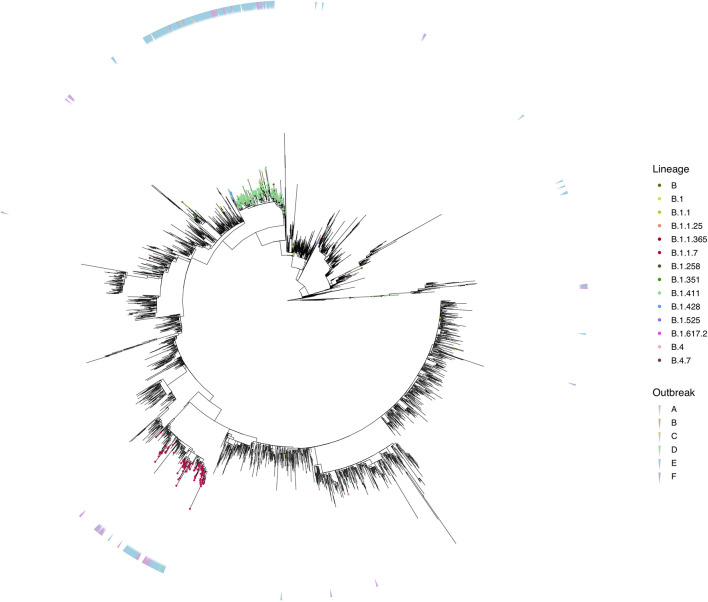
Phylogenetic analysis of Sri Lankan sequences. Maximum likelihood tree of 373 Sri Lanka samples and 3,200 representative global sequences obtained from GISAID. Tips are coloured by Pango lineages, and the external layer on the right indicates the outbreak period associated with each Sri Lanka sample. The tree was estimated using IQTree2 (GTR maximum likelihood model and +G heterogeneity rate) and 1,000 replicates of ultrafast bootstrapping (−B 1000) and SH-aLRT branch test (−alrt 1000).

**FIGURE 4:**
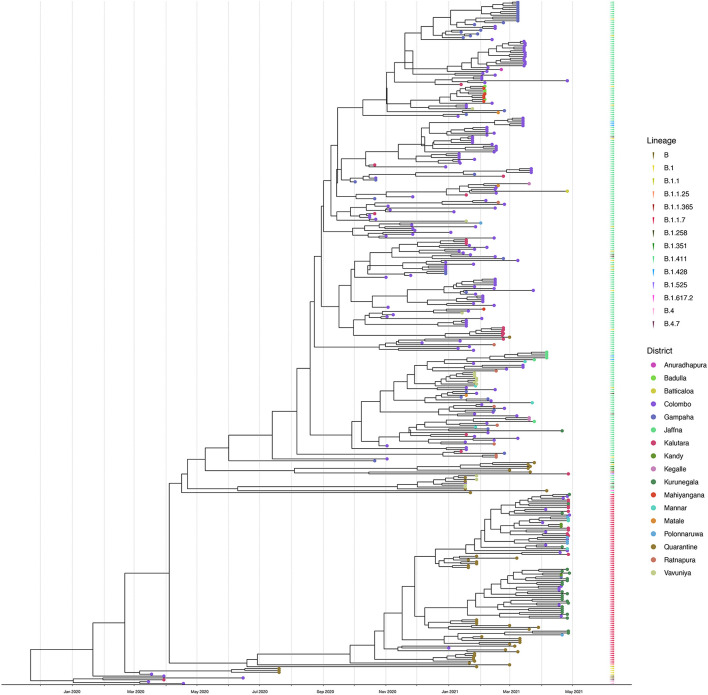
Bayesian time-scale phylogeny of Sri Lankan samples. Tips are coloured by the sample location, and the external layer on the right shows the sample lineages. The molecular clock was inferred using BEAST with HKY evolution model and a relaxed molecular clock (initial evolutionary rate was set to 0.001 substitutions/site/year). See Supplementary Figure 1 for the Bayesian tree with all posterior support values.

**FIGURE 5:**
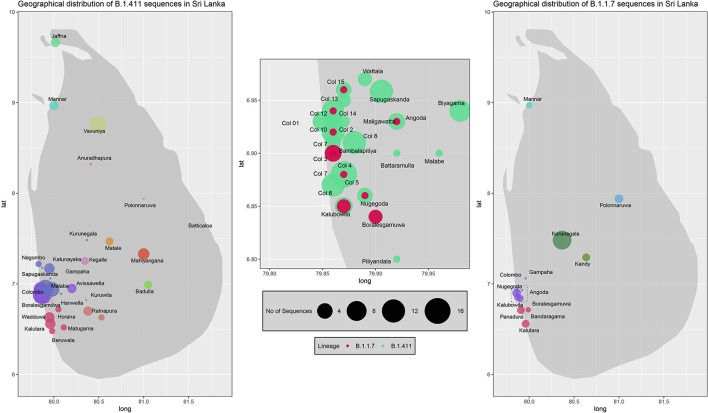
Geographical distribution of SARS-CoV-2 B.1.411 and B.1.17 lineage infections in Sri Lanka sampled from October 2020 to April 2021. Radius of each bubble accounts for the number of sequences reported from its representing district and the colour code based on the location is adopted from the Figure 4. Colombo district is expanded in the middle to visualize its suburbs.

We sequenced 361 samples taken during October 2020 to April 30, 2021, to determine how the outbreak evolved and establish if there were any new introductions to fuel ongoing infections. 231 of these 361 viruses were classified into a novel lineage B.1.411. The global phylogenetic tree shown in Figure 3 suggests that the B.1.411 viruses are distinct from other global sequences and was predominantly seen in Sri Lanka. This lineage was responsible for the period E outbreak (October 2020 to early April 2021) in Sri Lanka (Figure 2). A Bayesian time-scale phylogeny produced using BEAST (Figure 4) suggests that the estimated time of the most recent common ancestor (tMRCA) of the B.1.411 lineage is around June 1, 2020 (with 95% lower and upper bounds March 30 to July 27). A Maximum-likelihood method, TreeTime, also estimated a similar tMRCA of June 29, 2020 (with 95% lower and upper bounds May 23–July 30), shown in Supplementary Figure 2. The distribution of B.1.411 cases throughout the country is shown in Figure 5.

The predominant mutations in the spike protein were D614G seen in 228/231 sequences and H1159Y mutation seen in 215/231 sequences. Importantly, one B.1.411 genome had the E484K mutation while four sequences from had acquired N439K in the spike protein along with the other B.1.411 lineage defining mutations. Also, we observed that the ΔH69/V70 deletion in the spike had co-occurred in three of those with the N439K mutation. The other mutations seen in B.1.411 lineage are, T116I in NSP2, L37F in NSP6, P323L, and M666I in NSP12 and T205I in the N protein (Supplementary Table 2).

### Outbreak With the B.1.1.7 Lineage

Twenty seven infections sampled between January and late March 2021 belonged to the B.1.1.7 lineage. The first case of these was an imported infection on the January 02, 2021, with a further 26 imported cases detected between January and March 2021. Except one case infection that was seen in the community and another infection seen in a fisherman in March 2021, all other cases were identified within quarantine facilities during this period (Figure 2). After a gradual decline in the number of cases by the end of March, a rapid surge in the number of cases were seen since mid-April 2021. From the 78 samples sequenced from March 26 to April 30, 66 were found to be of the B.1.1.7 variant (Figures 2, 3). By the end of April, B.1.1.7 was the predominant variant circulating in many parts of the country (Figure 5).

### Other Severe Acute Respiratory Syndrome Coronavirus-2 Variants Detected

Twenty infections sampled from the community and quarantine centres were classified into United Kingdom dominant B.1.258 (*n* = 7), Danish lineage B.1.428 (*n* = 5) and B.1.351 lineage, first described in South Africa (*n* = 4). The rest of the sequences identified were B.1.1.25 (*n* = 1), B.1.1.365 (*n* = 1), B.1.525 (*n* = 1), and B.1.617.2 (*n* = 1). Both variants of concern (B.1.617.2 and B.1.351) were reported from returnees from India and Middle East.

## Discussion

We report the first description of SARS-CoV-2 molecular epidemiology in Sri Lanka from March 2020 to April 30, 2021. The virus strains identified in March 2020 belonged to clades B.1, B.2, B 1.1, and B.4, demonstrating that SARS-CoV-2 strains were introduced to Sri Lanka from multiple locations (Rambaut et al., 2020). Sri Lanka underwent a national lockdown very early in the pandemic on the March 20, 2020, when only 66 patients with SARS-CoV-2 were confirmed. This lockdown, which continued until mid-May, managed to contain the outbreak and prevent community transmission, except within isolated community clusters. A further contained outbreak occurred in mid-July within a DRC. Sequencing of a limited number of these samples showed that this outbreak was due to viruses belonging to lineage B.1 but which were distinct to the former B.1 samples. The outbreak in the DRC was also subsequently controlled and Sri Lanka did not report any cases of locally acquired infection during the months of August and September. Small numbers of reported cases were from imported infections only.

A large outbreak was discovered in early October after a clothing factory employee presented with pneumonia caused by SARS-CoV-2, which was followed by the emergence of a large second wave. We report that these were due to a lineage first detected in Sri Lanka, B.1.411, that dispersed throughout the country. The molecular clock analysis revealed that this lineage most likely emerged in mid-late June 2020 and therefore, it is possible that the virus was circulating in the community for several months before leading to the large outbreak that started in October. This highlights the potential for cryptic community transmission leading to a national epidemic wave even in the face of strict quarantine rules for returning travellers.

The B.1.411 Sri Lankan lineage has a unique spike mutation H1159Y in the C terminal region, which was seen in 215/231 viruses belonging to this lineage. The significance of this mutation is unknown. Also, the P323L mutation in NSP12 region, which is known to have co-evolved with D614G mutation was seen in 201/231 of the B.1.411 genomes (Kannan et al., 2020). Even though there is no direct correlation between P323L mutation and infectivity, given the fact that this mutation is widespread and almost 100% co-existent with D614G, some argue that this mutation could contribute to the enhanced viral replication and infectivity seen in D614G dominant strains (Kannan et al., 2020).

Most importantly, number of B.1.411 genomes carrying the E484K (*n* = 1) and N439K (*n* = 4) mutations in the spike protein were detected from both community and quarantine centres between January to mid-February 2021, demonstrating the potential for this lineage to evolve mutations that may evade antibody responses. Even though E484K mutation is predominantly seen in B.1.351 and P.1 lineages, recent evidence indicates introduction of this mutation into other lineages such as B.1.1.7 and B.1.243 (Wise, 2021). N439K mutation in the receptor binding motif (RBM) of the spike protein is known to enhance the binding affinity of the spike protein to human ACE2 receptor and increase the resistance against several neutralizing monoclonal antibodies (Thomson et al., 2021). In addition, three of those genomes showed the S:H69/V70 mutation, which often co-occurs in the RBM with amino acid replacements such as N439K. This also is shown to associate with increased infectivity (Kemp et al., 2021). None of these were identified within the community. The other more frequent mutations were T166I in NSP2, L37F in NSP6, and T205I in N-protein. The L37F mutation in NSP6 is thought to render the NSP6 protein less stable and therefore, compromise the function of NSP6 (Wang et al., 2020). The other mutations have been frequently reported in many other SARS-CoV-2 lineages (Kaushal et al., 2020) while the other changes that were detected in the amino acids have not been associated with increased or reduced virulence.

Since the emergence of the “second wave” of SARS-CoV-2 infections in early October 2020, all repatriation from overseas was stopped for a few months and subsequently, restarted in December 2020. Along with this, viruses of many lineages were identified within the quarantine centres where overseas returnees were housed. Importantly, the B.1.1.7 variant, which has been associated with higher transmissibility (Volz et al., 2021b) was initially identified within these quarantine centres, but later from the community from samples sequenced from April 2021. The introduction and spread of the B.1.1.7 led to an exponential rise in the number of cases, along with the number of deaths (Ministry of Health, 2021c). Despite strict quarantine for returning travellers, where several imported B.1.1.7 cases were detected, a period of relative quiescence has been followed by an explosive increase in cases across the country. During a period of 1 month, it appears that the B.1.1.7 lineage had almost completely replaced the circulating B.1.411 due to is higher transmissibility (Volz et al., 2021b). The introduction of B.1.1.7 has also resulted in higher case fatality rates (CFRs). For instance, the COVID-19 until to March 31 was 0.63%, whereas since the April 1 the CFRs has risen to 0.89%, and is 1.09% during May 15–21 (Ministry of Health, 2021c). The rise in the CFRs could be due to increase in the number of patients requiring health care or possibly due to the increase in viral virulence (Davies et al., 2021).

In summary, the viruses identified in March 2020, could be due to multiple introductions from overseas. The large outbreak that started in early October, appears to be due to spread of a single virus lineage, B.1.411 until to end of March 2021. The current exponential rise in case numbers appears to be due to the introduction of B.1.1.7 into the community, rapidly displacing the previous circulating B.1.411. As SARS-CoV-2 vaccine rollout commences in Sri Lanka, ongoing genomic surveillance for variants of concern will be vital.

## Data Availability Statement

The datasets presented in this study can be found in online repositories. The names of the repository/repositories and accession number(s) can be found in the article/Supplementary Material.

## Ethics Statement

The studies involving human participants were reviewed and approved by Ethics Review Committee, University of Sri Jayewardenepura. Written informed consent for participation was not required for this study in accordance with the national legislation and the institutional requirements.

## Author Contributions

CJ, TS, and GM: conceptualization. DJ, DA, TJ, LG, and DP: experiments. CJ, AW, MK, and GM: patient data acquisition. DR, NH, BL, and MP: data analysis. DR, TS, and GM: writing the manuscript. TS, GM, and GO: review and editing the manuscript. CJ, GM, and GO: funding. All authors contributed to the article and approved the submitted version.

## Conflict of Interest

The authors declare that the research was conducted in the absence of any commercial or financial relationships that could be construed as a potential conflict of interest.

## Publisher’s Note

All claims expressed in this article are solely those of the authors and do not necessarily represent those of their affiliated organizations, or those of the publisher, the editors and the reviewers. Any product that may be evaluated in this article, or claim that may be made by its manufacturer, is not guaranteed or endorsed by the publisher.

## Footnotes

1
https://github.com/W-L/ProblematicSites_SARS-CoV-2
