# Erratum: Genomic and Epidemiological Analysis of SARS-CoV-2 Viruses in Sri Lanka

**DOI:** 10.3389/fmicb.2022.898684

**Published:** 2022-04-11

**Authors:** Frontiers Production Office

**Affiliations:** Frontiers Media SA, Lausanne, Switzerland

**Keywords:** SARS-CoV-2, genomic sequencing, pangolin lineages, B.1.411, B.1.1.7, molecular epidemiology

Due to a production error, affiliations 4 and 5 were incorrectly published as:

^4^MRC Weatherall Institute of Molecular Medicine, National Institute of Infectious Diseases, Angoda, Sri Lanka

^5^University of Oxford, Oxford, United Kingdom

The correct affiliations 4 and 5 are:

^4^ National Institute of Infectious Diseases, Angoda, Sri Lanka

^5^ MRC Weatherall Institute of Molecular Medicine, University of Oxford, Oxford, United Kingdom

The publisher apologizes for this mistake. The original version of this article has been updated.

## Publisher's Note

All claims expressed in this article are solely those of the authors and do not necessarily represent those of their affiliated organizations, or those of the publisher, the editors and the reviewers. Any product that may be evaluated in this article, or claim that may be made by its manufacturer, is not guaranteed or endorsed by the publisher.

