# Supplementary material for: Genomic and Epidemiological Analysis of SARS-CoV-2 Viruses in Sri Lanka
Source: Front Microbiol. 2021 Sep 16;12:722838. doi: 10.3389/fmicb.2021.722838 (PMC8483294; doi:10.3389/fmicb.2021.722838)
Supplement: Supplementary file 1 [file Data_Sheet_1.ZIP › Supplementary_4_map/SL373_prop_map.pdf]

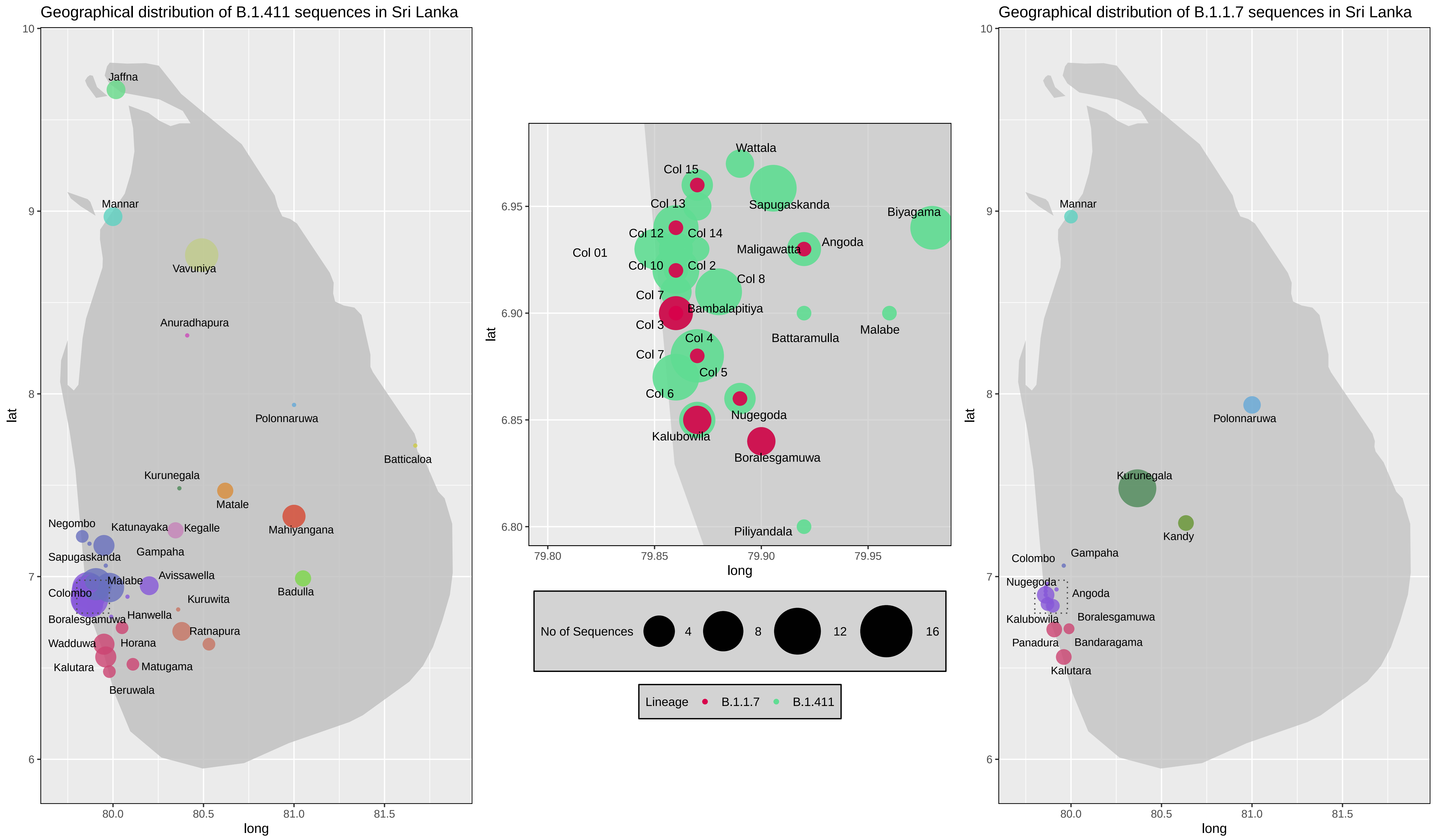
Geographical distribution of B.1.411 sequences in Sri Lanka
Jaffna
Mannar
Vavuniya
Anuradhapura
Polonnaruwa
Batticaloa
Kurunegala
Matale
Negombo
Katunayaka
Kegalle
Mahiyangana
Sapugaskanda
Gampaha
Avissawella
Malabe
Colombo
Kuruwita
Badulla
Boralesgamuwa
Hanwella
Ratnapura
Wadduwa
Horana
Kalutara
Matugama
Beruwala
lat
long
10
9
8
7
6
80.0
80.5
81.0
81.5
Wattala
Col 15
Sapugaskanda
Col 13
Biyagama
Col 12
Col 14
Angoda
Col 01
Maligawatta
Col 10
Col 2
Col 8
Col 7
Bambalapitiya
Malabe
Col 3
Battaramulla
Col 4
Col 7
Col 5
Col 6
Nugegoda
Kalubowila
Boralesgamuwa
Piliyandala
6.95
6.90
6.85
6.80
79.80
79.85
79.90
79.95
No of Sequences
4
8
12
16
Lineage
B.1.1.7
B.1.411
Geographical distribution of B.1.1.7 sequences in Sri Lanka
Mannar
Polonnaruwa
Kurunegala
Kandy
Colombo
Gampaha
Nugegoda
Angoda
Kalubowila
Boralesgamuwa
Panadura
Bandaragama
Kalutara
