# Supplementary figures and images for: Genomic and Epidemiological Analysis of SARS-CoV-2 Viruses in Sri Lanka
Source: Front Microbiol. 2021 Sep 16;12:722838. doi: 10.3389/fmicb.2021.722838 (PMC8483294; doi:10.3389/fmicb.2021.722838)

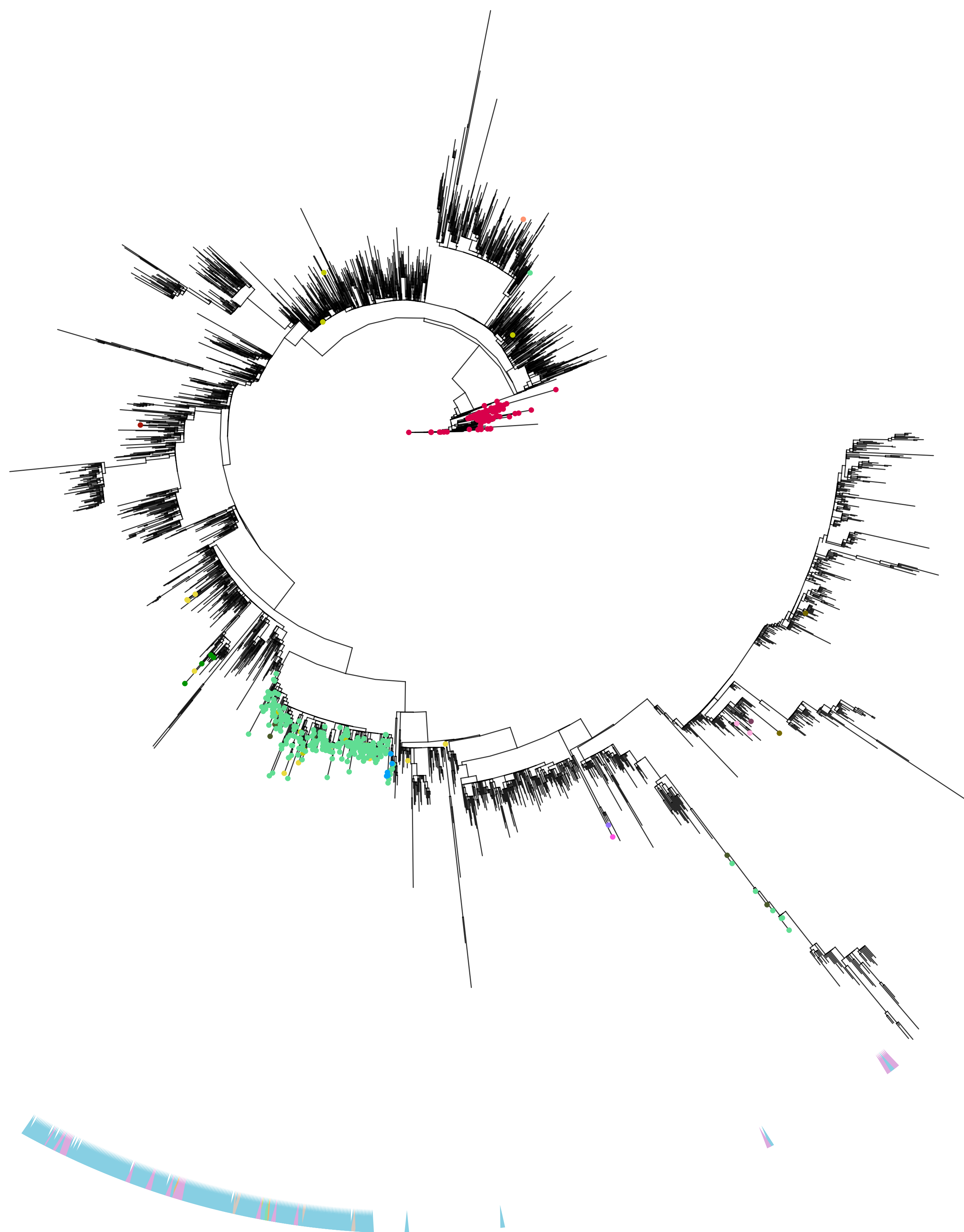

- Lineage
- B
  - B.1
  - B.1.1
  - B.1.1.25
  - B.1.1.365
  - B.1.1.7
  - B.1.258
  - B.1.351
  - B.1.411
  - B.1.428
  - B.1.525
  - B.1.617.2
  - B.4
  - B.4.7
- Outbreak
- A
  - B
  - C
  - D
  - E
  - F

Supplement: Supplementary file 1 [file Data_Sheet_1.ZIP › Supplementary_1_global_tree/SL_Global.pdf]

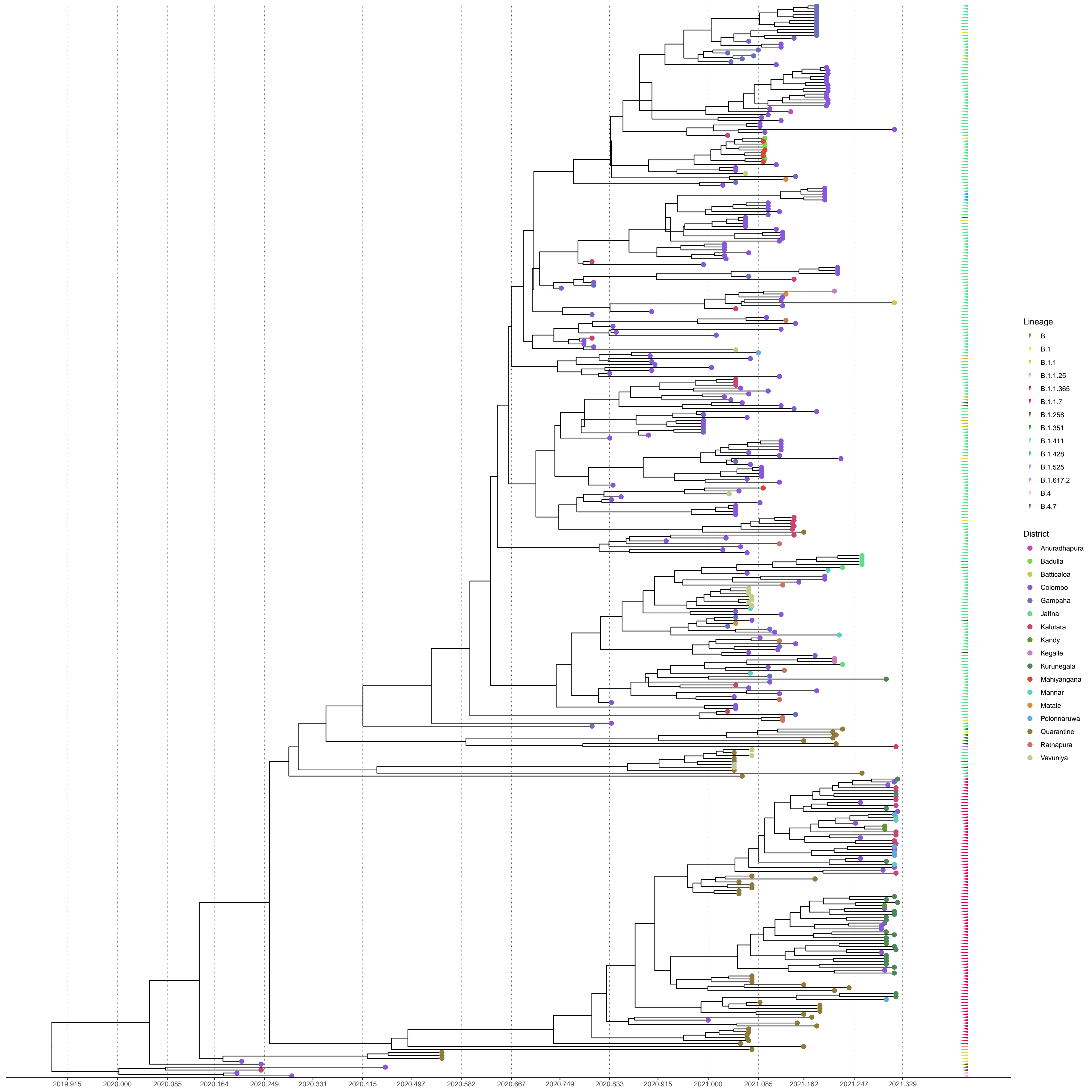

Supplement: Supplementary file 1 [file Data_Sheet_1.ZIP › Supplementary_3_clock_tree/SL_BEAST_clock_tree.pdf]
