# Supplementary material for: Genomic and Epidemiological Analysis of SARS-CoV-2 Viruses in Sri Lanka
Source: Front Microbiol. 2021 Sep 16;12:722838. doi: 10.3389/fmicb.2021.722838 (PMC8483294; doi:10.3389/fmicb.2021.722838)
Supplement: Supplementary file 2 [file Data_Sheet_2.PDF]

Supplementary Table 1. Metadata of 373 SARS-CoV2 samples sequenced in Sri Lanka between March 2020 to April 2021

Sequence_ID	GISAID_Accession ID	Outbreak Period	Pango Lineage	Clade	Collection Date	Location	Patient Gender	Patient Age	AA_Substitutions
CoV53	EPI_ISL_428671	A	B.1.1	GR	2020-03-10	Colombo	Male	58	N_R203K, N_G204R, NSP12_P323L, Spike_D614G
Cov38	EPI_ISL_428670	A	B.4	O	2020-03-16	Colombo	Male	33	NSP14_M315I, NSP2_V198I, NS3_I263M, NSP6_L37F, NSP15_S288F, N_A398S
CoV91	EPI_ISL_428672	A	B.1	G	2020-03-19	Colombo	Male	37	NSP3_M494I, NSP12_P323L, Spike_D614G, M_A2V
COV194	EPI_ISL_525478	A	B.1.1	GR	2020-03-22	Kalutara	Male	66	NS6_I60V, N_R203K, N_G204R, NSP12_P323L, Spike_D614G
CoV486	EPI_ISL_428673	A	B	O	2020-03-31	Colombo	Male	47	NS3_G251V
COV503	EPI_ISL_525479	A	B.4.7	O	2020-03-31	Kalutara	Female	53	N_P199S, NSP2_V198I, NSP2_R27C, NS8_G8stop, NS3_M260T, NSP14_D345G, NSP4_M33I, NSP6_L37F
C142	EPI_ISL_525474	B	B.4	O	2020-04-19	Colombo	Male	2	NSP14_M315I, NSP2_V198I, NS3_A98S, NS3_I263M, NS7b_H42Y, NSP6_L37F, NSP15_S288F, N_A398S
1885	EPI_ISL_525476	B	B.1	G	2020-04-28	Colombo	Female	93	NS6_I60V, NSP12_D893B, NSP3_P125L, NSP12_P323L, Spike_D614G, NSP5_V157I
7066	EPI_ISL_525481	C	B	O	2020-06-16	Colombo	Male	43	NSP2_V198I, NSP2_R27C, NSP12_D893B, E_A41T, N_S186F, Spike_T478R, NSP4_M33I, NSP6_L37F, Spike_D80Y, NSP12_D208B
KK224	EPI_ISL_525488	D	B.1	GH	2020-07-21	Quarantine	Male	37	Spike_G769V, NS3_V90F, NSP3_P74S, NS3_Y156H, NSP12_K91R, NS3_Q57H, NSP5_A234V, NSP2_T85I, NSP2_F93V, NSP12_A706T, Spike_D614G
KK230	EPI_ISL_525489	D	B.1	GH	2020-07-21	Quarantine	Male	37	NSP3_S377I, Spike_G769V, NS3_V90F, NSP3_P74S, NS3_Y156H, Spike_P1162L, NS3_Q57H, NSP5_A234V, NSP3_V50A, NSP2_T85I, NSP2_K384E, NSP12_P323L, NSP12_A706T, Spike_D614G
KK57	EPI_ISL_525486	D	B.1	GH	2020-07-21	Quarantine	Male	23	NSP3_S377I, Spike_G769V, NS3_V90F, NSP3_P74S, NS3_Y156H, NS3_Q57H, NSP5_A234V, NSP2_T85I, NSP12_A706T, Spike_D614G, N_Q418H
SL-MIN01	EPI_ISL_602564	E	B.1.411	GH	2020-10-03	Gampaha	Female	39	NSP12_D445G, NSP12_M666I, NS8_Q18stop, Spike_H1159Y, N_T205I, NSP2_T166I, NS3_Q57H, NSP2_T85I, NSP12_P323L, Spike_D614G, NSP6_L37F
SL-CMC9932	EPI_ISL_602572	E	B.1.411	O	2020-10-17	Colombo	Male	61	NSP12_M666I, Spike_H1159Y, NSP2_T166I, NS3_Q57H, NSP2_T85I, NSP12_P323L, NSP6_L37F
SL-CMC9950	EPI_ISL_602573	E	B.1.411	GH	2020-10-17	Colombo	Male	22	NSP12_M666I, Spike_H1159Y, NSP2_T166I, NS3_Q57H, NSP2_T85I, Spike_D614G, NSP6_L37F
SL-BIN33	EPI_ISL_602575	E	B.1.411	O	2020-10-22	Gampaha	Male	n/a	NSP2_ins211TSX, NSP2_E210D, NSP12_M666I, NSP2_T166I, NSP3_Y519N, Spike_L841I, NSP5_Q306R, NSP12_P323L, Spike_D614G, NSP2_S211H, NSP6_L37F
SL-BIN44	EPI_ISL_602576	E	B.1.411	O	2020-10-22	Gampaha	Male	n/a	NSP12_M666I, NS8_Q18stop, Spike_H1159Y, N_T205I, NSP2_T166I, NS3_Q57H, NSP2_T85I, NSP2_R222C, NSP12_P323L, Spike_D614G, NSP6_L37F

SL-WB04	EPI_ISL_602566	E	B.1.411	GH	2020-10-22	Beruwala	Male	n/a	NSP12_M666I, NS8_Q18stop, Spike_H1159Y, N_T205I, NSP2_T166I, NS3_Q57H, NSP10_P107del, NSP2_T85I, NSP10_V108del, NSP12_P323L, Spike_D614G
SL-WB28	EPI_ISL_602567	E	B.1.411	GH	2020-10-22	Beruwala	Male	n/a	NSP12_M666I, Spike_H1159Y, NSP2_T166I, NS3_Q57H, NSP2_T85I, NSP12_P323L, Spike_D614G, NSP6_L37F
SL-CMC11595	EPI_ISL_602565	E	B.1.411	GH	2020-10-23	Colombo	Male	31	NSP12_M666I, NS8_Q18stop, Spike_F92H, Spike_S94P, Spike_H1159Y, NSP2_T166I, NS3_Q57H, NSP2_T85I, NSP12_P323L, Spike_D614G, NSP6_L37F, Spike_A93Y
SL-CSTH3262	EPI_ISL_602568	E	B.1.411	GH	2020-10-23	Colombo	Female	79	NSP12_M666I, NS8_Q18stop, Spike_H1159Y, NSP2_T166I, NS3_Q57H, NSP2_T85I, NSP12_P323L, Spike_D614G, NSP6_L37F
SL-GQC12	EPI_ISL_602574	E	B.1.411	GH	2020-10-23	Gampaha	Male	35	NSP12_M666I, NS8_Q18stop, Spike_H1159Y, N_T205I, NSP2_T166I, NS3_Q57H, NSP2_T85I, NSP12_P323L, Spike_D614G, NSP6_L37F
cov3563	EPI_ISL_668454	E	B.1.411	GH	2020-11-02	Colombo	Male	43	NSP12_D445G, NSP12_M666I, NS8_Q18stop, Spike_H1159Y, N_T205I, NSP2_T166I, NS3_Q57H, NSP14_P203L, NSP12_P323L, Spike_D614G, NSP6_L37F
cov3576	EPI_ISL_668455	E	B.1.411	G	2020-11-02	Colombo	Male	42	NSP12_M666I, NS8_Q18stop, Spike_H1159Y, N_T205I, NSP2_T166I, NSP12_P323L, Spike_D614G
7589	EPI_ISL_668447	E	B.1.411	O	2020-11-03	Colombo	Female	54	NSP12_M666I, NS8_Q18stop, Spike_H1159Y, N_T205I, NSP2_T166I, NS3_Q57H, NSP12_P323L, Spike_D614G, NSP6_L37F
14902	EPI_ISL_668448	E	B.1.411	O	2020-11-03	Colombo	Male	46	NSP12_M666I, NS8_Q18stop, N_T205I, NSP2_T166I, Spike_D614G, NSP6_L37F
CM10	EPI_ISL_668453	E	B.1.411	G	2020-11-03	Colombo	Male	36	NSP12_M666I, Spike_H1159Y, N_T205I, NSP2_T166I, NSP12_P323L, Spike_D614G, NSP6_L37F
15372	EPI_ISL_668449	E	B.1.411	GH	2020-11-04	Colombo	Male	46	NSP12_M666I, Spike_H1159Y, N_T205I, NSP2_T166I, NS3_Q57H, NSP2_T85I, NS7a_A79V, Spike_D614G, NSP6_L37F
15386	EPI_ISL_668450	E	B.1.411	GH	2020-11-04	Colombo	Male	34	NSP12_D445G, NSP12_M666I, NS8_Q18stop, Spike_H1159Y, N_T205I, NSP2_T166I, NS3_Q57H, NS8_E106stop, Spike_D614G, NSP6_L37F
15899	EPI_ISL_668451	E	B.1.411	G	2020-11-06	Maligawatta	Male	22	NSP12_D445G, NSP12_M666I, Spike_H1159Y, N_T205I, NSP2_T166I, NSP2_T85I, NSP12_P323L, Spike_D614G, NSP6_L37F
17272	EPI_ISL_668452	E	B.1.411	O	2020-11-09	Colombo	Male	74	NSP12_M666I, NS8_Q18stop, Spike_H1159Y, N_T205I, NSP2_T166I, NS3_Q57H, Spike_D614G, NSP6_L37F, Spike_Q14H
CMC34108	EPI_ISL_792547	E	B.1.411	GH	2020-11-26	Colombo	Male	19	NSP12_M666I, NS8_Q18stop, Spike_H1159Y, N_T205I, NSP2_T166I, NS3_Q57H, Spike_D614G, N_A134V, NSP6_L37F
cov5260	EPI_ISL_792552	E	B.1.411	O	2020-11-27	Colombo	Male	45	NSP12_M666I, NS8_Q18stop, Spike_H1159Y, N_T205I, NSP2_T166I, NS3_Q57H, Spike_D614G
CMC35812	EPI_ISL_792548	E	B.1.411	GH	2020-11-28	Colombo	Female	33	NSP12_M666I, NS8_Q18stop, Spike_H1159Y, N_T205I, NS3_Q57H, NSP12_P323L, Spike_D614G

DKP18	EPI_ISL_792554	E	B.1.411	GH	2020-11-28	Colombo	Male	30	NSP2_L71F, NSP12_M666I, NS8_Q18stop, Spike_H1159Y, N_T205I, NSP2_T166I, NS3_Q57H, NSP2_T85I, NSP12_P323L, Spike_D614G, NSP6_L37F
DKP5	EPI_ISL_792553	E	B.1.411	GH	2020-11-28	Colombo	Male	36	NSP2_L71F, NSP12_M666I, Spike_H1159Y, N_T205I, NS3_Q57H, Spike_D614G
CMC37019	EPI_ISL_792549	E	B.1.411	GH	2020-11-30	Colombo	Male	36	NSP12_M666I, NS8_Q18stop, Spike_H1159Y, NS6_N39T, N_T205I, NSP2_T166I, NS3_Q57H, Spike_F186L, Spike_D614G
w65	EPI_ISL_792556	E	B.1.411	O	2020-12-07	Colombo	Male	n/a	NSP12_M666I, Spike_H1159Y, N_T205I, NSP2_T166I, NS3_Q57H, Spike_D614G
CMC60514	EPI_ISL_1717040	E	B.1.1	GH	2020-12-30	Colombo	Female	43	NSP12_D445G, NSP13_S80G, NSP12_M666I, Spike_H1159Y, NSP2_T166I, NS3_Q57H, NSP13_D260Y, NSP12_P323L, Spike_D614G, NSP6_L37F, Spike_D178G
CMC60597	EPI_ISL_1717042	E	B.1.1	GH	2020-12-30	Colombo	Female	29	NSP12_D445G, NSP13_S80G, NSP12_M666I, NS8_Q18stop, Spike_H1159Y, N_T205I, NS3_L106R, NS3_Q57H, NSP13_D260Y, NSP12_P323L, Spike_D614G, NSP6_L37F, Spike_D178G
CMC60508	EPI_ISL_1717039	E	B.1.411	GH	2020-12-30	Kahathuduwa	Male	58	NSP12_D445G, NSP12_M666I, NS8_Q18stop, NSP14_D496Y, Spike_H1159Y, Spike_E154K, N_T205I, NSP2_T166I, NS3_Q57H, NSP2_T85I, Spike_D253N, NSP12_P323L, Spike_D614G, NSP6_L37F
CMC60513	EPI_ISL_792550	E	B.1.411	O	2020-12-30	Colombo	Female	76	NSP13_S80G, NSP12_M666I, NS8_Q18stop, Spike_H1159Y, N_T205I, NSP2_T166I, NS3_L106R, NS3_Q57H, NSP13_D260Y, Spike_D614G, Spike_D178G
CMC60515	EPI_ISL_1717041	E	B.1.411	G	2020-12-30	Colombo	Female	12	NSP12_D445G, NSP13_S80G, NSP12_M666I, NS8_Q18stop, Spike_H1159Y, N_T205I, NSP2_T166I, NS3_L106R, NSP2_T85I, NSP13_D260Y, NSP12_P323L, Spike_D614G, NSP6_L37F, Spike_D178G
NP47	EPI_ISL_1717092	E	B.1.411	O	2020-12-30	Negombo	Male	n/a	NSP12_D445G, NSP12_M666I, NS8_Q18stop, Spike_H1159Y, N_T205I, NSP2_T166I, NSP2_T85I, NSP12_P323L, Spike_D614G, N_A134V, NSP6_L37F, NSP3_L1096I
NP55	EPI_ISL_1717093	E	B.1.411	GH	2020-12-30	Negombo	Male	n/a	NSP12_D445G, NSP12_M666I, NS8_Q18stop, Spike_H1159Y, N_T205I, NSP2_T166I, NS3_Q57H, NSP12_P323L, Spike_D614G, N_A134V, NSP6_L37F, NSP3_L1096I
ECB1	EPI_ISL_792555	E	B.1.1.7	GR	2021-01-02	Colombo	Male	33	NS8_Q27stop, NSP3_T183I, Spike_T716I, NSP6_S106del, N_R203K, Spike_A570D, Spike_N501Y, NSP3_I1412T, NS8_R52I, Spike_P681H, NSP3_L1700F, NSP3_P402L, NSP3_T1501I, NSP6_G107del, NSP3_A890D, Spike_D1118H, NSP6_F108del, NS8_Y73C, N_G204R, NSP12_P323L, Spike_D614G, N_D3L, Spike_S982A, N_S235F
CMC64284	EPI_ISL_792551	E	B.1.411	GH	2021-01-04	Colombo	Female	49	NSP12_M666I, NS8_Q18stop, Spike_H1159Y, N_T205I, NSP2_T166I, NS3_Q57H, M_K14I, M_L16Q, M_K15M, NSP12_P323L, Spike_D614G

CMC66839	EPI_ISL_1717043	E	B.1.411	G	2021-01-07	Colombo	Male	54	NSP12_M666I, Spike_H1159Y, N_T205I, NSP2_T166I, NSP2_T85I, NSP12_P323L, NSP3_Q1884H, Spike_D614G, NSP6_L37F
CMC68909	EPI_ISL_1717044	E	B.1.411	GH	2021-01-11	Colombo	Female	48	NSP12_M666I, NS8_Q18stop, Spike_H1159Y, N_T205I, NSP2_T166I, NS3_Q57H, NSP2_T85I, NSP12_P323L, NSP3_Q1884H, Spike_D614G, NSP6_L37F
CoV7137	EPI_ISL_872478	E	B.1.411	GH	2021-01-11	Colombo	Female	61	NSP12_M666I, NS8_Q18stop, Spike_H1159Y, N_T205I, NSP2_T166I, NS3_Q57H, N_M210V, NSP12_P323L, Spike_D614G, NSP6_L37F
N22	EPI_ISL_1717091	E	B.1.411	O	2021-01-11	Colombo	Male	32	NSP12_D445G, NS7a_G38stop, NSP12_M666I, NS8_Q18stop, Spike_H1159Y, NSP4_A380V, NSP2_T166I, NS3_Q57H, NSP12_P323L, NS8_Q72H, Spike_D614G, NSP6_L37F
CMC69934	EPI_ISL_1717046	E	B.1.1	GH	2021-01-12	Colombo	Female	44	NSP12_D445G, NSP12_M666I, NS8_Q18stop, Spike_H1159Y, NS3_W131C, N_T205I, NSP2_T166I, NS3_Q57H, NSP2_T85I, NSP12_P323L, Spike_D614G, NSP6_L37F, Spike_C1247F
CMC69602	EPI_ISL_1717045	E	B.1.411	GH	2021-01-12	Colombo	Male	77	NSP12_D445G, NSP12_M666I, NS8_Q18stop, Spike_H1159Y, N_T205I, NSP2_T166I, NS3_Q57H, NSP2_T85I, NSP12_P323L, Spike_D614G, NSP6_L37F
P2	EPI_ISL_1717099	E	B.1.411	GH	2021-01-12	Colombo	Male	n/a	NSP12_D445G, NSP12_M666I, NS8_Q18stop, Spike_H1159Y, NSP3_M1556L, N_T205I, NSP2_T166I, NS3_Q57H, NSP2_T85I, NS7a_I3F, NSP12_P323L, Spike_D614G, NSP6_L37F, Spike_A222V
P4	EPI_ISL_1717100	E	B.1.411	GV	2021-01-12	Colombo	Male	n/a	NSP12_D445G, NSP12_M666I, NS8_Q18stop, Spike_H1159Y, NSP3_M1556L, N_T205I, NS7a_I3F, NSP12_P323L, Spike_D614G, NSP6_L37F, Spike_A222V
P8	EPI_ISL_862725	E	B.1.411	GH	2021-01-12	Colombo	Male	n/a	NSP12_M666I, NS8_Q18stop, Spike_H1159Y, N_T205I, NSP2_T166I, NS3_Q57H, NSP2_T85I, NSP12_P323L, Spike_D614G, NSP6_L37F, Spike_A222V
CMC70666	EPI_ISL_1717047	E	B.1.411	GH	2021-01-13	Colombo	Male	28	NSP14_Y361C, NSP6_T172A, NSP12_M666I, Spike_H1159Y, N_T205I, NSP2_T166I, NS3_Q57H, NSP6_ins171MTARTVYDDG, NS3_T175I, NSP12_P323L, Spike_D614G, NSP6_L37F
CMC70852	EPI_ISL_872394	E	B.1.411	GH	2021-01-13	Colombo	Female	34	NSP2_M404V, NSP12_M666I, NS8_Q18stop, Spike_H1159Y, N_T205I, NSP2_T166I, NS3_Q57H, NSP2_T85I, NSP7_A80V, Spike_L1063F, NSP12_P323L, Spike_D614G, NSP6_L37F
ABG16	EPI_ISL_862718	E	B.1.411	O	2021-01-14	Gampaha	Female	46	NSP12_M666I, NS8_Q18stop, Spike_H1159Y, N_T205I, NSP2_T166I, NS3_Q57H, Spike_A684V, Spike_D614G, NSP6_L37F, NSP3_P654S
ABG34	EPI_ISL_862719	E	B.1.411	G	2021-01-14	Gampaha	Male	34	NSP12_D445G, N_K373R, NSP12_M666I, Spike_T716I, NS8_Q18stop, NSP3_I617V, Spike_H1159Y, N_T205I, NSP2_T166I, Spike_D614G, NSP6_L37F

CA78693	EPI_ISL_1717026	E	B.1.411	GH	2021-01-14	Horana	Male	25	NSP12_M666I, NS8_Q18stop, Spike_H1159Y, N_T205I, NSP2_T166I, NS3_Q57H, NSP2_T85I, NS3_K67R, NSP12_P323L, NSP2_V308L, Spike_D614G, NSP6_L37F
CA78694	EPI_ISL_1717027	E	B.1.411	G	2021-01-14	Horana	Male	29	NSP12_D445G, NSP12_M666I, Spike_H1159Y, N_T205I, NSP2_T166I, NSP12_P323L, Spike_D614G, NSP6_L37F
MVP03	EPI_ISL_872567	E	B.1.411	O	2021-01-15	Vavuniya	Male	20	NSP3_P985L, NSP12_M666I, NS8_Q18stop, Spike_H1159Y, N_T205I, NSP2_T166I, NS3_Q57H, NSP2_T85I, NSP9_T24I, NSP12_P323L, Spike_D614G, NSP6_L37F
CA79689	EPI_ISL_1717028	E	B.1.411	GH	2021-01-16	Gampaha	Female	32	NSP12_M666I, NS8_Q18stop, Spike_H1159Y, N_T205I, NS3_Q57H, NSP12_P323L, Spike_A684V, Spike_D614G, Spike_T1238S, NSP6_L37F, NSP3_P654S
CMC72385	EPI_ISL_1718051	E	B.1.411	GH	2021-01-16	Colombo	Male	24	NSP14_R213H, NSP14_R212H, NSP14_L209del, NSP12_M666I, NSP14_D211del, NSP14_T215K, Spike_H1159Y, N_T205I, NSP2_T166I, NSP14_C210del, NSP14_C208del, NSP12_P323L, Spike_D614G, NSP6_L37F, NSP14_F217del
VQC30	EPI_ISL_862727	E	B.1.258	G	2021-01-18	Vavuniya	Female	31	NSP13_H290Y, NSP9_M101I, NSP12_V720I, NSP6_E195D, NSP12_P323L, Spike_D614G
VQC32	EPI_ISL_862716	E	B.1.258	O	2021-01-18	Colombo	Female	42	Spike_H69del, NSP2_T175N, NSP13_H290Y, NSP13_A598S, NSP9_M101I, NSP12_V720I, Spike_V70del, NSP14_S134F, NSP6_E195D, Spike_D614G
CMC72613	EPI_ISL_1717048	E	B.1.411	G	2021-01-18	Colombo	Male	27	NSP12_M666I, Spike_H1159Y, N_T205I, Spike_D614G, NSP6_L37F
CV80518	EPI_ISL_1717070	E	B.1.411	G	2021-01-18	Quarantine	Female	42	Spike_H69del, NSP13_H290Y, NSP13_A505P, NSP3_I1683T, Spike_N439K, NSP13_A598S, NSP9_M101I, NSP12_V720I, Spike_V70del, NSP14_S134F, NSP6_E195D, NSP12_P323L, Spike_D614G, NS7b_A15S
CV80604	EPI_ISL_1717071	E	B.1.411	G	2021-01-18	Quarantine	Female	34	Spike_H69del, NSP13_H290Y, NSP3_I1683T, NSP9_M101I, NSP12_V720I, Spike_V70del, NSP6_E195D, NSP12_P323L, Spike_D614G
CV80630	EPI_ISL_1717072	E	B.1.411	G	2021-01-18	Quarantine	Female	45	Spike_H69del, NSP13_H290Y, NSP13_A505P, Spike_N439K, NSP13_A598S, NSP9_M101I, NSP12_V720I, Spike_V70del, NSP14_S134F, NSP6_E195D, NSP12_P323L, Spike_D614G
VQC14	EPI_ISL_862726	E	B.1.411	G	2021-01-18	Vavuniya	Female	53	NSP13_H290Y, NSP13_A505P, NSP13_A598S, NSP9_M101I, NSP12_V720I, NSP14_S134F, NSP6_E195D, Spike_D614G
CA81471	EPI_ISL_1717031	E	B.1.1	GH	2021-01-19	Awissawella	Female	40	NSP12_D445G, NSP12_M666I, NS8_Q18stop, Spike_H1159Y, N_T205I, NSP2_T166I, NS3_Q57H, NS3_V13L, NSP2_T85I, NSP3_V929I, NSP12_P323L, Spike_D614G, NSP6_L37F
CA81615	EPI_ISL_1717032	E	B.1.1	G	2021-01-19	Colombo	Male	27	NSP12_D445G, NSP12_M666I, NS8_Q18stop, NSP14_P203F, Spike_H1159Y, NSP2_T166I, NSP12_P323L, Spike_D614G, NSP2_S138L, NSP6_L37F
A7528	EPI_ISL_862717	E	B.1.411	O	2021-01-19	Colombo	Male	30	NSP12_M666I, NS8_Q18stop, Spike_H1159Y, N_T205I, NSP2_T166I, NS3_Q57H, NSP12_P323L, NSP3_A488V, Spike_D614G, NSP6_L37F, Spike_G1124V

CA81375	EPI_ISL_1718049	E	B.1.411	O	2021-01-19	Awissawella	Male	21	NSP12_D445G, NSP12_M666I, NS8_Q18stop, Spike_H1159Y, NSP2_T166I, NS3_V13L, NSP2_T85I, NSP3_V929I, NSP12_P323L, Spike_D614G, NSP6_L37F
CA81378	EPI_ISL_1718050	E	B.1.411	GH	2021-01-19	Awissawella	Male	28	NSP12_D445G, NSP12_M666I, NS8_Q18stop, Spike_H1159Y, NSP2_T166I, NS3_Q57H, NSP12_P323L, NSP3_A488V, Spike_D614G, NSP6_L37F, Spike_G1124V
CA81381	EPI_ISL_1717029	E	B.1.411	GH	2021-01-19	Awissawella	Male	29	NSP12_M666I, NS8_Q18stop, Spike_H1159Y, N_T205I, NSP2_T166I, NS3_Q57H, NSP12_P323L, NSP3_A488V, Spike_D614G, NSP6_L37F, Spike_G1124V
CA81465	EPI_ISL_1717030	E	B.1.411	O	2021-01-19	Awissawella	Male	27	NSP12_D445G, NSP12_M666I, NS8_Q18stop, Spike_H1159Y, N_T205I, NSP2_T166I, NS3_Q57H, NSP12_P323L, Spike_D614G, NSP6_L37F, Spike_G1124V
CMC73322	EPI_ISL_1717049	E	B.1.411	GH	2021-01-19	Piliyandala	Male	70	NSP12_D445G, NSP12_M666I, NS8_Q18stop, Spike_H1159Y, N_T205I, NSP2_T166I, NS3_Q57H, NSP7_V58A, NSP12_P323L, Spike_D614G, NSP6_L37F
CMC73331	EPI_ISL_1717050	E	B.1.411	GH	2021-01-19	Gampaha	Male	63	NSP12_M666I, NS8_Q18stop, Spike_H1159Y, N_T205I, NSP2_T166I, NS3_Q57H, NSP2_T85I, NSP12_P323L, NSP3_Q1884H, Spike_D614G, NSP6_L37F, NSP6_V149F, Spike_D80Y
CMC73350	EPI_ISL_1717051	E	B.1.411	G	2021-01-19	Colombo	Male	42	NSP12_D445G, NSP12_M666I, NSP15_S308Y, Spike_H1159Y, N_T205I, NSP2_T166I, NSP7_V58A, NSP12_P323L, Spike_D614G, NSP6_L37F
CoV7350	EPI_ISL_1717067	E	B.1.411	GH	2021-01-19	Gampaha	Female	37	NSP12_D445G, N_K373R, NSP12_M666I, Spike_T716I, NS8_Q18stop, Spike_H1159Y, N_T205I, NS3_Q57H, NSP12_P323L, Spike_D614G, NSP6_L37F
CoV7356	EPI_ISL_1718055	E	B.1.411	G	2021-01-19	Gampaha	Female	26	NSP12_D445G, NSP12_M666I, NS8_Q18stop, Spike_H1159Y, N_T205I, NSP2_T166I, NS3_L108F, NS8_R52I, NSP12_P323L, Spike_D614G, NSP6_L37F, Spike_S640F
ING2052	EPI_ISL_1717084	E	B.1.411	O	2021-01-19	Kalutara	Male	44	NSP12_D445G, NSP12_M666I, NS8_Q18stop, Spike_H1159Y, N_T205I, NSP2_T166I, NS3_Q57H, NSP2_T85I, NSP12_P323L, Spike_D614G, NSP6_L37F
ING2084	EPI_ISL_862720	E	B.1.411	GH	2021-01-19	Kalutara	Male	24	N_D128Y, NSP12_M666I, Spike_H1159Y, N_T205I, NSP2_T166I, NSP3_L1328F, NS3_Q57H, Spike_D614G, NSP6_L37F, NSP2_E201A
M151	EPI_ISL_862721	E	B.1.411	O	2021-01-19	Matale	Female	n/a	Spike_P1263L, N_K373R, NSP12_M666I, Spike_T716I, NS8_Q18stop, Spike_H1159Y, N_T205I, NSP2_T166I, NS3_Q57H, NS8_V62L, Spike_D614G, NSP6_L37F
MAT36	EPI_ISL_1717086	E	B.1.411	GH	2021-01-19	Kalutara	Male	32	NSP12_D445G, NSP3_A1311V, NSP12_M666I, NS8_Q18stop, NSP4_A231V, Spike_H1159Y, NSP2_T166I, NS3_Q57H, NSP12_P323L, Spike_D614G
MAT38	EPI_ISL_862724	E	B.1.411	GH	2021-01-19	Kalutara	Male	35	NSP12_M666I, NS8_Q18stop, Spike_H1159Y, N_T205I, NSP2_T166I, NS3_Q57H, NSP12_P323L, Spike_D614G, NSP6_L37F

MAT39	EPI_ISL_1718059	E	B.1.411	O	2021-01-19	Kalutara	Male	26	NSP3_A1311V, NSP12_M666I, Spike_H1159Y, N_T205I, NSP12_P323L, Spike_D614G, NSP6_L37F
MAT43	EPI_ISL_1717087	E	B.1.411	GH	2021-01-19	Kalutara	Male	40	NSP12_D445G, NSP3_A1311V, NSP12_M666I, NS8_Q18stop, Spike_H1159Y, N_T205I, NSP2_T166I, NS3_Q57H, NSP12_P323L, Spike_D614G, NSP6_L37F
MVP46	EPI_ISL_872568	E	B.1.411	GH	2021-01-19	Vavuniya	Male	44	NSP12_M666I, NS8_Q18stop, Spike_H1159Y, N_T205I, NSP2_T166I, NS3_Q57H, NSP2_T85I, NSP3_S1443F, NSP12_P323L, Spike_D614G, NSP6_L37F
QA31	EPI_ISL_1717101	E	B.1.1.7	O	2021-01-21	Quarantine	Female	47	Spike_H69del, NS8_Q27stop, NSP3_T183I, NS8_K68stop, NSP6_S106del, N_R203K, Spike_A570D, Spike_N501Y, NSP3_I1412T, NS8_R52I, Spike_P681H, Spike_Y144del, NSP6_G107del, NSP3_A890D, Spike_D1118H, NSP6_F108del, NS8_Y73C, N_G204R, Spike_V70del, NSP12_P323L, Spike_D614G, N_D3L, Spike_S982A
QA71	EPI_ISL_1717102	E	B.1.1.7	GR	2021-01-21	Quarantine	Female	46	NS8_Q27stop, NSP3_T183I, NSP3_A890D, NSP6_G107del, Spike_T716I, NS8_K68stop, NSP6_S106del, N_R203K, Spike_D1118H, NSP6_F108del, NS8_Y73C, N_G204R, NSP3_I1412T, NS8_R52I, NSP12_P323L, Spike_P681H, Spike_D614G, Spike_Y144del, N_D3L, Spike_S982A, N_S235F
QA72	EPI_ISL_1717103	E	B.1.1.7	GRY	2021-01-21	Quarantine	Female	29	Spike_H69del, NS8_Q27stop, NSP3_T183I, Spike_T716I, NS8_K68stop, NSP6_S106del, N_R203K, Spike_A570D, Spike_N501Y, NSP3_I1412T, NS8_R52I, Spike_P681H, Spike_Y144del, NSP6_G107del, NSP3_A890D, Spike_D1118H, NSP6_F108del, NS8_Y73C, N_G204R, Spike_V70del, NSP12_P323L, Spike_D614G, N_D3L, Spike_S982A
CMC74873	EPI_ISL_1718052	E	B.1.411	O	2021-01-21	Colombo	Male	46	NSP6_L33M, NSP6_ins35VF, NSP12_M666I, NS8_Q18stop, Spike_H1159Y, N_T205I, NSP2_T166I, NSP6_F34V, NSP6_W31Y, NS3_Q57H, NSP2_T85I, NSP6_Q30E, NSP12_P323L, Spike_D614G, NSP6_L37F, NSP6_T29P
NR5	EPI_ISL_1717098	E	B.1.1.7	GRY	2021-01-22	Quarantine	n/a	n/a	Spike_H69del, NS3_L15F, NS8_Q27stop, NSP3_T183I, NSP6_S106del, N_R203K, Spike_A570D, NSP13_K460R, NSP4_F17L, Spike_N501Y, NSP3_I1412T, NS8_R52I, Spike_Y144del, NSP3_A890D, NSP6_G107del, Spike_D1118H, NSP6_F108del, NS8_Y73C, N_G204R, Spike_V70del, NSP12_P323L, Spike_D614G, N_D3L, Spike_S982A, N_S235F
CMC75126	EPI_ISL_1717052	E	B.1.411	O	2021-01-22	Maligawatta	Male	51	NSP12_D445G, NSP12_M666I, Spike_H1159Y, N_T205I, NSP2_T166I, NS3_Q57H, NSP12_Q822H, NS3_T175I, NS3_E102K, NSP12_P323L, Spike_D614G, NSP6_L37F, Spike_T76I
CMC75184	EPI_ISL_1717053	E	B.1.411	GH	2021-01-22	Colombo	Female	55	NSP12_D445G, NSP12_M666I, NS8_Q18stop, Spike_H1159Y, N_T205I, NS3_Q57H, NSP12_P323L, Spike_D614G, NSP6_L37F

CA85022	EPI_ISL_1717033	E	B.1.1	GH	2021-01-23	Gampaha	Male	33	NSP12_D445G, NSP12_M666I, Spike_H1159Y, N_T205I, NSP2_T166I, NS3_Q57H, NSP12_P323L, Spike_A684V, Spike_D614G, NSP6_L37F, NSP3_P654S
CMC75810	EPI_ISL_1717054	E	B.1.258	G	2021-01-23	Colombo	Male	22	NSP12_D445G, NSP12_M666I, Spike_T240I, Spike_H1159Y, N_T205I, NSP2_T166I, NSP2_T85I, NSP14_S503L, NSP12_P323L, Spike_D614G, NSP6_L37F
NR1	EPI_ISL_1717094	E	B.1.411	G	2021-01-23	Quarantine	Female	67	N_P199S, NSP3_P1261S, NS3_G174V, NSP14_P203L, NSP3_S1206L, NS3_Y264H, N_V72I, NSP12_P323L, Spike_D614G, NSP2_V447F, Spike_T76I
CMC76417	EPI_ISL_1717056	E	B.1	GH	2021-01-25	Colombo	Female	42	NSP12_M666I, NS8_Q18stop, Spike_H1159Y, N_T205I, NSP2_T166I, NSP2_L410F, NS3_Q57H, Spike_L5F, Spike_D614G, NSP6_L37F
CMC76300	EPI_ISL_1717055	E	B.1.258	GH	2021-01-25	Colombo	Female	35	NSP12_M666I, NS8_Q18stop, Spike_H1159Y, N_T205I, NSP2_T166I, NSP2_L410F, NS3_Q57H, Spike_L5F, NSP2_T85I, Spike_D614G, NSP6_L37F
CMC76418	EPI_ISL_1717057	E	B.1.411	GH	2021-01-25	Colombo	Female	60	NSP12_D445G, NSP12_M666I, NS8_Q18stop, Spike_H1159Y, N_T205I, NSP2_L410F, NS3_Q57H, Spike_L5F, NSP2_T85I, NSP12_P323L, Spike_D614G, NSP6_L37F
CMC76424	EPI_ISL_1717058	E	B.1.411	GH	2021-01-25	Colombo	Female	44	NSP12_D445G, NSP12_M666I, NS8_Q18stop, Spike_H1159Y, N_T205I, NSP2_T166I, NS3_Q57H, Spike_L5F, NSP2_T85I, NSP12_P323L, Spike_D614G, NSP6_L37F
CV85548	EPI_ISL_1717073	E	B.1.411	GH	2021-01-25	Vavuniya	Male	36	NSP12_M666I, NS8_Q18stop, Spike_H1159Y, N_T205I, NS3_Q57H, NS3_V13L, NSP2_T85I, NSP4_C296F, NSP12_P323L, Spike_D614G, NSP14_S255I, NSP6_L37F, Spike_V1176F
NR2	EPI_ISL_1717095	E	B.1.1.7	GRY	2021-01-26	Quarantine	Male	36	Spike_H69del, NS8_Q27stop, NSP3_T183I, Spike_T716I, NSP6_S106del, N_R203K, Spike_N501Y, NSP3_I1412T, NS8_R52I, Spike_P681H, NS7a_Q62stop, NSP6_G107del, NSP3_A890D, Spike_D1118H, NSP6_F108del, NS8_Y73C, N_G204R, Spike_V70del, NS3_T151I, Spike_N149del, NSP12_P323L, Spike_D614G, N_D3L, Spike_S982A, N_S235F
NR3	EPI_ISL_1717096	E	B.1.1.7	O	2021-01-26	Quarantine	n/a	43	Spike_H69del, NS8_Q27stop, NSP3_T183I, NSP6_S106del, N_R203K, Spike_A570D, Spike_L5F, Spike_N501Y, NSP3_I1412T, NS8_R52I, Spike_P681H, Spike_Y144del, NS7a_Q62stop, NSP3_A890D, NSP6_G107del, Spike_D1118H, NSP6_F108del, NS8_Y73C, N_G204R, Spike_V70del, NS3_T151I, NSP12_P323L, Spike_D614G, N_D3L, Spike_S982A, N_S235F
CMC76839	EPI_ISL_1717059	E	B.1.411	O	2021-01-26	Colombo	Male	38	NSP13_S80G, NSP12_M666I, NS8_Q18stop, Spike_H1159Y, N_T205I, NSP2_T166I, NS3_L106R, NS3_Q57H, NSP12_P323L, Spike_D614G, NSP6_L37F, Spike_D178G, NSP4_L353F

CMC76926	EPI_ISL_1717060	E	B.1.411	G	2021-01-26	Colombo	Female	57	NSP12_D445G, NS8_F120V, NSP12_M666I, Spike_T33S, NS8_I121L, NS8_Q18stop, NSP9_V76A, Spike_H1159Y, N_T205I, NSP12_P323L, Spike_D614G
CMC77019	EPI_ISL_1717061	E	B.1.411	G	2021-01-26	Colombo	Female	62	NSP12_D445G, NSP12_M666I, NS8_Q18stop, N_T205I, NSP2_T166I, NSP12_P323L, NSP5_K90R, Spike_D614G, NSP6_L37F
CMC77196	EPI_ISL_1717062	E	B.1.411	O	2021-01-26	Colombo	Male	28	NSP2_L550I, N_M234V, NSP12_M666I, NS8_Q18stop, Spike_H1159Y, N_T205I, NSP2_T166I, NS3_Q57H, NSP12_P323L, Spike_D614G, NSP6_L37F, Spike_A783S
CVQ87412	EPI_ISL_1717081	E	B.1.1.7	GRY	2021-01-27	Quarantine	Female	33	Spike_H69del, NS8_Q27stop, NSP3_T183I, NSP6_S106del, N_R203K, Spike_A570D, Spike_L5F, NSP3_I1412T, NS8_R52I, Spike_P681H, Spike_Y144del, NS7a_Q62stop, NSP3_A890D, NSP6_G107del, Spike_D1118H, NSP6_F108del, NS8_Y73C, N_G204R, Spike_V70del, NS3_T151I, NSP12_P323L, Spike_D614G, N_D3L, Spike_S982A, N_S235F
CVQ87413	EPI_ISL_1717082	E	B.1.1.7	GRY	2021-01-27	Quarantine	Female	3	Spike_H69del, NS8_Q27stop, NSP3_T183I, NSP6_S106del, N_R203K, Spike_A570D, Spike_L5F, Spike_N501Y, NSP3_I1412T, NS8_R52I, Spike_Y144del, NS3_G100C, NS7a_Q62stop, NSP3_A890D, NSP6_G107del, Spike_D1118H, NSP6_F108del, NS8_Y73C, N_G204R, Spike_V70del, NS3_T151I, NSP12_P323L, Spike_D614G, N_D3L, Spike_S982A, N_S235F
CVQ87434	EPI_ISL_1717083	E	B.1.1.7	O	2021-01-27	Quarantine	Female	56	Spike_H69del, NS8_Q27stop, NSP3_T183I, Spike_T716I, NSP6_S106del, N_R203K, Spike_A570D, Spike_L5F, Spike_N501Y, NSP3_I1412T, NS8_R52I, Spike_P681H, Spike_Y144del, NS7a_Q62stop, NSP6_G107del, NSP3_A890D, Spike_D1118H, NSP6_F108del, NS8_Y73C, N_G204R, Spike_V70del, NS3_T151I, NSP12_P323L, Spike_D614G, N_D3L, Spike_S982A, N_S235F
CMC77416	EPI_ISL_1717065	E	B.1.258	O	2021-01-27	Colombo	Male	38	NSP12_D445G, NSP12_M666I, NS8_Q18stop, Spike_H1159Y, N_T205I, NSP2_T166I, Spike_E583D, NSP2_T85I, NSP12_P323L, Spike_D614G, NSP6_L37F
CA86899	EPI_ISL_1717034	E	B.1.411	GH	2021-01-27	Gampaha	Female	25	NSP12_M666I, NS8_Q18stop, Spike_H1159Y, N_T205I, NSP2_T166I, NS3_Q57H, NSP2_T85I, Spike_D287N, NSP12_P323L, Spike_D614G
CA86905	EPI_ISL_1717035	E	B.1.411	G	2021-01-27	Gampaha	Male	27	NSP12_D445G, NSP12_M666I, NS8_Q18stop, Spike_H1159Y, N_T205I, NSP2_T85I, NSP12_P323L, Spike_A684V, Spike_D614G, NSP6_L37F, NSP3_P654S
CMC77309	EPI_ISL_1717064	E	B.1.411	O	2021-01-27	Colombo	Male	55	NSP12_D445G, NSP12_M666I, NS8_Q18stop, Spike_H1159Y, N_T205I, NSP2_T166I, NSP12_P323L, Spike_D614G
CMC77618	EPI_ISL_1718054	E	B.1.411	GH	2021-01-27	Colombo	Male	59	NSP12_M666I, NS8_Q18stop, Spike_H1159Y, N_T205I, NSP2_T166I, NS3_Q57H, NS3_L108F, NS8_R52I, NSP12_P323L, Spike_D614G, NSP6_L37F, Spike_S640F

COV7469	EPI_ISL_1717068	E	B.1.411	GH	2021-01-27	Colombo	Female	4	NSP12_M666I, NS8_Q18stop, Spike_H1159Y, N_T205I, NSP2_T166I, NS3_Q57H, NSP2_T85I, NSP16_N235R, NSP12_P323L, Spike_D614G, NSP6_L37F, NSP16_ins234MstopM
COV7478	EPI_ISL_1717069	E	B.1.411	GH	2021-01-27	Colombo	Male	31	NSP12_D445G, NSP12_M666I, NS8_Q18stop, Spike_H1159Y, NS3_W131C, N_T205I, NSP2_T166I, NS3_Q57H, NSP12_P323L, Spike_D614G, NSP6_L37F
CV86866	EPI_ISL_1717074	E	B.1.411	GH	2021-01-27	Vavuniya	Male	37	NSP12_D445G, Spike_S98P, NSP12_M666I, Spike_H1159Y, N_T205I, NSP2_T166I, NS3_Q57H, NSP9_T24I, NSP12_P323L, Spike_D614G, NSP6_L37F
CV86867	EPI_ISL_1717075	E	B.1.411	GH	2021-01-27	Vavuniya	Male	31	NSP12_D445G, Spike_S98P, NSP12_M666I, Spike_H1159Y, N_T205I, NSP2_T166I, NS3_Q57H, NSP2_T85I, N_V270L, NSP12_P323L, Spike_D614G, NSP6_L37F
CV86870	EPI_ISL_1717076	E	B.1.411	GH	2021-01-27	Vavuniya	Male	30	NSP12_D445G, Spike_S98P, NSP12_M666I, NS8_Q18stop, Spike_H1159Y, N_T205I, NS3_Q57H, NSP12_P323L, Spike_D614G, NSP6_L37F
CV86880	EPI_ISL_1717077	E	B.1.411	G	2021-01-27	Vavuniya	Male	40	NSP12_D445G, Spike_S98P, NSP12_M666I, NS8_Q18stop, Spike_H1159Y, N_T205I, NSP2_T166I, NSP12_P323L, Spike_D614G, NSP6_L37F
CMC77266	EPI_ISL_1717063	E	B.1.1	GH	2021-01-28	Colombo	Female	49	NSP12_M666I, NS8_Q18stop, Spike_H1159Y, N_T205I, NSP2_T166I, NS3_Q57H, NS3_K21N, NS7a_A79V, NSP12_P323L, Spike_D614G
CMC77485	EPI_ISL_1718053	E	B.1.411	G	2021-01-28	Colombo	Female	62	NSP12_D445G, NSP12_M666I, Spike_H1159Y, N_T205I, NSP2_T166I, NS3_L108F, NS8_R52I, NSP12_P323L, Spike_D614G, Spike_S640F
MAN3	EPI_ISL_1718058	E	B.1.411	O	2021-01-28	Mannar	Male	15	NS3_V255del, NSP12_D445G, NSP14_A425V, NSP12_M666I, Spike_H1159Y, NSP13_P504S, NSP2_T166I, NS3_W193L, NSP3_V1936S, NSP12_P323L, Spike_T63A, Spike_D614G, NSP3_V1935L, NSP6_L37F
MAN4	EPI_ISL_1717085	E	B.1.411	G	2021-01-28	Mannar	Male	22	NSP12_D445G, Spike_S98P, NSP12_M666I, NS8_Q18stop, Spike_H1159Y, N_T205I, NSP12_P323L, Spike_D614G, NSP6_L37F
NR4	EPI_ISL_1717097	E	B.1.1	GR	2021-01-29	Quarantine	n/a	n/a	M_D209Y, NS8_A55V, N_R203K, NSP4_M458I, NS3_G224C, N_G204R, NS3_F114S, NSP12_P323L, Spike_D614G, NSP3_N22D
MULQ106	EPI_ISL_1717088	E	B.1.1.7	GRY	2021-01-29	Quarantine	Male	36	Spike_H69del, NS8_Q27stop, NSP3_T183I, Spike_T716I, NS8_K68stop, NSP6_S106del, N_R203K, Spike_A570D, Spike_N501Y, NSP3_I1412T, NS8_R52I, Spike_P681H, Spike_Y144del, NSP6_G107del, NSP3_A890D, Spike_D1118H, NSP6_F108del, NS8_Y73C, N_G204R, Spike_V70del, NSP12_P323L, Spike_D614G, N_D3L, Spike_S982A

MULQ112	EPI_ISL_1717089	E	B.1.1.7	GR	2021-01-29	Quarantine	Male	41	Spike_H69del, NS8_Q27stop, NSP3_T183I, NSP3_A890D, NSP6_G107del, Spike_T716I, NS8_K68stop, NS8_Q18stop, NSP6_S106del, Spike_D1118H, NSP6_F108del, NS8_Y73C, N_G204R, Spike_V70del, Spike_N501Y, NS8_R52I, NSP12_P323L, Spike_D614G, N_D3L, N_S235F
MULQ66	EPI_ISL_1717090	E	B.1.1.7	GRY	2021-01-29	Quarantine	Male	22	Spike_H69del, NS8_Q27stop, NSP3_T183I, Spike_T716I, NS8_K68stop, NSP6_S106del, N_R203K, Spike_A570D, Spike_N501Y, NSP3_I1412T, NSP15_T33I, NS8_R52I, Spike_P681H, Spike_Y144del, NSP6_G107del, NSP3_A890D, Spike_D1118H, NSP6_F108del, NS8_Y73C, N_G204R, Spike_V70del, NSP12_P323L, Spike_D614G, N_D3L, Spike_S982A
QCF12	EPI_ISL_1717104	E	B.1.1.7	GR	2021-01-29	Quarantine	Female	38	NS8_Q27stop, NSP3_T183I, NSP3_D339G, Spike_T716I, NS8_K68stop, NSP6_S106del, N_R203K, Spike_A570D, Spike_N501Y, NSP3_I1412T, NS8_R52I, Spike_P681H, N_R195I, NSP6_G107del, NSP3_A890D, Spike_D1118H, NSP3_R407I, NSP6_F108del, NS8_Y73C, N_G204R, NSP12_P323L, Spike_D614G, N_D3L, Spike_S982A
QCF6	EPI_ISL_1717105	E	B.1.1.7	GR	2021-01-29	Quarantine	Female	56	NS8_Q27stop, NSP3_T183I, NSP3_D339G, Spike_T716I, NS8_K68stop, NSP6_S106del, N_R203K, Spike_A570D, Spike_N501Y, NSP3_I1412T, NS8_R52I, Spike_P681H, Spike_Y144del, N_R195I, NSP6_G107del, NSP3_A890D, Spike_D1118H, NSP6_F108del, NS8_Y73C, N_G204R, NSP12_P323L, Spike_D614G, Spike_S982A
QCF7	EPI_ISL_1717106	E	B.1.1.7	GRY	2021-01-29	Quarantine	Female	33	Spike_H69del, NS8_Q27stop, NSP3_T183I, Spike_T716I, NS8_K68stop, NSP6_S106del, N_R203K, Spike_A570D, Spike_N501Y, NSP3_I1412T, Spike_P681H, Spike_Y144del, N_R195I, NSP6_G107del, NSP3_A890D, Spike_D1118H, NSP3_R407I, NSP6_F108del, NS8_Y73C, N_G204R, Spike_V70del, NSP12_P323L, Spike_D614G, N_D3L, Spike_S982A, N_S235F
CMC77697	EPI_ISL_1717066	E	B.1.258	GH	2021-01-29	Colombo	Male	22	NSP12_D445G, N_K373R, NSP12_M666I, Spike_T716I, NS8_Q18stop, Spike_H1159Y, N_T205I, NSP2_T166I, NS3_Q57H, NSP12_P323L, Spike_D614G, NSP6_L37F
CV87792	EPI_ISL_1717078	E	B.1.411	G	2021-01-29	Vavuniya	Male	33	NSP3_N1680D, NSP12_D445G, Spike_S98P, NSP12_M666I, NS8_Q18stop, Spike_H1159Y, N_T205I, NSP2_T166I, NSP12_P323L, Spike_D614G, NSP6_L37F
CV87795	EPI_ISL_1717079	E	B.1.411	G	2021-01-29	Vavuniya	Male	49	NSP12_D445G, Spike_S98P, NSP12_M666I, NS8_Q18stop, Spike_H1159Y, N_T205I, NSP2_T166I, NSP9_T24I, NSP12_P323L, Spike_D614G, NSP6_L37F
CV87812	EPI_ISL_1717080	E	B.1.411	O	2021-01-29	Vavuniya	Male	35	NSP12_D445G, Spike_S98P, NSP12_M666I, NS8_Q18stop, Spike_H1159Y, N_T205I, NSP2_T166I, NSP9_T24I, NSP12_P323L

CV87923	EPI_ISL_1718056	E	B.1.411	G	2021-01-29	Vavuniya	Female	55	Spike_H69del, NSP13_H290Y, NSP13_A505P, NSP3_I1683T, Spike_N439K, NS7a_P99S, NSP13_A598S, NSP9_M101I, NSP12_V720I, Spike_V70del, NSP14_S134F, NSP6_E195D, NSP12_M380L, Spike_D614G
CV87946	EPI_ISL_1718057	E	B.1.411	O	2021-01-29	Vavuniya	Female	55	Spike_N439K, NSP9_M101I, NSP12_V720I, NSP6_E195D, Spike_D614G
CA88751	EPI_ISL_1717036	E	B.1.411	GH	2021-01-30	Gampaha	Male	53	NSP2_S378F, NSP12_D445G, NSP12_M666I, Spike_H1159Y, N_T205I, NSP2_T166I, NS3_Q57H, NSP2_T85I, NSP12_P323L, Spike_A684V, Spike_D614G, NSP6_L37F, NSP3_P654S
CA89635	EPI_ISL_1717037	E	B.1.411	O	2021-02-02	Polonnaruwa	Male	44	NSP12_M666I, NS8_Q18stop, Spike_H1159Y, N_T205I, NSP2_T166I, NS3_Q57H, NSP6_L37F
CA89803	EPI_ISL_1717038	E	B.1.411	O	2021-02-02	Gampaha	Male	36	NSP12_D445G, NSP12_M666I, NS8_Q18stop, Spike_H1159Y, N_T205I, NSP2_T166I, NS3_Q57H, NSP12_P323L, Spike_A684V, Spike_D614G, NSP6_L37F, NSP3_P654S
NR125	EPI_ISL_1233116	E	B.1.1.7	G	2021-02-03	Quarantine	Male	52	Spike_H69del, NS8_Q27stop, NSP3_T183I, Spike_T716I, NSP6_S106del, NS3_T89I, Spike_N501Y, NSP3_I1412T, NS8_R52I, Spike_P681H, NSP12_P227L, NSP3_P67L, NSP6_G107del, NSP3_A890D, NSP3_D782N, Spike_D1118H, NSP6_F108del, Spike_V70del, NSP12_P323L, NSP14_P451S, Spike_D614G, N_D3L, Spike_S982A, N_S235F
C105141	EPI_ISL_1233065	E	B.1.411	G	2021-02-03	Colombo	Male	36	NSP12_D445G, NSP2_T388I, NSP12_M666I, NS8_Q18stop, Spike_H1159Y, N_T205I, NSP2_T166I, NSP2_T85I, NSP15_T48I, NSP12_P323L, Spike_D614G, NSP6_L37F, NS3_S171L
CK105351	EPI_ISL_1233082	E	B.1.411	GH	2021-02-03	Colombo	Male	21	Spike_N679K, NSP12_D445G, N_T362I, NSP12_M666I, NS8_Q18stop, Spike_H1159Y, N_T205I, NSP2_T166I, NS3_Q57H, NSP2_T497K, NSP12_E254D, NSP12_P323L, Spike_D614G, NSP6_L37F
CK105352	EPI_ISL_1233083	E	B.1.411	O	2021-02-03	Colombo	Male	30	Spike_N679K, NSP12_D445G, N_T362I, NSP12_M666I, NS8_Q18stop, Spike_H1159Y, N_T205I, NSP2_T166I, NS3_Q57H, NSP2_T497K, NSP2_T85I, NSP12_E254D, NSP12_P323L, Spike_D614G, NSP6_L37F
CK89939	EPI_ISL_1233112	E	B.1.411	O	2021-02-03	Colombo	Male	30	NSP12_D445G, NSP12_M666I, NS8_Q18stop, Spike_H1159Y, NSP2_T166I, NS3_Q57H, Spike_L585F, NSP12_P323L, Spike_D614G, NSP6_L37F
CA90197	EPI_ISL_1233115	E	B.1.411	GH	2021-02-04	Colombo	Female	30	NSP12_M666I, NS8_Q18stop, Spike_H1159Y, N_T205I, NSP2_T166I, NS3_Q57H, N_D402Y, NSP2_T85I, NSP12_P323L, Spike_D614G, NSP6_L37F
CA90219	EPI_ISL_1233071	E	B.1.411	GH	2021-02-04	Colombo	Female	52	NSP12_D445G, NS8_F120V, NSP12_M666I, Spike_T33S, NS8_I121L, NS8_Q18stop, Spike_H1159Y, N_T205I, NSP2_T166I, NSP13_L581F, NS3_Q57H, NSP12_P323L, Spike_D614G, NSP6_L37F, NSP3_T1072I, NSP2_H208Y

CA90221	EPI_ISL_1233072	E	B.1.411	GH	2021-02-04	Colombo	Female	49	NSP12_Q292H, NSP15_A94T, NSP12_D445G, NS8_F120V, NSP12_M666I, Spike_T33S, NS8_I121L, NS8_Q18stop, Spike_H1159Y, N_T205I, NSP2_T166I, NSP13_L581F, NS3_Q57H, NSP12_P323L, Spike_D614G, NSP6_L37F
CA90222	EPI_ISL_1233073	E	B.1.411	GH	2021-02-04	Colombo	Female	37	NSP12_Q292H, NSP15_A94T, NSP12_D445G, NS8_F120V, NSP12_M666I, Spike_T33S, NS8_I121L, NS8_Q18stop, Spike_H1159Y, N_T205I, NSP2_T166I, NSP13_L581F, NS3_Q57H, NSP12_P323L, Spike_D614G, NSP6_L37F, NSP3_T1072I
CA90224	EPI_ISL_1233074	E	B.1.411	GH	2021-02-04	Colombo	Female	21	NSP12_Q292H, NSP12_D445G, NS8_F120V, NSP12_M666I, Spike_T33S, NS8_I121L, NS8_Q18stop, Spike_H1159Y, N_T205I, NSP2_T166I, NSP13_L581F, NS3_Q57H, NSP2_T85I, NSP12_P323L, Spike_D614G, NSP6_L37F, NSP2_H208Y
CA90544	EPI_ISL_1233075	E	B.1.411	GH	2021-02-05	Mahiyangana	Female	39	NS3_R30H, N_T265I, NSP6_C221F, NSP12_M666I, NS8_Q18stop, Spike_H1159Y, NSP2_T166I, NS3_Q57H, NSP12_P323L, Spike_D614G, NSP6_L37F
CA90800	EPI_ISL_1233120	E	B.1.411	O	2021-02-05	Mahiyangana	Female	41	NS3_R30H, N_T265I, NSP12_D445G, NSP6_C221F, NSP12_M666I, Spike_H1159Y, N_T205I, NSP2_T166I, NS3_Q57H, NSP12_P323L, Spike_D614G, NSP6_L37F
CA90834	EPI_ISL_1233076	E	B.1.411	G	2021-02-05	Mahiyangana	Female	45	N_T265I, NSP12_D445G, NSP6_C221F, NSP12_M666I, NS8_Q18stop, Spike_H1159Y, N_T205I, NSP2_T166I, NSP2_T85I, NSP12_P323L, Spike_D614G, NSP6_L37F
CA90907	EPI_ISL_1233059	E	B.1.411	O	2021-02-05	Mahiyangana	Female	41	NSP12_M666I, NS8_Q18stop, Spike_H1159Y, N_T205I, NSP2_T166I, NS3_Q57H, NSP2_T85I, NSP5_V261F, NSP15_V320L, NSP12_P323L, Spike_D614G
CA91156	EPI_ISL_1233057	E	B.1.411	GH	2021-02-05	Mahiyangana	Female	36	N_T265I, NSP12_D445G, NSP6_C221F, NSP12_M666I, NS8_Q18stop, Spike_H1159Y, NSP2_T166I, NS3_Q57H, NSP2_T85I, NSP12_P323L, Spike_D614G, NSP6_L37F
CA91958	EPI_ISL_1233078	E	B.1	G	2021-02-06	Badulla	Female	38	NS3_R30H, N_T265I, NSP12_D445G, NSP6_C221F, NSP12_M666I, NS8_Q18stop, Spike_H1159Y, N_T205I, NSP2_T85I, NSP12_P323L, Spike_D614G, NSP6_L37F
CA91568	EPI_ISL_1233077	E	B.1.411	GH	2021-02-06	Badulla	Female	33	NS3_R30H, N_T265I, NSP12_D445G, NSP6_C221F, NSP12_M666I, NS8_Q18stop, Spike_H1159Y, N_T205I, NSP2_T166I, NS3_Q57H, NSP2_T85I, NSP12_P323L, Spike_D614G, NSP6_L37F
CA91575	EPI_ISL_1233054	E	B.1.411	GH	2021-02-06	Badulla	Female	39	NS3_R30H, NS3_V50I, NSP12_D445G, NSP6_C221F, NSP12_M666I, NS8_Q18stop, Spike_H1159Y, N_T205I, NSP2_T166I, NS3_Q57H, NSP2_T85I, NSP12_P323L, Spike_D614G, NSP6_L37F
CA91924	EPI_ISL_1233121	E	B.1.411	GH	2021-02-06	Badulla	Female	40	NS3_R30H, N_T265I, NSP12_D445G, NSP6_C221F, NSP12_M666I, Spike_H1159Y, N_T205I, NSP2_T166I, NS3_Q57H, NSP2_T85I, NSP12_P323L, Spike_D614G, NSP6_L37F

CA91949	EPI_ISL_1233062	E	B.1.411	GH	2021-02-06	Mahiyangana	Male	47	NS3_R30H, NSP6_C221F, NSP12_M666I, NS8_Q18stop, Spike_H1159Y, N_T205I, NS3_Q57H, NSP2_T85I, NSP12_P323L, Spike_D614G, NSP6_L37F
VAC01	EPI_ISL_1233108	E	B.1.411	GH	2021-02-06	Colombo	Male	n/a	Spike_N679K, NSP12_D445G, NSP12_M666I, Spike_D936Y, NS8_Q18stop, Spike_H1159Y, N_T205I, NSP2_T166I, NS3_Q57H, NSP2_T85I, NSP12_P323L, Spike_D614G, NSP6_L37F
CA92688	EPI_ISL_1233122	E	B.1.411	GH	2021-02-07	Colombo	Female	46	NSP12_D445G, NSP12_M666I, Spike_H1159Y, NSP2_T166I, NS3_Q57H, NSP2_T85I, NSP3_A667T, NSP12_P323L, Spike_D614G, NSP6_L37F
CMC79794	EPI_ISL_1233084	E	B.1.411	GH	2021-02-08	Colombo	Male	66	NSP12_D445G, NSP12_M666I, NS8_Q18stop, NSP2_A159S, Spike_H1159Y, NS8_C83S, N_T205I, NSP2_T166I, NS3_Q57H, NSP12_P323L, Spike_D614G, NSP6_L37F
CMC80220	EPI_ISL_1233085	E	B.1.411	O	2021-02-08	Colombo	Male	27	NSP14_K349N, NSP3_A690V, NSP12_D445G, NSP12_M666I, NS8_Q18stop, Spike_H1159Y, NSP16_R86K, NSP2_T166I, NS3_Q57H, NSP12_P323L, Spike_D614G, NSP6_L37F
CMC80234	EPI_ISL_1233064	E	B.1.411	GH	2021-02-08	Colombo	Male	32	NSP4_I383F, NSP3_A690V, NSP12_D445G, NSP12_M666I, NS8_Q18stop, Spike_H1159Y, NSP16_R86K, N_T205I, NSP2_T166I, NS3_Q57H, NSP3_L1266I, NSP3_S1265del, NSP12_P323L, Spike_D614G, NSP6_L37F
CMC80235	EPI_ISL_1233118	E	B.1.411	G	2021-02-08	Colombo	Male	36	NSP4_I383F, NSP3_A690V, NSP12_D445G, NSP14_G481S, NSP12_M666I, Spike_H1159Y, NSP16_R86K, N_T205I, NSP2_T166I, NSP3_L1266I, NSP3_S1265del, NSP12_P323L, Spike_D614G, NSP6_L37F
CMC80313	EPI_ISL_1233086	E	B.1.411	G	2021-02-08	Colombo	Male	47	NSP14_K349N, NSP12_D445G, NSP12_M666I, NS8_Q18stop, NSP16_R86K, N_T205I, NSP2_T85I, NSP12_P323L, Spike_D614G, NSP6_L37F
CMC80418	EPI_ISL_1233128	E	B.1.411	O	2021-02-08	Colombo	Male	19	NSP12_D445G, NSP12_M666I, Spike_H1159Y, NSP2_T166I, NSP3_T423I, NSP14_H455Y, NSP3_H290Y, NSP12_P323L, Spike_D614G, NSP6_L37F
CMC80451	EPI_ISL_1233087	E	B.1.411	GH	2021-02-08	Colombo	Male	73	NSP12_D445G, Spike_T676A, NSP12_M666I, NS8_Q18stop, NSP10_C41S, Spike_H1159Y, N_T205I, NSP2_T166I, NS3_Q57H, NSP2_T85I, NSP12_P323L, Spike_D614G, NSP6_L37F
CA94250	EPI_ISL_1233123	E	B.1.411	G	2021-02-09	Gampaha	Male	35	NSP12_D445G, N_K373R, NSP12_M666I, Spike_T716I, Spike_H1159Y, N_T205I, NSP2_T166I, NSP2_T85I, NSP12_P323L, Spike_D614G, NSP6_L37F
CA94316	EPI_ISL_1233079	E	B.1.411	O	2021-02-09	Colombo	Female	47	NS3_T151S, NSP12_D445G, Spike_T676A, NSP12_M666I, NS8_Q18stop, Spike_H1159Y, N_T205I, NSP2_T166I, NS3_Q57H, NSP12_P323L, Spike_D614G, M_S4F, NSP6_L37F
CA94371	EPI_ISL_1233124	E	B.1.411	O	2021-02-09	Colombo	Female	27	NS3_R30H, NSP12_D445G, NSP6_C221F, NSP12_M666I, Spike_H1159Y, N_T205I, NSP2_T166I, NSP12_P323L, Spike_D614G, NSP6_L37F

CA94441	EPI_ISL_1233125	E	B.1.411	O	2021-02-09	Gampaha	Female	25	NSP12_D445G, NSP12_M666I, N_T205I, NSP2_T166I, Spike_V213L, NSP3_S1675I, Spike_V367F, NSP12_P323L, Spike_D614G, NSP6_L37F
CMC82058	EPI_ISL_1233088	E	B.1.411	G	2021-02-12	Colombo	Male	26	NSP12_D445G, N_K373R, NSP12_M666I, Spike_T716I, NS8_Q18stop, Spike_H1159Y, N_T205I, NSP2_T166I, NSP2_T85I, NSP12_P323L, Spike_D614G, NSP6_L37F
CMC82201	EPI_ISL_1239367	E	B.1.411	GH	2021-02-13	Colombo	Male	34	NSP12_M666I, NS8_Q18stop, Spike_H1159Y, NS8_T26I, N_T205I, NSP2_T166I, NS3_Q57H, NSP2_T85I, NS3_V202L, NSP13_V266L, NSP12_P323L, Spike_D614G, NSP6_L37F
CMC82662	EPI_ISL_1233092	E	B.1.411	GH	2021-02-13	Colombo	Female	57	NS3_Y107C, NSP12_D445G, NSP15_S261L, NSP12_M666I, NS8_Q18stop, Spike_H1159Y, N_T205I, NSP2_T166I, NS3_Q57H, NSP2_T85I, NSP12_P323L, Spike_D614G, M_A2S, NSP6_L37F, NSP3_P654S
CMC82701	EPI_ISL_1233093	E	B.1.411	GH	2021-02-13	Colombo	Female	48	NS3_R30H, N_T265I, NS3_A23S, NSP12_D445G, NSP6_C221F, NSP12_M666I, NS8_Q18stop, NSP10_V7L, Spike_H1159Y, N_T205I, NSP2_T166I, NS3_Q57H, NSP2_T85I, NSP12_P323L, Spike_D614G, NSP6_L37F
CMC82283	EPI_ISL_1233089	E	B.1.411	G	2021-02-14	Colombo	Male	34	NSP12_D445G, NSP12_M666I, NS8_Q18stop, Spike_H1159Y, N_T205I, NSP2_T166I, NSP2_T85I, NSP12_P323L, Spike_D614G, NSP6_L37F
CMC82395	EPI_ISL_1233090	E	B.1.411	G	2021-02-15	Gampaha	Male	39	NSP12_D445G, NSP12_M666I, NS8_Q18stop, Spike_H1159Y, N_T205I, NSP2_T166I, NSP2_T85I, NS3_D199Y, NSP12_P323L, Spike_D614G, NSP6_L37F
CMC82819	EPI_ISL_1233094	E	B.1.411	GH	2021-02-15	Colombo	Male	32	NSP14_K349N, NSP12_D445G, NSP12_M666I, NS8_Q18stop, Spike_H1159Y, NSP16_R86K, N_T205I, NSP2_T166I, NS3_Q57H, NSP2_T85I, NSP12_P323L, Spike_D614G, NSP6_L37F
CMC82904	EPI_ISL_1233129	E	B.1.411	G	2021-02-15	Colombo	Male	40	NSP12_D445G, NSP12_M666I, Spike_H1159Y, N_T205I, NSP2_T166I, NSP12_P323L, Spike_D614G, NSP6_L37F
CMC83045	EPI_ISL_1233095	E	B.1.411	G	2021-02-15	Colombo	Male	25	NSP12_D445G, NSP12_M666I, NS8_Q18stop, Spike_H1159Y, N_T205I, NSP2_T166I, NS3_V13L, NSP12_P323L, Spike_D614G
CMC83064	EPI_ISL_1233096	E	B.1.411	O	2021-02-15	Colombo	Male	25	NSP12_D445G, NS8_F120V, NSP12_M666I, NS8_I121L, NS8_Q18stop, NSP9_V76A, Spike_H1159Y, N_T205I, NSP2_T166I, NSP3_V267F, NS3_Q57H, Spike_D614G, NSP6_L37F, NSP2_H208Y
CMC83303	EPI_ISL_1239361	E	B.1.411	GH	2021-02-15	Colombo	Male	28	NSP12_M666I, NS8_Q18stop, Spike_H1159Y, N_T205I, NSP2_T166I, NS3_Q57H, NSP2_T85I, NS3_L108F, NS8_R52I, NSP12_P323L, Spike_D614G, NSP6_L37F, Spike_S640F
CMC83312	EPI_ISL_1233097	E	B.1.411	GH	2021-02-15	Colombo	Male	52	NSP12_D445G, NSP12_M666I, NS8_Q18stop, Spike_H1159Y, N_T205I, NSP2_T166I, NS3_Q57H, NSP2_T85I, NSP12_P323L, Spike_D614G, NSP6_L37F, Spike_E748D

RTH6	EPI_ISL_1233106	E	B.1.411	O	2021-02-15	Ratnapura	Female	17	NSP12_D445G, NSP2_T388I, NSP12_M666I, NS8_Q18stop, Spike_H1159Y, N_T205I, NSP2_T85I, NSP15_T48I, NSP12_P323L, NS3_Q185H, Spike_D614G, NSP6_L37F, NS3_S171L
RTH7	EPI_ISL_1233136	E	B.1.411	G	2021-02-15	Ratnapura	Female	48	NSP13_P326L, Spike_N751K, Spike_H1101Y, NSP12_D445G, NSP12_M666I, N_T205I, NSP3_L862F, NSP12_P323L, Spike_D614G, NSP6_L37F
RTH8	EPI_ISL_1233137	E	B.1.411	O	2021-02-15	Ratnapura	Male	54	NSP3_I967T, NSP12_D445G, NSP2_T388I, NSP12_M666I, Spike_H1159Y, NSP2_T166I, NS3_Q57H, NSP16_D26H, NSP3_A1280V, NSP15_T48I, NSP12_P323L, Spike_D614G, NSP6_L37F, NS3_S171L
CMC83757	EPI_ISL_1233098	E	B.1.258	GH	2021-02-16	Colombo	Female	57	NSP12_D445G, NSP12_M666I, Spike_T240I, NS8_Q18stop, Spike_H1159Y, N_T205I, NSP2_T166I, NS3_Q57H, NSP14_S503L, NSP12_P323L, Spike_D614G, NSP6_L37F
CA99139	EPI_ISL_1233126	E	B.1.411	G	2021-02-16	Colombo	Male	31	NSP12_D445G, NSP12_M666I, Spike_E484K, Spike_H1159Y, N_T205I, NSP2_T166I, NSP2_T85I, NS3_V202L, NSP12_P323L, Spike_D614G, NSP6_L37F
CA99145	EPI_ISL_1233080	E	B.1.411	GH	2021-02-16	Colombo	Female	20	NSP12_D445G, NSP12_M666I, NS8_Q18stop, Spike_H1159Y, N_T205I, NSP2_T166I, NS3_Q57H, NSP2_T85I, NSP12_P323L, Spike_A684V, Spike_D614G, NSP6_L37F, NSP3_P654S
CA99150	EPI_ISL_1233081	E	B.1.411	GH	2021-02-16	Colombo	Female	23	NSP12_D445G, NSP12_M666I, NS8_Q18stop, Spike_H1159Y, N_T205I, NSP2_T166I, NS3_Q57H, NSP2_T85I, NSP12_P323L, Spike_A684V, Spike_D614G, NSP6_L37F, NSP3_P654S
CMC82521	EPI_ISL_1233091	E	B.1.411	G	2021-02-16	Colombo	Male	51	NSP2_T170I, NSP3_L72F, NSP12_D445G, NSP12_M666I, NS8_Q18stop, Spike_H1159Y, NSP2_T166I, NSP12_D135Y, NSP6_F35L, NSP12_P323L, Spike_D614G, NSP6_L37F
CMC83433	EPI_ISL_1239364	E	B.1.411	GH	2021-02-16	Colombo	Male	70	NSP12_D445G, NSP12_M666I, NS8_Q18stop, Spike_H1159Y, N_T205I, NSP2_T166I, NS3_Q57H, NSP2_T85I, NS3_L108F, NS8_R52I, NSP12_P323L, Spike_D614G, NSP3_K1804N, NSP6_L37F, Spike_S640F
CMC83434	EPI_ISL_1239365	E	B.1.411	GH	2021-02-16	Colombo	Female	40	NSP12_D445G, NSP12_M666I, NS8_Q18stop, Spike_H1159Y, N_T205I, NSP2_T166I, NS3_Q57H, NS3_L108F, NS8_R52I, NSP12_P323L, Spike_D614G, NSP3_K1804N, NSP6_L37F, Spike_S640F
CMC83435	EPI_ISL_1239363	E	B.1.411	GH	2021-02-16	Colombo	Female	80	NSP12_N9Y, NSP12_D445G, NSP12_M666I, Spike_H1159Y, N_T205I, NSP2_T166I, NS3_Q57H, NSP2_T85I, NS3_L108F, NSP12_ins9stop, NS8_R52I, NSP12_R10C, NSP12_P323L, Spike_D614G, NSP3_K1804N, NSP6_L37F, NSP12_L8C, Spike_S640F
CMC83466	EPI_ISL_1239366	E	B.1.411	GH	2021-02-16	Colombo	Male	68	NSP12_M666I, NS8_Q18stop, Spike_H1159Y, N_R209K, N_T205I, NSP2_T166I, NS3_Q57H, NSP2_T85I, NS3_L108F, NS8_R52I, NSP12_P323L, Spike_D614G, NSP3_K1804N, NSP6_L37F

CMC83690	EPI_ISL_1233113	E	B.1.411	GH	2021-02-16	Colombo	Female	40	NSP12_M666I, NS8_Q18stop, NSP13_I333V, Spike_H1159Y, N_T205I, NSP2_T166I, NS3_Q57H, NSP2_T85I, NSP12_P323L, Spike_D614G, NSP6_L37F, Spike_D178G
CMC83755	EPI_ISL_1233055	E	B.1.411	GH	2021-02-16	Colombo	Male	57	NSP12_M666I, NS8_Q18stop, Spike_H1159Y, N_T205I, NSP2_T166I, NS3_Q57H, N_D402Y, Spike_V622F, NSP2_T85I, NSP12_P323L, Spike_D614G, NSP6_L37F
RTH2	EPI_ISL_1239368	E	B.1	G	2021-02-17	Ratnapura	Female	17	Spike_L18F, NSP12_D445G, NSP12_M666I, NS8_Q18stop, Spike_H1159Y, N_T205I, NSP14_P203L, NSP2_T85I, NSP12_P323L, Spike_D614G, NSP6_L37F
CMC83980	EPI_ISL_1233130	E	B.1.411	GH	2021-02-17	Colombo	Male	33	NSP12_D445G, NSP8_T148I, NSP12_M666I, Spike_H1159Y, N_T205I, NSP2_T166I, NS3_Q57H, NSP2_T85I, NSP12_P323L, Spike_D614G, NSP6_L37F, NSP3_G17C
CMC83982	EPI_ISL_1233099	E	B.1.411	GH	2021-02-17	Colombo	Male	32	NSP12_D445G, NSP3_K529R, NSP8_T148I, NSP12_M666I, NS8_Q18stop, Spike_H1159Y, NSP2_T166I, NS3_Q57H, NSP2_T85I, NSP12_P323L, Spike_D614G, NS8_I58V, NSP6_L37F, NSP3_G17C
CMC83991	EPI_ISL_1233100	E	B.1.411	GH	2021-02-17	Colombo	Male	41	NSP12_D445G, NSP3_K529R, NSP8_T148I, NSP12_M666I, NS8_Q18stop, Spike_H1159Y, N_T205I, NSP2_T166I, NS3_Q57H, NSP2_T85I, NSP12_P323L, Spike_D614G, NS8_I58V, NSP6_L37F
CMC84013	EPI_ISL_1233056	E	B.1.411	GH	2021-02-17	Colombo	Male	28	NS7b_L32F, NSP12_M666I, NS8_Q18stop, Spike_H1159Y, N_T205I, NSP2_T166I, NS3_Q57H, NSP2_T85I, NSP12_P323L, Spike_D614G, NSP6_L37F
CMC84237	EPI_ISL_1233058	E	B.1.411	O	2021-02-17	Colombo	Male	31	NSP12_M666I, NS8_Q18stop, Spike_H1159Y, N_T205I, NSP2_T166I, NS3_Q57H, NSP2_T85I, NSP12_P323L, Spike_D614G, NSP6_L37F, NS8_P93L
RTH1	EPI_ISL_1239362	E	B.1.411	G	2021-02-17	Ratnapura	Female	33	NSP3_T1303I, NSP12_M666I, NS8_Q18stop, Spike_T859I, N_T205I, NSP2_T166I, NSP12_P323L, Spike_D614G, NSP6_L37F, NSP3_P395L, NSP8_A21V
RTH4	EPI_ISL_1233105	E	B.1.411	G	2021-02-17	Ratnapura	Female	13	Spike_L18F, NSP12_D445G, NSP12_M666I, NS8_Q18stop, Spike_H1159Y, N_T205I, NSP2_T166I, NSP14_P203L, NSP2_T85I, NSP12_P323L, Spike_D614G, NSP6_L37F, NSP3_H1880Y
RTH3	EPI_ISL_1233135	E	B.1.411	O	2021-02-18	Ratnapura	Male	36	NSP12_D445G, NSP12_M666I, Spike_H1159Y, NSP2_T166I, NSP3_A667T, NSP3_H290Y, NSP12_P323L, Spike_D614G
DMC_C_111	EPI_ISL_1233061	E	B.1.411	O	2021-02-19	Matale	Female	17	NSP12_D445G, NSP3_A1321V, NSP12_M666I, NS8_Q18stop, Spike_H1159Y, N_T205I, NSP2_T166I, NS3_Q57H, NSP2_T85I, NSP12_P323L, Spike_D614G, NSP6_L37F
RATT_C_02	EPI_ISL_1233104	E	B.1.411	GH	2021-02-19	Matale	Male	18	NSP3_A1105T, NSP12_D445G, NSP3_P1228S, NSP12_M666I, NS8_Q18stop, Spike_H1159Y, N_T205I, NSP2_T166I, NS3_Q57H, NSP2_T85I, NSP3_Q995H, NSP12_P323L, NS3_S92L, Spike_D614G, NSP6_L37F

RTH5	EPI_ISL_1233117	E	B.1.411	GH	2021-02-19	Ratnapura	Female	48	NSP12_D445G, E_L39del, NSP12_M666I, Spike_H1159Y, N_T205I, NSP2_T166I, NS3_Q57H, E_C40del, NSP3_A667T, N_S201N, NSP3_H290Y, NSP12_P323L, E_A41S, Spike_D614G, NSP6_L37F
CN102751	EPI_ISL_1233060	E	B.1.411	GH	2021-02-22	Anuradhapura	Male	36	NS3_T151S, NSP12_D445G, Spike_T676A, NSP12_M666I, NS8_Q18stop, NSP1_V84del, Spike_H1159Y, NSP1_M85V, N_T205I, NSP2_T166I, NS3_Q57H, NSP2_T85I, NSP1_H83del, NSP12_P323L, Spike_D614G, NSP6_L37F, NSP1_G82del, NSP16_P236L
CW103180	EPI_ISL_1233111	E	B.1	GH	2021-02-23	Kalutara	Female	75	NSP7_T81I, NSP12_D445G, NS3_L41F, NSP12_M666I, NSP16_Q3L, Spike_H1159Y, N_T205I, NSP2_T166I, NS3_Q57H, NS8_E64stop, NSP2_G392R, NSP12_T85I, Spike_D614G, NSP2_K337E, NSP6_L37F
CW103177	EPI_ISL_1233114	E	B.1.411	GH	2021-02-23	Kalutara	Male	42	NSP7_T81I, NSP12_M666I, NS8_Q18stop, NSP16_Q3L, Spike_H1159Y, N_T205I, NSP2_T166I, NS3_Q57H, NSP2_T85I, NSP2_G392R, NSP12_T85I, NSP14_S450I, Spike_D614G, NSP2_K337E, NSP6_L37F
CW103178	EPI_ISL_1233131	E	B.1.411	G	2021-02-23	Kalutara	Male	73	NSP7_T81I, NSP12_D445G, NSP12_M666I, NSP16_Q3L, Spike_H1159Y, N_T205I, NSP2_T166I, NSP2_T85I, NSP2_G392R, NSP12_P323L, NSP12_T85I, NSP14_S450I, Spike_D614G, NSP2_K337E, NSP6_L37F
CW103259	EPI_ISL_1233132	E	B.1	GH	2021-02-24	Kalutara	Male	48	NSP7_T81I, NSP12_D445G, NS3_L41F, NSP16_Q3L, Spike_H1159Y, NSP2_T166I, NS3_Q57H, NSP2_G392R, NSP12_P323L, NSP12_T85I, NSP14_S450I, Spike_D614G, NSP2_K337E, NSP6_L37F
CA103286	EPI_ISL_1233066	E	B.1.411	GH	2021-02-24	Gampaha	Male	35	NSP12_D445G, NSP12_M666I, NS8_Q18stop, Spike_H1159Y, N_T205I, NSP2_T166I, NS3_Q57H, NSP14_S503L, NSP12_D284N, NSP12_P323L, Spike_D614G, NSP6_L37F
CA103306	EPI_ISL_1233067	E	B.1.411	G	2021-02-24	Gampaha	Female	37	NSP12_D445G, NSP12_M666I, NS8_Q18stop, Spike_H1159Y, N_T205I, NSP2_T166I, NSP2_T85I, NSP12_P323L, Spike_A684V, Spike_D614G, NSP6_L37F, NSP3_P654S
CW103261	EPI_ISL_1233133	E	B.1.411	GH	2021-02-24	Kalutara	Male	10	NSP7_T81I, NSP12_D445G, NS3_L41F, NSP12_M666I, NSP16_Q3L, Spike_H1159Y, N_T205I, NSP2_T166I, NS3_Q57H, NSP2_G392R, NSP12_P323L, NSP12_T85I, NSP14_S450I, Spike_D614G, NSP2_K337E, NSP6_L37F
CW103264	EPI_ISL_1233101	E	B.1.411	G	2021-02-24	Kalutara	Female	96	NSP7_T81I, NSP12_D445G, NSP12_M666I, NS8_Q18stop, NSP16_Q3L, Spike_H1159Y, N_T205I, NSP2_T166I, NSP2_G392R, NSP12_P323L, NSP12_T85I, NSP14_S450I, Spike_D614G, NSP2_K337E, NSP6_L37F
CW103267	EPI_ISL_1233102	E	B.1.411	GH	2021-02-24	Kalutara	Male	53	Spike_E180G, NSP12_D445G, NSP12_M666I, NS8_Q18stop, Spike_H1159Y, N_T205I, NSP2_T166I, NS3_Q57H, NSP2_T85I, Spike_D614G, NSP6_L37F

STF103572	EPI_ISL_1233138	E	B.1.411	GH	2021-02-24	Kalutara	Female	n/a	Spike_F1121L, NSP12_D445G, NSP12_M666I, Spike_H1159Y, N_T205I, NSP2_T166I, NS3_Q57H, NS8_V62L, NSP12_P323L, Spike_D614G, NSP6_L37F
CA103847	EPI_ISL_1233068	E	B.1.411	G	2021-02-25	Gampaha	Male	41	NSP12_D445G, NSP12_M666I, NS8_Q18stop, Spike_H1159Y, N_T205I, NSP2_T166I, NS3_D210Y, NSP12_P323L, Spike_D614G, NSP6_L37F
CA103848	EPI_ISL_1233069	E	B.1.411	GH	2021-02-25	Gampaha	Male	34	NS3_A59T, NSP12_D445G, NSP12_M666I, NS8_Q18stop, Spike_H1159Y, N_T205I, NS3_Q57H, NSP2_T85I, NSP12_P323L, Spike_D614G, NSP6_L37F
CA103909	EPI_ISL_1233070	E	B.1.411	G	2021-02-25	Colombo	Male	56	N_K387R, NSP12_D445G, Spike_M1237I, NSP12_M666I, NS8_Q18stop, N_T205I, NSP2_T166I, NSP2_T85I, Spike_A879T, NSP12_P323L, Spike_D614G, NSP6_L37F
CK104003	EPI_ISL_1233127	E	B.1.411	GH	2021-02-25	Colombo	Male	21	NSP12_D445G, NSP12_M666I, Spike_H1159Y, N_T205I, NSP2_T166I, NS3_Q57H, NSP2_T85I, NSP12_P323L, Spike_D614G, NSP6_L37F
CQ104596	EPI_ISL_1233063	E	B.1.1.7	GRY	2021-02-26	Quarantine	Male	25	Spike_H69del, NS8_Q27stop, NSP3_T183I, NSP1_V106A, NSP6_S106del, N_R203K, Spike_A570D, NSP2_L400F, Spike_N501Y, NSP3_I1412T, Spike_Y144del, NSP15_S288F, NSP3_A890D, NSP6_G107del, E_ins38CLL, Spike_D1118H, NSP6_F108del, NS8_Y73C, N_G204R, Spike_V70del, NS3_E261G, NSP12_P323L, Spike_D614G, N_D3L, N_S235F
SEQ01	EPI_ISL_1233107	E	B.1.411	G	2021-02-27	Colombo	Male	31	NSP12_D445G, NSP12_M666I, NS8_Q18stop, NSP2_K456R, Spike_H1159Y, N_D144Y, NSP2_T166I, NSP12_P323L, Spike_D614G, NSP6_L37F, NS8_P93L
NR128	EPI_ISL_1233134	E	B.1.1.25	GR	2021-03-02	Quarantine	Male	56	Spike_V1264L, NS6_I60V, N_R203K, NSP3_K412N, N_G204R, M_F28L, Spike_P681R, NS3_K75N, NS8_V62L, NSP2_I120F, NS8_S54L, NSP12_P323L, Spike_D614G, NSP6_L37F, Spike_Q677H, Spike_W258L
NR123	EPI_ISL_1233109	E	B.1.1.7	GRY	2021-03-02	Quarantine	Male	28	Spike_H69del, NS8_Q27stop, NSP3_T183I, Spike_T716I, NS8_K68stop, NSP6_S106del, N_R203K, Spike_A570D, NS3_W131C, Spike_E96D, Spike_G1251V, Spike_N501Y, NSP3_I1412T, NS8_R52I, Spike_P681H, Spike_Y144del, NSP6_G107del, Spike_D1118H, NSP6_F108del, N_G204R, Spike_V70del, NSP12_P323L, Spike_D614G, N_D3L, Spike_S982A
NR126	EPI_ISL_1233110	E	B.1.1.7	GRY	2021-03-02	Quarantine	Male	40	Spike_H69del, NS8_Q27stop, NSP3_T183I, Spike_T716I, NS8_K68stop, NSP6_S106del, N_R203K, Spike_A570D, NS3_T89I, Spike_N501Y, NSP3_I1412T, NS8_R52I, Spike_P681H, Spike_Y144del, NSP12_P227L, NSP3_P67L, NSP6_G107del, NSP3_A890D, NSP3_D782N, Spike_D1118H, NSP6_F108del, NS8_Y73C, N_G204R, Spike_V70del, NSP12_P323L, NSP14_P451S, Spike_D614G, N_D3L, Spike_S982A

NR127	EPI_ISL_1233119	E	B.1.351	G	2021-03-02	Quarantine	Male	30	Spike_D215G, E_P71L, NSP3_K837N, Spike_K417N, Spike_L244del, NSP6_G107del, NSP6_S106del, Spike_E484K, N_T205I, NSP6_F108del, Spike_L242del, Spike_A701V, NSP2_T85I, Spike_D80A, Spike_N501Y, NSP12_P323L, NSP4_L264F, NSP5_K90R, Spike_D614G, Spike_A243del, NS3_S171L
NR124	EPI_ISL_1233103	E	B.1.411	GH	2021-03-02	Quarantine	Male	40	NSP7_T81I, NSP12_D445G, NS3_L41F, NSP12_M666I, NS8_Q18stop, Spike_H1159Y, N_T205I, NSP2_T166I, NS3_Q57H, NSP12_P323L, NSP12_T85I, NSP14_S450I, Spike_D614G, NSP6_L37F, NSP3_N922S
NR22	EPI_ISL_1533846	E	B.1.1.7	GRY	2021-03-07	Qurantine	Female	71	Spike_H69del, NS8_Q27stop, NSP3_T183I, Spike_T716I, NSP6_S106del, N_R203K, Spike_A570D, Spike_N501Y, NS8_R52I, Spike_P681H, Spike_Y144del, NSP3_I441L, NSP6_G107del, NSP3_A890D, Spike_D1118H, NSP6_F108del, NS8_Y73C, N_G204R, Spike_V70del, NSP12_P323L, Spike_D614G, N_D3L, Spike_S982A, N_S235F
CDRF36	EPI_ISL_1533804	E	B.1.1.7	G	2021-03-09	Qurantine	Male	n/a	Spike_H69del, NSP3_T183I, NSP6_G107del, Spike_T716I, NSP6_S106del, N_R203K, Spike_D1118H, NSP6_F108del, NS3_Q57H, N_G204R, Spike_V70del, NSP3_I1412T, NS8_R52I, NSP3_T1830I, Spike_P681H, Spike_D614G, N_D3L, Spike_S982A, N_S235F
CDRI1	EPI_ISL_1533805	E	B.1.411	O	2021-03-09	Colombo	Male	48	NSP12_D445G, NSP12_M666I, NS8_Q18stop, Spike_H1159Y, N_T205I, NSP2_T166I, NSP3_L1328F, NSP2_T85I, NSP12_P323L, Spike_D614G, NSP6_L37F, NSP6_V149F, NSP2_E201A
CDRC2	EPI_ISL_1533803	E	B.1	G	2021-03-10	Colombo	Male	5	NSP13_S80G, NSP12_M666I, NS8_Q18stop, Spike_H1159Y, N_T205I, NS3_L106R, NSP2_T85I, NSP12_P323L, Spike_D614G, N_D377A, Spike_D178G
CDRS25	EPI_ISL_1533810	E	B.1	G	2021-03-10	Gampaha	Male	53	NSP12_D445G, NSP12_M666I, N_T205I, NSP2_T166I, NSP2_T85I, NSP12_P323L, Spike_A684V, Spike_D614G, NSP6_L37F, Spike_M1229I, NSP3_P654S, NSP13_A18V
CDRQ4	EPI_ISL_1533806	E	B.1.1.7	O	2021-03-10	Qurantine	Male	28	NS8_Q27stop, NSP3_T183I, NSP1_V106A, Spike_T716I, NSP6_S106del, Spike_A570D, NSP2_L400F, Spike_N501Y, NSP3_I1412T, NS8_R52I, Spike_P681H, Spike_Y144del, NSP15_S288F, NSP6_G107del, NSP3_A890D, Spike_D1118H, NSP6_F108del, NS8_Y73C, NS3_E261G, NSP12_P323L, Spike_D614G, N_D3L, Spike_S982A
CDRS22	EPI_ISL_1533807	E	B.1.411	O	2021-03-10	Gampaha	Male	45	NSP12_D445G, NSP12_M666I, Spike_H1159Y, N_T205I, NSP2_T166I, NS3_Q57H, NSP12_P323L, Spike_A684V, Spike_D614G, NSP6_L37F, Spike_M1229I, NSP3_P654S

CDRS23	EPI_ISL_1533808	E	B.1.411	O	2021-03-10	Gampaha	Male	54	NSP12_D445G, NSP12_M666I, NS8_Q18stop, Spike_H1159Y, N_T205I, NSP2_T166I, NS3_Q57H, NSP2_T85I, NSP12_P323L, Spike_A684V, Spike_D614G, NSP1_E36K, NSP6_L37F, Spike_M1229I, NSP3_P654S
CDRS24	EPI_ISL_1533809	E	B.1.411	GH	2021-03-10	Gampaha	Male	53	NSP12_D445G, NSP12_M666I, NS8_Q18stop, Spike_H1159Y, NSP2_T166I, NS3_Q57H, NSP2_T85I, Spike_A684V, Spike_D614G, NSP6_L37F, Spike_M1229I, NSP3_P654S
CDRS26	EPI_ISL_1533811	E	B.1.411	G	2021-03-10	Gampaha	Male	56	NSP12_M666I, Spike_H1159Y, N_T205I, NSP2_T166I, NSP2_T85I, NSP12_P323L, Spike_A684V, Spike_D614G, NSP6_L37F, NSP3_P654S
CDRS27	EPI_ISL_1533812	E	B.1.411	GH	2021-03-10	Gampaha	Male	28	NSP12_D445G, NSP12_M666I, NS8_Q18stop, Spike_H1159Y, N_T205I, NSP2_T166I, NS3_Q57H, NSP2_T85I, NSP12_P323L, Spike_A684V, Spike_D614G, NSP6_L37F, Spike_M1229I, NSP3_P654S
CDRS28	EPI_ISL_1533813	E	B.1.411	GH	2021-03-10	Gampaha	Male	44	NSP12_D445G, NSP12_M666I, NS8_Q18stop, Spike_H1159Y, N_T205I, NSP2_T166I, NS3_Q57H, NSP2_T85I, NSP12_P323L, Spike_A684V, Spike_D614G, NSP6_L37F, Spike_M1229I, NSP3_P654S
CDRS29	EPI_ISL_1533814	E	B.1.411	GH	2021-03-10	Gampaha	Male	53	NSP12_D445G, NSP12_M666I, NS8_Q18stop, Spike_H1159Y, N_T205I, NSP2_T166I, NS3_Q57H, NSP2_T85I, NSP12_P323L, Spike_A684V, Spike_D614G, NSP6_L37F, Spike_M1229I, NSP3_P654S
CDRS30	EPI_ISL_1533815	E	B.1.411	GH	2021-03-10	Gampaha	Male	45	NSP12_D445G, NSP12_M666I, Spike_H1159Y, N_T205I, NSP2_T166I, NS3_Q57H, NSP2_T85I, NSP12_P323L, Spike_A684V, Spike_D614G, NSP6_L37F, Spike_M1229I, NSP3_P654S
CDRS31	EPI_ISL_1533816	E	B.1.411	GH	2021-03-10	Gampaha	Male	67	NSP12_D445G, NSP12_M666I, NS8_Q18stop, Spike_H1159Y, N_T205I, NSP2_T166I, NS3_Q57H, NSP2_T85I, NSP12_P323L, Spike_A684V, Spike_D614G, NSP6_L37F, Spike_M1229I, NSP3_P654S
CDRS33	EPI_ISL_1533817	E	B.1.411	G	2021-03-10	Gampaha	Male	27	NSP3_D174Y, NSP12_M666I, NS8_Q18stop, Spike_H1159Y, N_T205I, NSP2_T166I, NSP2_T85I, NSP12_P323L, Spike_A684V, Spike_D614G, NSP6_L37F, Spike_M1229I, NSP3_P654S
CMC86624	EPI_ISL_1533822	E	B.1.411	G	2021-03-10	Colombo	Male	46	NSP12_D445G, NSP12_M666I, Spike_H1159Y, N_T205I, NSP2_T166I, NS3_P262S, NSP2_T85I, NSP12_P323L, Spike_D614G, NSP6_L37F

NR23	EPI_ISL_1533847	E	B.1.1.7	O	2021-03-12	Qurantine	Male	14	Spike_H69del, Spike_T716I, NS8_K68stop, NSP6_S106del, N_R203K, Spike_A570D, NS3_T89I, NSP5_L75F, Spike_N501Y, NSP3_I1412T, NS8_R52I, Spike_P681H, Spike_Y144del, NSP12_P227L, NSP3_P67L, NSP6_G107del, NSP3_A890D, NSP3_D782N, Spike_D1118H, NSP6_F108del, NS8_Y73C, N_G204R, Spike_V70del, NSP12_P323L, NSP14_P451S, Spike_D614G, N_D3L
NR24	EPI_ISL_1533848	E	B.1.1.7	O	2021-03-12	Qurantine	Female	40	NS8_Q27stop, NSP3_T183I, NS8_K68stop, NSP6_S106del, N_R203K, Spike_A570D, NS3_T89I, NSP5_L75F, Spike_N501Y, NS8_R52I, Spike_P681H, Spike_Y144del, NSP12_P227L, NSP3_P67L, NSP6_G107del, NSP3_A890D, NSP3_D782N, Spike_D1118H, NSP6_F108del, NS8_Y73C, N_G204R, NSP14_P451S, Spike_D614G, N_D3L, Spike_S982A
NR9	EPI_ISL_1533851	E	B.1.1.7	G	2021-03-12	Qurantine	Male	18	Spike_H69del, NS8_Q27stop, NSP3_T183I, Spike_T716I, NS8_K68stop, NSP6_S106del, Spike_A570D, Spike_N501Y, NSP3_I1412T, NS8_R52I, Spike_P681H, Spike_Y144del, NSP12_P227L, NSP3_P67L, NSP6_G107del, NSP3_A890D, NSP3_D782N, Spike_D1118H, NSP6_F108del, NS8_Y73C, Spike_V70del, NSP12_P323L, NSP14_P451S, Spike_D614G, N_D3L, Spike_S982A
CMC86789	EPI_ISL_1533823	E	B.1.411	O	2021-03-15	Colombo	Male	49	NSP12_D445G, NSP12_M666I, NS8_Q18stop, Spike_H1159Y, N_T205I, N_D144Y, NSP3_T204I, NSP15_T48I, Spike_D614G, NSP6_L37F, NS8_P93L
CMC86811	EPI_ISL_1533824	E	B.1.411	G	2021-03-15	Colombo	Male	52	NSP3_G777V, NSP3_G337S, NSP12_D445G, NSP12_D618N, NSP12_M666I, Spike_H1159Y, N_T205I, N_D144Y, NSP12_ins617LR, NSP2_T166I, NSP3_ins336DHNY, NSP3_A338H, NSP3_T204I, NSP2_T85I, NSP15_T48I, NSP12_P323L, Spike_D614G, NSP6_L37F, NS8_P93L, NSP12_W617F
CP107829	EPI_ISL_1533839	E	B.1.411	GH	2021-03-15	Colombo	Male	41	NSP12_D445G, NSP12_M666I, NS3_E102Q, NSP16_R86K, N_T205I, NSP2_T166I, NS3_Q57H, NSP5_K90R, Spike_D614G, NSP6_L37F, NSP2_G147D
CP107863	EPI_ISL_1533841	E	B.1.411	GH	2021-03-15	Colombo	Male	38	NSP3_S284C, NSP12_M666I, NS8_Q18stop, NS3_E102Q, Spike_H1159Y, NSP3_P2L, NSP16_R86K, N_T205I, NSP2_T166I, NS3_Q57H, NSP2_T85I, NSP5_K90R, Spike_D614G, NSP6_L37F, NSP2_G147D
CP107818	EPI_ISL_1533838	E	B.1.428	GH	2021-03-15	Colombo	Male	56	NSP12_M666I, NS8_Q18stop, NS3_E102Q, Spike_H1159Y, NSP3_P2L, NSP16_R86K, N_T205I, NSP2_T166I, NS3_Q57H, NSP2_T85I, NSP12_P323L, NSP5_K90R, Spike_D614G, NSP6_L37F, NSP2_G147D

CP107831	EPI_ISL_1533840	E	B.1.428	GH	2021-03-15	Colombo	Male	46	NSP3_S284C, NSP12_D445G, NSP12_M666I, NS8_Q18stop, NS3_E102Q, Spike_H1159Y, NSP3_P2L, NSP16_R86K, N_T205I, NSP2_T166I, NS3_Q57H, NSP2_T85I, NSP3_T864I, NSP12_P323L, NSP5_K90R, Spike_D614G, NSP6_L37F, NSP2_G147D
CP107951	EPI_ISL_1533842	E	B.1.428	GH	2021-03-15	Colombo	Male	53	NSP3_S284C, NSP12_D445G, NSP12_M666I, NS8_Q18stop, NS3_E102Q, Spike_H1159Y, NSP3_P2L, NSP16_R86K, N_T205I, NSP2_T166I, NS3_Q57H, NSP2_T85I, NSP12_P323L, NSP5_K90R, Spike_D614G, NSP6_L37F, NSP2_G147D
CMC87036	EPI_ISL_1533825	E	B.1.411	G	2021-03-16	Colombo	n/a	84	NS3_T151S, NSP12_D445G, Spike_T676A, NSP12_M666I, Spike_H1159Y, N_T205I, NSP2_T166I, NSP12_P323L, Spike_D614G, NSP6_L37F, NSP14_P140S
CMC87038	EPI_ISL_1533826	E	B.1.411	G	2021-03-16	Colombo	n/a	75	NS3_T151S, NSP12_D445G, Spike_T676A, NSP12_M666I, NS8_Q18stop, Spike_H1159Y, N_T205I, NSP2_T166I, NSP12_P323L, Spike_D614G, NSP6_L37F
CMC87040	EPI_ISL_1533827	E	B.1.411	G	2021-03-16	Colombo	n/a	68	NS3_T151S, NSP12_D445G, Spike_T676A, NSP12_M666I, NS8_Q18stop, Spike_H1159Y, NSP2_T166I, NSP12_P323L, Spike_D614G, NSP6_L37F
CMC87041	EPI_ISL_1533828	E	B.1.411	GH	2021-03-16	Colombo	n/a	74	NS3_T151S, NSP12_D445G, NSP12_M666I, Spike_H1159Y, NSP2_T166I, NS3_Q57H, NSP2_T85I, NSP12_P323L, Spike_D614G, NSP6_L37F
CMC87046	EPI_ISL_1533829	E	B.1.411	G	2021-03-16	Colombo	n/a	85	NS3_T151S, NSP12_D445G, NSP12_M666I, NS8_Q18stop, Spike_H1159Y, N_T205I, NSP2_T166I, NSP12_P323L, Spike_D614G, NSP6_L37F
CMC87068	EPI_ISL_1533830	E	B.1.411	O	2021-03-16	Colombo	Female	68	NS3_T151S, NSP12_D445G, Spike_T676A, NSP12_M666I, NS8_Q18stop, Spike_H1159Y, N_T205I, NSP2_T166I, NS3_Q57H, NSP12_P323L, Spike_D614G, NSP6_L37F
CMC87070	EPI_ISL_1533831	E	B.1.411	O	2021-03-16	Colombo	Female	56	NSP10_T12I, NS3_T151S, NSP12_D445G, NSP12_M666I, Spike_H1159Y, NSP2_T166I, NS3_Q57H, NSP12_P323L, Spike_D614G, NSP6_L37F
CM108341	EPI_ISL_1533821	E	B.1.411	O	2021-03-17	Mannar	Female	26	NS8_L95F, NS8_W45C, NSP12_D445G, NSP16_Q238H, NSP12_M666I, NS8_Q18stop, Spike_H1159Y, N_T205I, NSP2_T166I, NSP1_G30S, E_S55F, NSP3_S1670F, NS7a_L102P, NSP12_P323L, NSP5_K90R, Spike_D614G, NSP6_L37F
CMC87128	EPI_ISL_1533832	E	B.1.411	G	2021-03-17	Colombo	Female	70	NS3_T151S, NSP12_D445G, NSP12_M666I, NS8_Q18stop, Spike_H1159Y, N_T205I, NSP2_T166I, NSP12_P323L, Spike_D614G, NSP6_L37F
CMC87130	EPI_ISL_1533833	E	B.1.411	G	2021-03-17	Colombo	Female	70	NS3_T151S, NSP12_D445G, Spike_T676A, NSP12_M666I, NS8_Q18stop, Spike_H1159Y, N_T205I, NSP2_T166I, NSP12_P323L, Spike_D614G, NSP6_L37F
CMC87137	EPI_ISL_1582411	E	B.1.411	O	2021-03-17	Colombo	Female	78	NS3_T151S, NSP12_D445G, Spike_T676A, NSP12_M666I, NS8_Q18stop, Spike_H1159Y, N_T205I, NSP2_T166I, NSP2_T85I, NSP12_P323L, Spike_D614G

CMC87140	EPI_ISL_1533834	E	B.1.411	G	2021-03-17	Colombo	Female	83	NS3_T151S, NSP12_D445G, Spike_T676A, NSP12_M666I, Spike_H1159Y, N_T205I, NSP2_T166I, NSP12_P323L, Spike_D614G
CMC87144	EPI_ISL_1533835	E	B.1.411	O	2021-03-17	Colombo	Female	73	NS3_T151S, NSP12_D445G, Spike_T676A, NSP12_M666I, NS8_Q18stop, Spike_H1159Y, N_T205I, NSP2_T166I, NSP1_G137S, NSP12_P323L, Spike_D614G, NSP6_L37F
CMC87190	EPI_ISL_1533836	E	B.1.411	O	2021-03-17	Colombo	Male	86	NS3_T151S, NSP12_D445G, Spike_T676A, NSP12_M666I, Spike_H1159Y, N_T205I, NSP2_T166I, NS3_Q57H, Spike_D614G, NSP6_L37F
CMC87205	EPI_ISL_1533837	E	B.1.411	O	2021-03-17	Colombo	Male	74	NS3_T151S, NSP12_D445G, Spike_T676A, NSP12_M666I, Spike_H1159Y, N_T205I, NSP2_T166I, NS3_Q57H, NS3_V55I, NSP12_P323L, Spike_D614G, NSP6_L37F
NR18	EPI_ISL_1582414	E	B.1	O	2021-03-20	Qurantine	Male	25	Spike_D215G, E_P71L, NSP3_K837N, Spike_K417N, Spike_L244del, NSP6_G107del, NS8_I121L, NSP6_S106del, Spike_E484K, N_T205I, NSP6_F108del, Spike_L242del, NS3_Q57H, NSP12_T120E, Spike_D80A, NSP12_P323L, Spike_D614G, NSP13_T588I, Spike_A243del, NS3_S171L
NR27	EPI_ISL_1533850	E	B.1.351	GH	2021-03-20	Qurantine	Male	26	NSP3_T1251I, Spike_D215G, E_P71L, NSP3_K837N, Spike_K417N, Spike_L244del, NSP6_G107del, NS8_I121L, NSP6_S106del, Spike_E484K, N_T205I, NSP6_F108del, Spike_L242del, NS3_Q57H, Spike_A701V, Spike_D80A, Spike_N501Y, NSP12_P323L, NSP5_K90R, Spike_D614G, Spike_A243del, NS3_S171L
NR25	EPI_ISL_1533849	E	B.1.1.7	GRY	2021-03-21	Qurantine	Male	37	Spike_H69del, NS8_Q27stop, NSP3_T183I, Spike_T716I, NS8_K68stop, NSP6_S106del, N_R203K, Spike_A570D, Spike_N501Y, NSP3_I1412T, NS8_R52I, Spike_P681H, Spike_Y144del, NSP6_G107del, NSP3_A890D, Spike_D1118H, NSP6_F108del, NSP13_S38L, NS8_Y73C, N_G204R, Spike_V70del, NSP12_P323L, Spike_D614G, NSP6_L37F, N_D3L, Spike_S982A
CDRW10	EPI_ISL_1533818	E	B.1.411	O	2021-03-21	Kegalle	Male	71	NSP12_D445G, NSP12_M666I, NS8_Q18stop, Spike_H1159Y, N_T205I, NSP2_T166I, NS3_Q57H, NSP2_T85I, NSP12_P323L, Spike_D614G, NSP6_L37F
CDRW11	EPI_ISL_1533819	E	B.1.411	O	2021-03-21	Kegalle	Female	32	NSP12_D445G, NSP12_M666I, NS8_Q18stop, Spike_H1159Y, N_T205I, NSP2_T166I, NSP12_P323L, Spike_D614G, NSP6_L37F
CDRW12	EPI_ISL_1533820	E	B.1.411	G	2021-03-21	Kegalle	Female	70	NSP12_D445G, NSP12_M666I, Spike_H1159Y, NSP2_T166I, NSP12_P323L, Spike_D614G, NSP6_L37F
NR17	EPI_ISL_1582413	E	B.1.1.365	G	2021-03-22	Qurantine	Female	27	N_L139F, NSP3_T1189I, N_S194L, Spike_E484K, NSP3_A1165V, NSP12_D92G, NSP12_P323L, Spike_D614G, NS3_G100C

NR26	EPI_ISL_1582415	E	B.1.351	GH	2021-03-22	Qurantine	Male	39	NSP3_T1251I, E_P71L, NSP3_K837N, Spike_K417N, NS8_I121L, NSP6_S106del, Spike_E484K, Spike_A701V, NSP3_V1933P, NSP3_V1935R, NSP3_T1938N, Spike_A243del, Spike_L244del, NSP6_G107del, N_T205I, NSP6_F108del, Spike_L242del, NS3_Q57H, NSP3_K1939I, Spike_D80A, NSP12_P323L, NSP5_K90R, Spike_D614G, NSP13_T588I, NSP3_V1936K, NS3_S171L
CP108816	EPI_ISL_1533843	E	B.1.411	GH	2021-03-23	Colombo	Male	26	NSP12_D445G, NSP12_M666I, NS8_Q18stop, Spike_H1159Y, N_T205I, NSP2_T166I, NS3_Q57H, NSP2_T85I, Spike_D287N, NSP12_P323L, Spike_D614G, NSP6_L37F
CP108841	EPI_ISL_1533844	E	B.1.411	GH	2021-03-23	Colombo	Male	37	NSP12_M666I, NS8_Q18stop, Spike_H1159Y, N_T205I, NSP2_T166I, NS3_Q57H, NSP2_T85I, Spike_D287N, NSP5_K90R, Spike_D614G, NSP6_L37F
CP108864	EPI_ISL_1533845	E	B.1.411	G	2021-03-23	Colombo	Male	31	NSP12_D445G, NSP12_M666I, NS8_Q18stop, Spike_H1159Y, N_T205I, NSP2_T166I, Spike_D287N, NSP12_P323L, Spike_D614G, NSP6_L37F
CM109233	EPI_ISL_1582410	E	B.1.411	G	2021-03-24	Mannar	Female	18	NSP12_D445G, N_K373R, NSP12_M666I, Spike_H1159Y, N_T205I, NSP2_T166I, NSP12_P323L, Spike_D614G, NSP6_L37F
CMC87762	EPI_ISL_1582412	E	B.1	G	2021-03-25	Colombo	Male	42	NSP12_D445G, NS8_Q18stop, Spike_H1159Y, N_T205I, NSP2_T166I, NS3_L108F, NS8_R52I, NSP12_P323L, Spike_D614G, NSP6_L37F, Spike_S640F
NR28	EPI_ISL_1970416	E	B.1.351	GH	2021-03-26	Quarantine	Male	28	NSP3_T1251I, E_P71L, NSP3_K837N, Spike_K417N, NS8_I121L, NSP6_S106del, Spike_E484K, NSP13_P78S, Spike_N501Y, Spike_A243del, Spike_D215G, Spike_L244del, NSP6_G107del, N_T205I, NSP6_F108del, Spike_L242del, NS3_Q57H, NSP2_T85I, Spike_D80A, NSP12_P323L, NSP5_K90R, Spike_D614G, NSP13_T588I, NS3_S171L
CDR72	EPI_ISL_1970385	E	B.1.411	O	2021-03-26	Jaffna	Male	57	NSP12_M666I, Spike_H1159Y, NSP2_T166I, NSP1_G30S, NSP12_P323L, NSP5_K90R, Spike_D614G, NSP6_L37F
CDR71	EPI_ISL_1970384	E	B.1.428	GH	2021-03-26	Jaffna	Male	35	NS8_W45C, NSP12_D445G, NSP12_M666I, NS8_Q18stop, Spike_H1159Y, NS3_L53F, N_T205I, NSP2_T166I, NS3_Q57H, NSP1_G30S, NSP2_T85I, NSP3_S1670F, NSP12_P323L, NSP5_K90R, Spike_D614G, NSP6_L37F
NR29	EPI_ISL_1970417	E	B.1.1.7	O	2021-03-30	Quarantine	Male	49	NS8_Q27stop, NSP3_A890D, NSP6_G107del, Spike_T716I, NS8_K68stop, NSP6_S106del, N_R203K, Spike_A570D, Spike_D1118H, NS3_W131C, NSP6_F108del, NS8_Y73C, N_G204R, Spike_N501Y, NSP3_I1412T, NS8_R52I, NSP12_P323L, Spike_P681H, Spike_D614G, Spike_Y144del, N_D3L, Spike_S982A

CDR38	EPI_ISL_1970355	E	B.1.1.7	GR	2021-04-03	Colombo	Male	30	Spike_H69del, NS8_Q27stop, NSP3_T183I, Spike_T716I, NS8_K68stop, NSP6_S106del, N_R203K, Spike_A570D, NSP3_I1412T, NS8_R52I, Spike_P681H, Spike_Y144del, NSP6_G107del, NSP3_A890D, Spike_D1118H, NSP6_F108del, N_G204R, Spike_V70del, NS3_E194D, NSP12_P323L, Spike_D614G, N_D3L, Spike_S982A, N_S235F
CDR39	EPI_ISL_1970356	E	B.1.1.7	GR	2021-04-06	Boralesgamuwa	Male	27	NS8_Q27stop, NSP3_T183I, NSP8_T145I, Spike_T716I, NS8_K68stop, NSP6_S106del, N_R203K, Spike_A570D, Spike_N501Y, NSP3_I1412T, NS8_R52I, Spike_P681H, Spike_Y144del, NSP6_G107del, NSP3_A890D, Spike_D1118H, NSP6_F108del, NS8_Y73C, N_G204R, NS3_E194D, NSP12_P323L, Spike_D614G, N_D3L, Spike_S982A, N_S235F
CDR40	EPI_ISL_1970357	E	B.1.1.7	GRY	2021-04-06	Boralesgamuwa	Male	25	Spike_H69del, NS8_Q27stop, NSP3_T183I, NSP8_T145I, Spike_T716I, NS8_K68stop, NSP6_S106del, N_R203K, Spike_A570D, Spike_N501Y, NSP3_I1412T, NS8_R52I, Spike_P681H, Spike_Y144del, NSP6_G107del, NSP3_A890D, Spike_D1118H, NSP6_F108del, NS8_Y73C, N_G204R, Spike_V70del, NS3_E194D, NSP12_P323L, Spike_D614G, N_D3L, Spike_S982A
CDR41	EPI_ISL_1970358	E	B.1.1.7	GR	2021-04-06	Boralesgamuwa	Female	35	NS8_Q27stop, NSP3_T183I, NSP8_T145I, NS8_K68stop, NSP6_S106del, N_R203K, Spike_A570D, Spike_N501Y, NSP3_I1412T, NS8_R52I, Spike_P681H, Spike_Y144del, NSP6_G107del, NSP3_A890D, Spike_D1118H, NSP6_F108del, NS8_Y73C, N_G204R, NS3_E194D, NSP12_P323L, Spike_D614G, NSP2_S430L, N_D3L, Spike_S982A, N_S235F
CDR56	EPI_ISL_1970370	E	B.1.411	O	2021-04-07	Jaffna	Male	36	NS8_W45C, NSP3_K429N, NSP12_D445G, NSP3_G145C, NSP12_M666I, NS8_Q18stop, Spike_H1159Y, NS3_L53F, N_T205I, NSP2_T166I, NS3_Q57H, NSP2_T85I, NSP3_S1670F, NSP5_K90R, Spike_D614G, NSP6_L37F, NSP12_Y546C, Spike_G261V
CDR57	EPI_ISL_1970371	E	B.1.411	G	2021-04-07	Jaffna	Male	16	NS8_W45C, NSP3_K429N, NSP12_D445G, NSP12_M666I, Spike_H1159Y, NSP3_K945N, N_T205I, NSP2_T166I, NSP2_T85I, NSP3_S1670F, NSP12_P323L, NSP5_K90R, Spike_D614G, NSP6_L37F
CDR59	EPI_ISL_1970373	E	B.1.411	G	2021-04-07	Jaffna	Female	19	NS8_W45C, NSP3_K429N, NSP12_D445G, NSP12_M666I, NS8_Q18stop, Spike_H1159Y, N_T205I, NSP2_T166I, NSP2_T85I, NSP9_G37R, NSP3_S1670F, NSP12_P323L, NSP5_K90R, Spike_D614G, NSP6_L37F
CDR58	EPI_ISL_1970372	E	B.1.428	GH	2021-04-07	Jaffna	Male	82	NS8_W45C, NSP3_K429N, NSP12_D445G, NSP12_M666I, Spike_H1159Y, NS3_L53F, N_T205I, NSP2_T166I, NS3_Q57H, NSP3_S1670F, NSP12_P323L, NSP5_K90R, Spike_D614G, NSP6_L37F

NR44	EPI_ISL_1970418	E	B.1.617.2	G	2021-04-07	Quarantine	Male	32	NS7a_L116F, N_D63G, N_R203M, NSP12_G671S, Spike_G142D, NSP2_P129L, NS3_S26L, NSP14_P46L, NSP2_R246H, Spike_P681R, Spike_R158del, NS7a_V82A, Spike_F157del, Spike_T19R, NS7a_T120I, M_I82T, NSP6_V149A, Spike_D950N, NSP3_P822L, NSP13_P77L, Spike_E156G, NSP4_A446V, NSP12_P323L, Spike_D614G, Spike_L452R, NS7a_T11K
CDR42	EPI_ISL_1970359	F	B.1.1.7	O	2021-04-19	Colombo	Female	49	Spike_H69del, NS8_Q27stop, NSP3_T183I, Spike_T716I, NS8_K68stop, NSP6_S106del, N_R203K, NSP3_D110Y, Spike_A570D, NSP15_E260A, Spike_N501Y, NSP3_I1412T, NS8_R52I, NSP8_S76F, Spike_P681H, Spike_Y144del, NSP6_G107del, NSP3_A890D, Spike_D1118H, NSP6_F108del, N_G204R, Spike_V70del, NSP12_P323L, Spike_D614G, N_D3L, Spike_S982A
CMC88881	EPI_ISL_1972232	F	B.1.1.7	GR	2021-04-19	Colombo	Female	41	Spike_H69del, NS8_Q27stop, NSP3_T183I, Spike_T716I, NSP6_S106del, N_R203K, NSP3_D110Y, NSP15_E260A, NSP3_R1297del, NSP3_I1412T, NS8_R52I, NSP8_S76F, Spike_P681H, NSP3_V1298del, NSP6_G107del, NSP3_A890D, Spike_D1118H, NSP6_F108del, N_G204R, Spike_V70del, NSP3_S1296del, Spike_N149del, NSP12_P323L, Spike_D614G, N_D3L, Spike_S982A, N_S235F
CMC88939	EPI_ISL_1970412	F	B.1.1.7	GRY	2021-04-19	Colombo	Male	40	Spike_H69del, NS8_Q27stop, NSP3_T183I, NS8_K68stop, NSP6_S106del, N_R203K, NSP3_D110Y, Spike_A570D, NSP15_E260A, Spike_N501Y, NSP3_R1297del, NSP3_L689F, NSP3_I1412T, NSP8_S76F, Spike_P681H, Spike_Y144del, NS7b_S31L, NSP3_V1298del, NSP3_A890D, NSP6_G107del, Spike_D1118H, NSP6_F108del, NS8_Y73C, N_G204R, Spike_V70del, NSP3_S1296del, NSP12_P323L, Spike_D614G, N_D3L, Spike_S982A, N_S235F
CMC89508	EPI_ISL_1972230	F	B.1.1.7	GR	2021-04-20	Colombo	Male	38	NS8_Q27stop, NSP3_T183I, Spike_T716I, NS8_K68stop, Spike_N370Y, NSP6_S106del, N_R203K, NSP14_A320V, Spike_N501Y, NSP3_I1412T, NS8_R52I, Spike_P681H, Spike_Y144del, Spike_ins370KLVPFWstopSF, NSP6_G107del, NSP3_A890D, Spike_D1118H, NSP6_F108del, NS8_Y73C, NS3_E194D, NSP12_P323L, Spike_D614G, N_D3L, Spike_S982A
CDR104	EPI_ISL_1970352	F	B.1.1.7	GRY	2021-04-21	Kandy	Female	33	Spike_H69del, NS8_Q27stop, NSP3_T183I, NSP8_T145I, Spike_T716I, NS8_K68stop, NSP6_S106del, N_R203K, Spike_A570D, Spike_N501Y, NSP3_I1412T, NS8_R52I, Spike_P681H, Spike_Y144del, NSP6_G107del, NSP3_A890D, Spike_D1118H, NSP6_F108del, NS8_Y73C, N_G204R, Spike_V70del, NS3_E194D, NSP12_P323L, Spike_D614G, N_D3L, Spike_S982A, N_S235F

CDR105	EPI_ISL_1970353	F	B.1.1.7	GR	2021-04-21	Kandy	Female	33	Spike_H69del, NS8_Q27stop, NSP3_T183I, Spike_T716I, NS8_K68stop, NSP3_W1196C, NSP6_S106del, N_R203K, NSP15_E260A, NSP3_I1412T, NS8_R52I, NSP8_S76F, Spike_P681H, Spike_Y144del, NSP6_G107del, NSP3_A890D, Spike_D1118H, NSP6_F108del, NS8_Y73C, N_G204R, Spike_V70del, NSP12_P323L, Spike_D614G, N_D3L, Spike_S982A, N_S235F
CDR106	EPI_ISL_1970354	F	B.1.1.7	GRY	2021-04-21	Kandy	Male	49	Spike_H69del, NS8_Q27stop, NSP3_T183I, NSP8_T145I, Spike_T716I, NS8_K68stop, NSP6_S106del, N_R203K, Spike_A570D, Spike_N501Y, NSP3_I1412T, NS8_R52I, Spike_P681H, Spike_Y144del, NSP6_G107del, NSP3_A890D, Spike_D1118H, NSP6_F108del, NS8_Y73C, N_G204R, NSP15_H234Y, Spike_V70del, NS3_E194D, NSP12_P323L, Spike_D614G, N_D3L, Spike_S982A, N_S235F
CDR107	EPI_ISL_1972227	F	B.1.1.7	G	2021-04-21	Kandy	Male	65	Spike_H69del, NS8_Q27stop, NSP3_T183I, NSP12_L775M, NSP3_A890D, NS8_K68stop, Spike_D1118H, NSP12_Q773H, NS8_Y73C, Spike_V70del, NS3_E194D, NSP3_I1412T, NS8_R52I, NSP12_P323L, NSP12_ins772LstopA, Spike_P681H, Spike_D614G, Spike_Y144del, NSP12_ins774TLR, N_D3L, Spike_S982A
CDR43	EPI_ISL_1970360	F	B.1.1.7	G	2021-04-21	Gampaha	Male	35	Spike_H69del, NS8_Q27stop, NSP3_T183I, NSP3_W1196C, NSP6_S106del, NSP3_D110Y, Spike_A570D, NSP15_E260A, NSP3_R1297del, NSP3_I1412T, NSP8_S76F, Spike_P681H, Spike_Y144del, NSP3_V1298del, NSP3_A890D, NSP6_G107del, Spike_D1118H, NSP6_F108del, Spike_V70del, NSP3_S1296del, E_ins38CLX, NSP12_P323L, Spike_D614G, N_D3L, Spike_S982A
CDR44	EPI_ISL_1970361	F	B.1.1.7	O	2021-04-21	Colombo	Male	17	NS8_Q27stop, NSP3_T183I, Spike_T716I, NS8_K68stop, NSP6_S106del, N_R203K, NSP3_D110Y, NSP15_E260A, Spike_N501Y, NSP3_R1297del, NSP3_I1412T, NS8_R52I, NSP8_S76F, Spike_P681H, Spike_Y144del, NSP3_V1298del, NSP6_G107del, NSP3_A890D, Spike_D1118H, NSP6_F108del, NS8_Y73C, N_G204R, NSP3_S1296del, NSP12_P323L, Spike_D614G, N_D3L, Spike_S982A, N_S235F
CMC90016	EPI_ISL_1970413	F	B.1.1.7	G	2021-04-21	Colombo	Male	49	Spike_H69del, NS8_Q27stop, NSP3_T183I, Spike_T716I, NS8_K68stop, NSP6_S106del, Spike_A570D, NSP15_E260A, Spike_N501Y, NSP3_R1297del, NSP3_I1412T, NS8_R52I, NSP8_S76F, Spike_P681H, Spike_Y144del, NSP3_V1298del, NSP6_G107del, NSP3_A890D, Spike_D1118H, NSP6_F108del, NS8_Y73C, Spike_V70del, NSP3_S1296del, NSP12_P323L, Spike_D614G, N_D3L, Spike_S982A, N_S235F

CDR45	EPI_ISL_1970362	F	B.1.1.7	GRY	2021-04-22	Kurunegala	Female	57	Spike_H69del, NS8_Q27stop, NSP3_T183I, Spike_T716I, NS8_K68stop, NSP6_S106del, N_R203K, NSP3_D110Y, Spike_A570D, NSP15_E260A, Spike_N501Y, NSP3_I1412T, NS8_R52I, NSP8_S76F, Spike_P681H, Spike_Y144del, NSP6_G107del, NSP3_A890D, Spike_D1118H, NSP6_F108del, NS8_Y73C, N_G204R, Spike_V70del, NSP12_P323L, Spike_D614G, N_D3L, Spike_S982A, N_S235F
CDR46	EPI_ISL_1970363	F	B.1.1.7	GRY	2021-04-22	Kurunegala	Male	n/a	Spike_H69del, NSP3_T183I, Spike_T716I, NS8_K68stop, NSP6_S106del, N_R203K, NSP3_D110Y, Spike_A570D, NSP15_E260A, NSP3_M560I, Spike_N501Y, NSP3_I1412T, NS8_R52I, NSP8_S76F, Spike_P681H, Spike_Y144del, NSP6_G107del, NSP3_A890D, Spike_D1118H, NSP6_F108del, NS8_Y73C, N_G204R, Spike_V70del, NSP12_P323L, Spike_D614G, N_D3L, Spike_S982A, N_S235F
CDR47	EPI_ISL_1972233	F	B.1.1.7	GR	2021-04-22	Kurunegala	Female	16	Spike_H69del, NS8_Q27stop, NSP3_T183I, Spike_T716I, NSP12_Q5H, NSP3_W1196C, NSP6_S106del, N_R203K, NSP3_D110Y, Spike_A570D, NSP15_E260A, NSP12_F7L, NSP12_N9I, Spike_N501Y, NSP3_R1297del, NSP12_S6stop, NSP3_I1412T, NS8_R52I, NSP8_S76F, Spike_P681H, NSP12_V11M, NSP3_V1298del, NSP6_G107del, NSP3_A890D, Spike_D1118H, NSP6_F108del, NS8_Y73C, N_G204R, NSP12_R10A, Spike_V70del, NSP12_G13T, NSP3_S1296del, NSP12_L8K, NSP12_P323L, NSP12_V14R, Spike_D614G, N_D3L, Spike_S982A, N_S235F
CDR48	EPI_ISL_1970364	F	B.1.1.7	O	2021-04-22	Kurunegala	Male	28	NS8_Q27stop, NSP3_T183I, Spike_T716I, NS8_K68stop, NSP3_W1196C, NSP6_S106del, N_R203K, NSP3_D110Y, Spike_A570D, NSP15_E260A, Spike_N501Y, NSP3_I1412T, NS8_R52I, NSP8_S76F, Spike_P681H, Spike_Y144del, NSP6_G107del, NSP3_A890D, Spike_D1118H, NSP6_F108del, NS8_Y73C, N_G204R, NSP12_P323L, Spike_D614G, N_D3L, Spike_S982A
CDR49	EPI_ISL_1970365	F	B.1.1.7	O	2021-04-22	Kurunegala	Male	11	Spike_R21I, NS8_Q27stop, Spike_T716I, NS8_K68stop, NSP3_W1196C, NSP6_S106del, N_R203K, NSP3_D110Y, Spike_A570D, NSP15_E260A, Spike_N501Y, NSP3_I1412T, NS8_R52I, NSP8_S76F, Spike_P681H, Spike_Y144del, Spike_Q14H, NS3_R134C, NSP6_G107del, NSP3_A890D, Spike_D1118H, NSP6_F108del, NS8_Y73C, N_G204R, NSP12_P323L, Spike_D614G, N_D3L, Spike_S982A

CDR50	EPI_ISL_1970366	F	B.1.1.7	GRY	2021-04-22	Kurunegala	Male	31	Spike_H69del, NS8_Q27stop, NSP3_T183I, NSP8_T145I, Spike_T716I, NS8_K68stop, NSP6_S106del, N_R203K, Spike_A570D, Spike_N501Y, NSP3_I1412T, NS8_R52I, Spike_P681H, Spike_Y144del, NSP15_G246C, NSP6_G107del, NSP3_A890D, Spike_D1118H, NSP6_F108del, NS8_Y73C, N_G204R, Spike_V70del, NS3_E194D, NSP12_P323L, Spike_D614G, N_D3L, Spike_S982A, N_S235F
CDR51	EPI_ISL_1972229	F	B.1.1.7	GR	2021-04-22	Kurunegala	Male	27	Spike_H69del, NS8_Q27stop, NSP3_T183I, Spike_T716I, NS8_K68stop, NSP6_S106del, N_R203K, NSP3_D110Y, Spike_A570D, NSP15_E260A, NSP2_K110R, Spike_ins370LVPFWstopSF, Spike_N501Y, NSP3_R1297del, NSP3_I1412T, NS8_R52I, NSP8_S76F, Spike_P681H, NSP3_V1298del, NSP6_G107del, NSP3_A890D, Spike_D1118H, NSP6_F108del, N_G204R, Spike_V70del, NSP3_S1296del, NSP12_P323L, Spike_D614G, Spike_S982A, Spike_N370K, N_S235F
CDR53	EPI_ISL_1970368	F	B.1.1.7	GR	2021-04-22	Kurunegala	n/a	n/a	NSP3_T183I, Spike_T716I, NS8_K68stop, NSP6_S106del, N_R203K, NSP4_A231V, NS3_T89I, NSP15_E260A, Spike_N501Y, NSP3_I1412T, Spike_P681H, Spike_Y144del, NSP12_P227L, NSP6_G107del, NSP3_A890D, NSP3_D782N, Spike_D1118H, NSP6_F108del, NS8_Y73C, N_G204R, NSP12_P323L, Spike_D614G, N_D3L, Spike_S982A
CDR54	EPI_ISL_1972234	F	B.1.1.7	O	2021-04-22	Kurunegala	Female	n/a	Spike_H69del, NS8_Q27stop, NSP3_T183I, Spike_T716I, NS8_K68stop, NSP6_S106del, Spike_A570D, NSP15_E260A, NSP3_I1412T, NS8_R52I, NSP8_S76F, Spike_P681H, Spike_Y144del, NS7a_T14I, NSP6_G107del, NSP3_A890D, Spike_D1118H, NSP6_F108del, NS8_Y73C, NSP3_T779I, Spike_V70del, NSP12_P323L, Spike_D614G, N_D3L, Spike_S982A
CDR55	EPI_ISL_1970369	F	B.1.1.7	GRY	2021-04-22	Kurunegala	Female	n/a	Spike_H69del, NS8_Q27stop, NSP3_T183I, Spike_T716I, NS8_K68stop, NSP6_S106del, N_R203K, NSP3_D110Y, Spike_A570D, NSP15_E260A, Spike_N501Y, NSP3_I1412T, NS8_R52I, NSP8_S76F, Spike_P681H, Spike_Y144del, NS7a_T28I, NSP6_G107del, NSP3_A890D, Spike_D1118H, NSP6_F108del, NS8_Y73C, N_G204R, Spike_V70del, NSP12_P323L, Spike_D614G, N_D3L, Spike_S982A
CDR60	EPI_ISL_1970374	F	B.1.1.7	GRY	2021-04-22	Kurunegala	Female	52	Spike_H69del, NS8_Q27stop, NSP3_T183I, Spike_T716I, NS8_K68stop, NSP6_S106del, N_R203K, Spike_A570D, NSP15_E260A, Spike_N501Y, NSP3_I1412T, NS8_R52I, NSP8_S76F, Spike_P681H, Spike_Y144del, NSP6_G107del, NSP3_A890D, Spike_D1118H, NSP6_F108del, NS8_Y73C, NSP12_A699S, N_G204R, Spike_V70del, NSP12_P323L, Spike_D614G, N_D3L, Spike_S982A

CDR61	EPI_ISL_1970375	F	B.1.1.7	GRY	2021-04-22	Kurunegala	n/a	n/a	Spike_H69del, NS8_Q27stop, NSP3_T183I, NSP8_T145I, Spike_T716I, NS8_K68stop, NSP6_S106del, N_R203K, Spike_A570D, Spike_N501Y, NS6_W27L, NS8_R52I, Spike_P681H, Spike_Y144del, NSP6_G107del, NSP3_A890D, N_P46S, Spike_D1118H, NSP6_F108del, NS8_Y73C, N_G204R, Spike_V70del, NS3_E194D, Spike_D614G, N_D3L, Spike_S982A
CDR62	EPI_ISL_1970376	F	B.1.1.7	O	2021-04-22	Kurunegala	n/a	n/a	Spike_H69del, NS8_Q27stop, NSP3_T183I, Spike_T716I, NS8_K68stop, NSP6_S106del, N_R203K, NSP3_D110Y, Spike_A570D, NSP15_E260A, NSP2_K110R, Spike_N501Y, NSP3_I1412T, NS8_R52I, NSP8_S76F, Spike_P681H, Spike_Y144del, NSP6_G107del, NSP3_A890D, Spike_D1118H, NSP6_F108del, NS8_Y73C, N_G204R, Spike_V70del, NSP12_P323L, Spike_D614G, N_D3L, Spike_S982A, N_S235F
CDR63	EPI_ISL_1970377	F	B.1.1.7	GRY	2021-04-22	Kurunegala	Male	n/a	Spike_H69del, NS8_Q27stop, NSP3_T183I, Spike_T716I, NS8_K68stop, NSP6_S106del, N_R203K, NSP15_E260A, NSP2_K110R, Spike_N501Y, NSP3_I1412T, NS8_R52I, NSP8_S76F, Spike_P681H, Spike_Y144del, NSP6_G107del, NSP3_A890D, Spike_D1118H, NSP6_F108del, NS8_Y73C, N_G204R, Spike_V70del, NSP12_P323L, Spike_D614G, N_D3L, Spike_S982A
CDR79	EPI_ISL_1970392	F	B.1.1.7	GRY	2021-04-22	Polonnaruwa	Male	34	Spike_H69del, NS8_Q27stop, NSP3_T183I, Spike_T716I, NS8_K68stop, NSP6_S106del, N_R203K, NSP4_A231V, Spike_A570D, NS3_T89I, Spike_N501Y, NSP3_I1412T, NS8_R52I, Spike_Y144del, NSP12_P227L, Spike_L141F, NSP3_P67L, NSP6_G107del, NSP3_A890D, Spike_D1118H, NSP6_F108del, NS8_Y73C, N_G204R, NSP3_T275A, Spike_V70del, NSP12_P323L, NSP14_P451S, Spike_D614G, N_D3L, Spike_S982A, N_S235F
CDR52	EPI_ISL_1970367	F	B.1.411	O	2021-04-22	Kurunegala	Male	22	NSP12_D445G, N_D144N, NSP12_M666I, NSP6_L125F, Spike_H1159Y, N_T205I, NSP2_T166I, NSP2_T85I, NSP12_P323L, Spike_D614G, NSP6_L37F
CDR68	EPI_ISL_1970381	F	B.1.1.7	G	2021-04-23	Colombo	Female	82	Spike_H69del, NS8_Q27stop, NSP3_T183I, NSP8_T145I, Spike_T716I, NSP6_S106del, Spike_A570D, NSP14_A320V, Spike_N501Y, NSP3_I1412T, NS8_R52I, Spike_P681H, Spike_Y144del, NSP6_G107del, NSP3_A890D, Spike_D1118H, NSP6_F108del, NS8_Y73C, Spike_V70del, NS3_E194D, NSP12_P323L, Spike_D614G, N_D3L, Spike_S982A

CDR67	EPI_ISL_1970380	F	B.1.1.7	O	2021-04-27	Colombo	Female	91	Spike_H69del, NS8_Q27stop, NSP3_T183I, NSP8_T145I, Spike_T716I, NS8_K68stop, NSP6_S106del, N_R203K, Spike_A570D, Spike_N501Y, NSP3_I1412T, NS8_R52I, Spike_P681H, NS7a_R78C, Spike_Y144del, NSP6_G107del, NSP3_A890D, Spike_D1118H, NSP6_F108del, NS8_Y73C, N_G204R, Spike_V70del, NS3_E194D, NSP12_P323L, Spike_D614G, N_D3L, Spike_S982A
CDR74	EPI_ISL_1970387	F	B.1.1.7	GRY	2021-04-27	Polonnaruwa	Female	76	Spike_H69del, NS8_Q27stop, NSP3_T183I, NSP8_T145I, Spike_T716I, NS8_K68stop, NSP6_S106del, N_R203K, Spike_A570D, Spike_N501Y, NSP3_I1412T, NS8_R52I, Spike_P681H, NS7a_R78C, Spike_Y144del, NSP6_G107del, NSP3_A890D, Spike_D1118H, NSP6_F108del, NS8_Y73C, N_G204R, Spike_V70del, NS3_E194D, NSP12_P323L, Spike_D614G, N_D3L, Spike_S982A, N_S235F
CDR75	EPI_ISL_1970388	F	B.1.1.7	GRY	2021-04-27	Polonnaruwa	Female	52	Spike_H69del, NS8_Q27stop, Spike_T716I, NS8_K68stop, NSP6_S106del, N_R203K, Spike_A570D, Spike_N501Y, NSP3_I1412T, NS8_R52I, Spike_P681H, NS7a_R78C, Spike_Y144del, NSP6_G107del, Spike_D1118H, NSP6_F108del, NS8_Y73C, N_G204R, Spike_V70del, NS3_E194D, NSP12_P323L, Spike_D614G, Spike_S982A
CDR76	EPI_ISL_1970389	F	B.1.1.7	O	2021-04-27	Kalutara	Male	30	Spike_H69del, NS8_Q27stop, NSP3_T183I, NSP8_T145I, Spike_T716I, NS8_K68stop, NSP6_S106del, N_R203K, Spike_N501Y, NSP3_I1412T, NS8_R52I, Spike_P681H, Spike_Y144del, NSP6_G107del, NSP3_A890D, Spike_D1118H, NSP6_F108del, NS8_Y73C, N_G204R, Spike_V70del, NS3_E194D, NSP12_P323L, Spike_D614G, Spike_S982A, N_S235F
CDR77	EPI_ISL_1970390	F	B.1.1.7	GR	2021-04-27	Polonnaruwa	Male	51	NSP6_G107del, Spike_T716I, NSP6_S106del, N_R203K, Spike_A570D, Spike_D1118H, NSP6_F108del, N_G204R, NS3_E194D, NSP3_I1412T, Spike_P681H, Spike_D614G, N_D3L, Spike_S982A, N_S235F
CDR78	EPI_ISL_1970391	F	B.1.1.7	GRY	2021-04-27	Polonnaruwa	Male	34	Spike_H69del, NS8_Q27stop, NSP3_T183I, NSP8_T145I, NS8_K68stop, NSP6_S106del, N_R203K, Spike_A570D, Spike_N501Y, NSP3_I1412T, NS8_R52I, Spike_P681H, NS7a_R78C, Spike_Y144del, NSP6_G107del, NSP3_A890D, NSP6_F108del, NS8_Y73C, N_G204R, Spike_V70del, NS3_E194D, NSP12_P323L, Spike_D614G, N_D3L, Spike_S982A

CDR90	EPI_ISL_1970403	F	B.1.1.7	GRY	2021-04-27	Mannar	Male	37	Spike_H69del, NS8_Q27stop, NSP3_T183I, NSP8_T145I, Spike_T716I, NS8_K68stop, NSP6_S106del, N_R203K, Spike_A570D, Spike_N501Y, NSP3_I1412T, NS8_R52I, Spike_P681H, Spike_Y144del, NSP6_G107del, NSP3_A890D, NS7a_P99S, Spike_D1118H, NSP6_F108del, NS8_Y73C, N_G204R, Spike_V70del, NS3_E194D, NSP12_P323L, Spike_D614G, N_D3L, Spike_S982A, N_S235F
CDR91	EPI_ISL_1970404	F	B.1.1.7	G	2021-04-27	Kurunegala	Female	17	Spike_H69del, NS8_Q27stop, NSP3_T183I, Spike_T716I, NSP6_S106del, NSP3_D110Y, NSP15_E260A, Spike_N501Y, NSP3_I1412T, NS8_R52I, NSP8_S76F, Spike_P681H, Spike_Y144del, NSP6_G107del, NSP3_A890D, Spike_D1118H, NSP6_F108del, NS8_Y73C, Spike_V70del, NSP12_P323L, Spike_D614G, N_D3L, Spike_S982A, N_S235F
CDR92	EPI_ISL_1970405	F	B.1.1.7	GR	2021-04-27	Kurunegala	Male	49	NS8_Q27stop, NSP3_V1298del, NSP3_A890D, NSP6_G107del, Spike_T716I, NS8_K68stop, NSP6_S106del, N_R203K, Spike_A570D, Spike_D1118H, NSP6_F108del, NSP15_E260A, N_G204R, Spike_M153del, NSP3_S1296del, NSP3_R1297del, NS8_R52I, NSP8_S76F, Spike_P681H, Spike_D614G, N_D3L, Spike_S982A
CDR93	EPI_ISL_1970406	F	B.1.1.7	O	2021-04-27	Kurunegala	Female	52	Spike_H69del, NS8_Q27stop, Spike_T716I, NS8_K68stop, NSP3_W1196C, NSP6_S106del, N_R203K, NSP3_D110Y, Spike_A570D, NSP15_E260A, Spike_N501Y, NSP3_I1412T, NS8_R52I, NSP8_S76F, Spike_P681H, Spike_Y144del, NSP6_G107del, NSP3_A890D, Spike_D1118H, NSP6_F108del, NS8_Y73C, N_G204R, Spike_V70del, NSP12_P323L, Spike_D614G, N_D3L, Spike_S982A, N_S235F
CDR94	EPI_ISL_1970407	F	B.1.1.7	GRY	2021-04-27	Kurunegala	Female	17	Spike_H69del, NS8_Q27stop, NSP3_T183I, Spike_T716I, NS8_K68stop, NSP3_W1196C, NSP6_S106del, N_R203K, NSP3_D110Y, Spike_A570D, NSP15_E260A, Spike_N501Y, NSP3_R1297del, NSP3_I1412T, NS8_R52I, NSP8_S76F, Spike_P681H, Spike_Y144del, NSP3_V1298del, NSP6_G107del, NSP3_A890D, Spike_D1118H, NSP6_F108del, NS8_Y73C, N_G204R, Spike_V70del, NSP3_S1296del, NSP12_P323L, Spike_D614G, N_D3L, Spike_S982A, N_S235F
CDR95	EPI_ISL_1970408	F	B.1.1.7	GR	2021-04-27	Kurunegala	Male	31	Spike_H69del, NS8_Q27stop, NSP3_T183I, Spike_T716I, NS8_K68stop, NSP3_W1196C, NSP6_S106del, N_R203K, NSP3_D110Y, Spike_A570D, NSP15_E260A, Spike_N501Y, NSP3_I1412T, NS8_R52I, NSP8_S76F, Spike_P681H, NSP6_G107del, NSP3_A890D, Spike_D1118H, NSP6_F108del, NS8_Y73C, N_G204R, Spike_V70del, Spike_D614G, N_D3L, Spike_S982A

CDR96	EPI_ISL_1970409	F	B.1.1.7	GRY	2021-04-27	Kurunegala	Male	72	Spike_H69del, NS8_Q27stop, NSP3_T183I, Spike_T716I, NS8_K68stop, NSP6_S106del, N_R203K, Spike_A570D, NSP15_E260A, NSP2_K110R, NSP3_I1412T, NS8_R52I, NSP8_S76F, Spike_P681H, Spike_Y144del, NSP6_G107del, NSP3_A890D, Spike_D1118H, NSP6_F108del, NS8_Y73C, N_G204R, Spike_V70del, NSP9_T21I, NSP12_P323L, Spike_D614G, N_D3L, Spike_S982A, N_S235F
CDR97	EPI_ISL_1972228	F	B.1.1.7	G	2021-04-27	Kurunegala	Male	20	Spike_H69del, NS8_Q27stop, NSP15_R138L, NSP3_T183I, NS8_K68stop, NSP6_S106del, NSP3_D110Y, NSP15_E260A, NSP5_L67F, NSP3_R1297del, NSP3_I1412T, NS8_R52I, NSP8_S76F, Spike_P681H, Spike_Y144del, NSP3_V1298del, NSP3_A890D, NSP6_G107del, Spike_D1118H, NSP6_F108del, NS8_Y73C, Spike_V70del, NSP3_S1296del, NSP12_P323L, Spike_D614G, N_D3L, Spike_S982A, NSP3_S699F
CMC91808	EPI_ISL_1970414	F	B.1.1.7	G	2021-04-27	Colombo	Male	40	Spike_H69del, NS8_Q27stop, NSP3_T183I, NSP8_T145I, Spike_T716I, E_L21F, NS8_K68stop, NSP6_S106del, Spike_A570D, NSP14_A320V, Spike_N501Y, NSP3_I1412T, NS8_R52I, Spike_P681H, Spike_Y144del, NSP6_G107del, NSP3_A890D, Spike_D1118H, NSP6_F108del, NS8_Y73C, Spike_V70del, NS3_E194D, NSP12_P323L, Spike_D614G, N_D3L, Spike_S982A
CMC95123	EPI_ISL_1972226	F	B.1.1.7	O	2021-04-27	Nugegoda	Male	71	NS3_D250E, Spike_H69del, NS8_Q27stop, NSP6_S106del, N_R203K, Spike_A570D, NSP3_G277F, NSP3_A274K, NS8_R52I, NSP3_N276M, Spike_P681H, Spike_Y144del, E_K63T, NSP2_G235C, NSP3_T275P, NSP3_A890D, NSP6_G107del, NSP6_F108del, NSP3_D1208E, N_G204R, E_N64A, Spike_V70del, NSP3_I273L, NS3_E194D, NSP12_P323L, Spike_D614G, NSP3_K280N, NSP3_Y272H, N_D3L, NSP3_P278T, NSP3_V281M, N_S235F
CDR66	EPI_ISL_1970379	F	B.1.411	GH	2021-04-27	Colombo	Male	28	Spike_N679K, NSP12_D445G, NSP12_M666I, NS8_Q18stop, Spike_H1159Y, N_T205I, NS3_Q57H, NSP12_P323L, Spike_D614G, NSP6_L37F, Spike_V1040F
CDR70	EPI_ISL_1970383	F	B.1.411	O	2021-04-27	Batticaloa	Female	83	NS3_V255del, NS8_E59stop, NSP12_D445G, NSP3_P822L, NSP12_M666I, NS8_Q18stop, Spike_H1159Y, N_T205I, NS3_Q57H, NSP14_P203L, Spike_L5F, NSP2_T85I, NS3_W193L, NSP12_P323L, Spike_D614G, NSP6_L37F, Spike_G1167R, Spike_V1122L

CDR100	EPI_ISL_1970350	F	B.1.1.7	GRY	2021-04-28	Kurunegala	Female	17	Spike_H69del, NS8_Q27stop, NSP3_T183I, Spike_T716I, NS8_K68stop, NSP6_S106del, N_R203K, NSP4_A231V, Spike_A570D, NS3_T89I, NSP13_L581F, NSP3_I1412T, NS8_R52I, Spike_P681H, Spike_Y144del, NSP12_P227L, NSP3_P67L, NSP6_G107del, NSP3_A890D, NSP3_D782N, Spike_D1118H, NSP6_F108del, NS8_Y73C, N_G204R, NSP3_T275A, Spike_V70del, NSP5_A193V, NSP12_P323L, NSP14_P451S, Spike_D614G, N_D3L, Spike_S982A, N_S235F
CDR65	EPI_ISL_1970378	F	B.1.1.7	G	2021-04-28	Kalutara	Female	33	NSP8_E20K, NSP3_T183I, NSP8_T145I, NSP3_A890D, NSP6_G107del, Spike_T716I, NS8_K68stop, NSP6_S106del, Spike_D1118H, NSP6_F108del, NS8_Y73C, Spike_N501Y, NS3_E194D, NSP3_I1412T, NS8_R52I, NSP12_P323L, Spike_D614G, Spike_Y144del, N_D3L, Spike_S982A, N_S235F
CDR69	EPI_ISL_1970382	F	B.1.1.7	O	2021-04-28	Kalutara	Male	11	Spike_H69del, NSP3_T183I, NSP8_T145I, Spike_T716I, NS8_K68stop, NSP6_S106del, N_R203K, Spike_A570D, Spike_N501Y, NSP3_I1412T, Spike_P681H, Spike_Y144del, NSP6_G107del, NSP3_A890D, Spike_D1118H, NSP6_F108del, NS8_Y73C, N_G204R, Spike_V70del, NS3_E194D, NSP12_P323L, Spike_D614G, Spike_S982A
CDR73	EPI_ISL_1970386	F	B.1.1.7	O	2021-04-28	Panadura	Male	31	Spike_H69del, NS8_Q27stop, NSP3_T183I, Spike_T716I, NS8_K68stop, NSP6_S106del, N_R203K, Spike_A570D, NSP14_A320V, Spike_N501Y, NSP3_I1412T, NS8_R52I, Spike_P681H, Spike_Y144del, NSP6_G107del, NSP3_A890D, Spike_D1118H, NSP6_F108del, NS8_Y73C, N_G204R, Spike_V70del, NS3_E194D, NSP12_P323L, Spike_D614G, N_D3L, Spike_S982A
CDR80	EPI_ISL_1970393	F	B.1.1.7	G	2021-04-28	Panadura	Female	27	Spike_H69del, NS8_Q27stop, NSP3_T183I, NSP8_T145I, Spike_T716I, NS8_K68stop, NSP6_S106del, Spike_A570D, Spike_N501Y, NSP3_I1412T, NS8_R52I, Spike_P681H, Spike_Y144del, NSP6_G107del, NSP3_A890D, Spike_D1118H, NSP6_F108del, NS8_Y73C, Spike_V70del, NS3_E194D, NSP12_P323L, Spike_D614G, N_D3L
CDR81	EPI_ISL_1970394	F	B.1.1.7	G	2021-04-28	Panadura	Male	41	Spike_H69del, NS8_Q27stop, NSP3_T183I, NSP8_T145I, NSP3_A890D, NSP6_G107del, NS8_K68stop, NSP6_S106del, Spike_A570D, Spike_D1118H, NSP6_F108del, NS8_Y73C, Spike_V70del, NS3_E194D, NSP3_I1412T, NS8_R52I, NSP12_P323L, Spike_P681H, Spike_D614G, Spike_Y144del, N_D3L, Spike_S982A

CDR82	EPI_ISL_1970395	F	B.1.1.7	GR	2021-04-28	Panadura	Male	52	NS8_Q27stop, NSP3_T183I, NSP8_T145I, NS8_K68stop, NSP6_S106del, N_R203K, Spike_A570D, Spike_N501Y, NSP3_I1412T, NS8_R52I, Spike_Y144del, NSP6_G107del, NSP3_A890D, Spike_D1118H, NSP6_F108del, NS8_Y73C, N_G204R, NS3_E194D, NSP12_P323L, Spike_D614G, N_D3L, Spike_S982A, N_S235F
CDR83	EPI_ISL_1970396	F	B.1.1.7	O	2021-04-28	Kurunegala	Male	n/a	Spike_H69del, NS8_Q27stop, NSP3_T183I, Spike_T716I, NSP6_S106del, N_R203K, NSP3_D110Y, Spike_A570D, NSP15_E260A, NSP3_R1297del, NSP3_I1412T, NS8_R52I, NSP8_S76F, Spike_P681H, Spike_Y144del, NSP3_V1298del, NSP6_G107del, NSP3_A890D, Spike_D1118H, NSP6_F108del, N_G204R, Spike_V70del, NSP3_S1296del, NSP12_P323L, Spike_D614G, N_D3L, Spike_S982A, N_S235F
CDR84	EPI_ISL_1970397	F	B.1.1.7	GR	2021-04-28	Bandaragama	Male	18	Spike_H69del, NS8_Q27stop, NSP3_T183I, NS8_K68stop, NSP6_S106del, N_R203K, Spike_A570D, NSP3_I1412T, NS3_S180P, NS8_R52I, Spike_P681H, Spike_Y144del, NSP6_G107del, NSP3_A890D, Spike_D1118H, NSP6_F108del, N_G204R, Spike_V70del, NS3_E194D, NSP12_P323L, Spike_D614G, N_D3L, Spike_S982A
CDR85	EPI_ISL_1970398	F	B.1.1.7	G	2021-04-28	Bandaragama	Female	40	Spike_H69del, NS8_Q27stop, NSP3_T183I, NSP8_T145I, Spike_T716I, NS8_K68stop, NSP6_S106del, Spike_A570D, NSP14_A320V, Spike_N501Y, NSP3_I1412T, NS8_R52I, Spike_Y144del, NSP2_Q383H, NSP6_G107del, NSP3_A890D, N_D402V, Spike_D1118H, NSP6_F108del, NS8_Y73C, Spike_V70del, NS3_E194D, NSP12_P323L, Spike_D614G, N_D3L, Spike_S982A
CDR87	EPI_ISL_1970400	F	B.1.1.7	GR	2021-04-28	Mannar	Male	23	Spike_H69del, NS8_Q27stop, NSP3_T183I, NSP8_T145I, Spike_T716I, NS8_K68stop, NSP6_S106del, N_R203K, Spike_A570D, NSP3_I1412T, NS8_R52I, Spike_P681H, Spike_Y144del, NSP6_G107del, NSP3_A890D, Spike_D1118H, NSP6_F108del, NS8_Y73C, N_G204R, Spike_V70del, NS3_E194D, NSP12_P323L, Spike_D614G, N_D3L, Spike_S982A, N_S235F
CDR88	EPI_ISL_1970401	F	B.1.1.7	GR	2021-04-28	Mannar	Male	31	Spike_H69del, Spike_V1264L, NSP3_T183I, NSP8_T145I, Spike_T716I, NS8_K68stop, NSP6_S106del, N_R203K, Spike_A570D, NSP3_I1412T, NS8_R52I, Spike_P681H, Spike_Y144del, NSP6_G107del, NSP3_A890D, Spike_D1118H, NSP6_F108del, NS8_Y73C, N_G204R, Spike_V70del, NS3_E194D, NSP12_P323L, Spike_D614G, N_D3L, Spike_S982A, N_S235F

CDR89	EPI_ISL_1970402	F	B.1.1.7	GRY	2021-04-28	Kalutara	Female	58	Spike_H69del, NS8_Q27stop, NSP3_T183I, NSP8_T145I, Spike_T716I, NSP6_S106del, N_R203K, Spike_N501Y, NSP3_I1412T, NS8_R52I, Spike_P681H, Spike_Y144del, NSP6_G107del, NSP6_F108del, NS8_Y73C, N_G204R, Spike_V70del, NS3_E194D, NSP12_P323L, Spike_D614G, N_D3L, Spike_S982A, N_S235F
CDR98	EPI_ISL_1970410	F	B.1.1.7	G	2021-04-28	Kurunegala	Male	52	Spike_H69del, NS8_Q27stop, NSP3_T183I, NSP8_T145I, NSP6_L148F, Spike_T716I, NS8_K68stop, NSP6_S106del, Spike_A570D, Spike_N501Y, NSP3_I1412T, Spike_P681H, Spike_Y144del, NSP6_G107del, NSP3_A890D, Spike_D1118H, NSP6_F108del, NS8_Y73C, Spike_V70del, NS3_E194D, NSP12_P323L, Spike_D614G, N_D3L, Spike_S982A
CDR99	EPI_ISL_1970411	F	B.1.1.7	GR	2021-04-28	Kurunegala	Male	74	Spike_H69del, NSP3_T183I, Spike_T716I, NSP6_S106del, N_R203K, NSP4_A231V, Spike_A570D, NS3_T89I, NSP13_L581F, NSP3_I1412T, Spike_P681H, Spike_Y144del, NSP12_P227L, NSP3_P67L, NSP6_G107del, NSP3_A890D, Spike_D1118H, NSP6_F108del, N_G204R, NSP3_T275A, Spike_V70del, NSP5_A193V, NSP12_P323L, NSP14_P451S, Spike_D614G, N_D3L, Spike_S982A, N_S235F
CDR86	EPI_ISL_1970399	F	B.1.525	G	2021-04-28	Bandaragama	Female	30	Spike_H69del, NSP12_P323F, M_I82T, Spike_A67V, N_A12G, NSP3_T1189I, NSP6_G107del, E_L21F, NSP2_K443Q, NSP6_S106del, Spike_E484K, N_T205I, NSP6_F108del, Spike_Q52R, Spike_V70del, NS3_S92L, Spike_D614G, Spike_Y144del, Spike_F888L, Spike_Q677H
CDR101	EPI_ISL_1970351	F	B.1.1.7	G	2021-04-29	Kurunegala	Female	49	Spike_H69del, NS8_Q27stop, NSP3_T183I, NSP8_T145I, Spike_T716I, NS8_K68stop, NSP6_S106del, Spike_A570D, NSP14_A320V, Spike_N501Y, NSP3_I1412T, NS8_R52I, Spike_P681H, Spike_Y144del, NSP6_G107del, NSP3_A890D, Spike_E748V, Spike_D1118H, NSP6_F108del, NS8_Y73C, Spike_V70del, NS3_E194D, NSP12_P323L, Spike_D614G, N_D3L, Spike_S982A
CDR102	EPI_ISL_1972231	F	B.1.1.7	GRY	2021-04-29	Kurunegala	Female	61	Spike_H69del, NS8_Q27stop, NSP3_T183I, E_Y57F, Spike_T716I, NSP3_W1196C, NSP6_S106del, N_R203K, NSP15_E260A, Spike_N501Y, NSP3_R1297del, NSP3_I1412T, NS8_R52I, NSP8_S76F, Spike_P681H, Spike_S151del, NSP3_V1298del, NSP6_G107del, NSP3_A890D, Spike_D1118H, NSP6_F108del, E_F56A, N_G204R, Spike_V70del, NSP3_S1296del, NSP12_P323L, Spike_D614G, N_D3L, Spike_S982A, N_S235F

CMC94262	EPI_ISL_1970415	F	B.1.1.7	GRY	2021-04-29	Colombo	Male	56	Spike_H69del, NS8_Q27stop, NSP3_T183I, NSP8_T145I, Spike_T716I, NS8_K68stop, NSP6_S106del, N_R203K, Spike_A570D, Spike_N501Y, NSP3_I1412T, NS8_R52I, Spike_P681H, Spike_Y144del, NSP6_G107del, NSP3_A890D, NSP5_P96L, NSP3_G145D, Spike_D1118H, NSP6_F108del, NS8_Y73C, N_G204R, Spike_V70del, NS3_E194D, NSP12_P323L, Spike_D614G, N_D3L, Spike_S982A
