# Supplementary material for: Genomic and Epidemiological Analysis of SARS-CoV-2 Viruses in Sri Lanka
Source: Front Microbiol. 2021 Sep 16;12:722838. doi: 10.3389/fmicb.2021.722838 (PMC8483294; doi:10.3389/fmicb.2021.722838)
Supplement: Supplementary file 3 [file Data_Sheet_3.PDF]

Supplementary Table 2. Amino acid mutation counts of 231 sequences belonging to B.1.411 lineage.

Protein	AA_Substitution	Count	Mutation_ID
Spike	D614G	228	Spike_D614G
NSP12	M666I	224	NSP12_M666I
Spike	H1159Y	215	Spike_H1159Y
NSP6	L37F	205	NSP6_L37F
NSP2	T166I	202	NSP2_T166I
NSP12	P323L	201	NSP12_P323L
N	T205I	198	N_T205I
NS8	Q18stop	164	NS8_Q18stop
NSP12	D445G	154	NSP12_D445G
NS3	Q57H	147	NS3_Q57H
NSP2	T85I	109	NSP2_T85I
NSP3	P654S	19	NSP3_P654S
Spike	A684V	18	Spike_A684V
NS3	T151S	16	NS3_T151S
Spike	T676A	13	Spike_T676A
NSP6	C221F	10	NSP6_C221F
NSP5	K90R	9	NSP5_K90R
Spike	M1229I	9	Spike_M1229I
NS3	R30H	8	NS3_R30H
NS3	L108F	8	NS3_L108F
NS8	R52I	8	NS8_R52I
Spike	S98P	8	Spike_S98P
N	T265I	7	N_T265I
NSP16	R86K	7	NSP16_R86K
Spike	S640F	7	Spike_S640F
NS8	F120V	6	NS8_F120V
N	K373R	6	N_K373R
NS8	I121L	6	NS8_I121L
NSP6	E195D	6	NSP6_E195D
NSP12	V720I	6	NSP12_V720I
NSP9	M101I	6	NSP9_M101I
Spike	T716I	5	Spike_T716I
Spike	T33S	5	Spike_T33S
NSP15	T48I	5	NSP15_T48I
NSP14	S450I	5	NSP14_S450I
NSP7	T81I	5	NSP7_T81I
NSP12	T85I	5	NSP12_T85I
Spike	V70del	5	Spike_V70del
Spike	H69del	5	Spike_H69del
NSP13	H290Y	5	NSP13_H290Y
NSP3	S1670F	4	NSP3_S1670F
Spike	N679K	4	Spike_N679K
NSP13	L581F	4	NSP13_L581F
NS8	W45C	4	NS8_W45C
NSP14	P203L	4	NSP14_P203L
NS8	P93L	4	NS8_P93L
Spike	D287N	4	Spike_D287N
NSP2	K337E	4	NSP2_K337E

NSP3	K1804N	4	NSP3_K1804N
NSP16	Q3L	4	NSP16_Q3L
Spike	D178G	4	Spike_D178G
NSP2	G392R	4	NSP2_G392R
Spike	G1124V	4	Spike_G1124V
NSP14	S134F	4	NSP14_S134F
NSP9	T24I	4	NSP9_T24I
Spike	N439K	4	Spike_N439K
NSP13	A505P	4	NSP13_A505P
NSP13	A598S	4	NSP13_A598S
NSP3	I1683T	4	NSP3_I1683T
NSP3	K429N	3	NSP3_K429N
NSP14	K349N	3	NSP14_K349N
NSP12	Q292H	3	NSP12_Q292H
Spike	L5F	3	Spike_L5F
NSP3	A667T	3	NSP3_A667T
NSP3	A690V	3	NSP3_A690V
NSP2	H208Y	3	NSP2_H208Y
NSP3	H290Y	3	NSP3_H290Y
N	D144Y	3	N_D144Y
NS3	S171L	3	NS3_S171L
NSP13	S80G	3	NSP13_S80G
NSP2	T388I	3	NSP2_T388I
NSP8	T148I	3	NSP8_T148I
NS3	V13L	3	NS3_V13L
NSP3	Q1884H	3	NSP3_Q1884H
NS3	L106R	3	NS3_L106R
NSP3	A488V	3	NSP3_A488V
Spike	A222V	3	Spike_A222V
N	A134V	3	N_A134V
NSP3	A1311V	3	NSP3_A1311V
NSP3	S1265del	2	NSP3_S1265del
NSP3	T204I	2	NSP3_T204I
NSP3	T1072I	2	NSP3_T1072I
NSP2	E201A	2	NSP2_E201A
NS3	E102Q	2	NS3_E102Q
NSP6	V149F	2	NSP6_V149F
NS3	V255del	2	NS3_V255del
NS3	V202L	2	NS3_V202L
NSP3	L1328F	2	NSP3_L1328F
NSP3	L1266I	2	NSP3_L1266I
NSP15	A94T	2	NSP15_A94T
NS3	W193L	2	NS3_W193L
NSP4	I383F	2	NSP4_I383F
NSP1	G30S	2	NSP1_G30S
NSP2	G147D	2	NSP2_G147D
NS3	T175I	2	NS3_T175I
N	T362I	2	N_T362I
NSP2	T497K	2	NSP2_T497K
Spike	T76I	2	Spike_T76I
NSP3	K529R	2	NSP3_K529R

NSP12	E254D	2	NSP12_E254D
NS8	V62L	2	NS8_V62L
NSP9	V76A	2	NSP9_V76A
NSP7	V58A	2	NSP7_V58A
NS3	L41F	2	NS3_L41F
NS3	W131C	2	NS3_W131C
NSP13	D260Y	2	NSP13_D260Y
N	D402Y	2	N_D402Y
NS8	I58V	2	NS8_I58V
NSP3	G17C	2	NSP3_G17C
NSP3	M1556L	2	NSP3_M1556L
NSP3	L1096I	2	NSP3_L1096I
NSP2	L71F	2	NSP2_L71F
NS7a	I3F	2	NS7a_I3F
NSP3	S1675I	1	NSP3_S1675I
NSP14	S503L	1	NSP14_S503L
NSP3	S284C	1	NSP3_S284C
E	S55F	1	E_S55F
M	S4F	1	M_S4F
NSP10	T12I	1	NSP10_T12I
NSP3	T423I	1	NSP3_T423I
N	K387R	1	N_K387R
NSP3	K945N	1	NSP3_K945N
NS8	E59stop	1	NS8_E59stop
Spike	E484K	1	Spike_E484K
NSP1	E36K	1	NSP1_E36K
NSP12	Y546C	1	NSP12_Y546C
Spike	V213L	1	Spike_V213L
NS3	V50I	1	NS3_V50I
NSP5	V261F	1	NSP5_V261F
Spike	V1122L	1	Spike_V1122L
Spike	V367F	1	Spike_V367F
NS3	V55I	1	NS3_V55I
Spike	V1040F	1	Spike_V1040F
NSP15	V320L	1	NSP15_V320L
NSP16	Q238H	1	NSP16_Q238H
Spike	M1237I	1	Spike_M1237I
NS8	C83S	1	NS8_C83S
E	C40L	1	E_C40L
NS7a	L102P	1	NS7a_L102P
NSP6	L125F	1	NSP6_L125F
NS3	L53F	1	NS3_L53F
NS8	L95F	1	NS8_L95F
E	L39C	1	E_L39C
NSP2	A159S	1	NSP2_A159S
NS3	A59T	1	NS3_A59T
NSP3	A338H	1	NSP3_A338H
Spike	A879T	1	Spike_A879T
E	A41L	1	E_A41L
NSP12	W617F	1	NSP12_W617F
NSP3	P822L	1	NSP3_P822L

NS3	P262S	1	NS3_P262S
NSP14	P140S	1	NSP14_P140S
NSP3	P2L	1	NSP3_P2L
NSP3	ins336DHNY	1	NSP3_ins336DHNY
NSP12	ins617LR	1	NSP12_ins617LR
NSP14	H455Y	1	NSP14_H455Y
NS3	D210Y	1	NS3_D210Y
N	D144N	1	N_D144N
NSP12	D618N	1	NSP12_D618N
NSP12	D284N	1	NSP12_D284N
NSP3	D174Y	1	NSP3_D174Y
Spike	G261V	1	Spike_G261V
NSP3	G777V	1	NSP3_G777V
NSP3	G145C	1	NSP3_G145C
NSP1	G137S	1	NSP1_G137S
Spike	G1167R	1	Spike_G1167R
NSP3	G337S	1	NSP3_G337S
NSP14	G481S	1	NSP14_G481S
NSP9	G37R	1	NSP9_G37R
NSP15	S261L	1	NSP15_S261L
N	S201N	1	N_S201N
NSP2	S378F	1	NSP2_S378F
NSP15	S308Y	1	NSP15_S308Y
NS3	S92L	1	NS3_S92L
NSP6	F35L	1	NSP6_F35L
NSP6	F34V	1	NSP6_F34V
NSP14	F217del	1	NSP14_F217del
Spike	F1121L	1	Spike_F1121L
Spike	T1238S	1	Spike_T1238S
Spike	T859I	1	Spike_T859I
NSP2	T170I	1	NSP2_T170I
NS8	T26I	1	NS8_T26I
NSP3	T1303I	1	NSP3_T1303I
NSP6	T29P	1	NSP6_T29P
NSP14	T215K	1	NSP14_T215K
NSP6	T172A	1	NSP6_T172A
NSP3	N922S	1	NSP3_N922S
NSP12	N9Y	1	NSP12_N9Y
Spike	N751K	1	Spike_N751K
NS3	K67R	1	NS3_K67R
NSP2	K456R	1	NSP2_K456R
NS3	E102K	1	NS3_E102K
Spike	E180G	1	Spike_E180G
Spike	E154K	1	Spike_E154K
Spike	E748D	1	Spike_E748D
NS3	Y107C	1	NS3_Y107C
NSP14	Y361C	1	NSP14_Y361C
NSP2	V308L	1	NSP2_V308L
NSP10	V7L	1	NSP10_V7L
NSP3	V929I	1	NSP3_V929I
NSP13	V266L	1	NSP13_V266L

NSP3	V267F	1	NSP3_V267F
Spike	V622F	1	Spike_V622F
NSP1	V84del	1	NSP1_V84del
NS3	Q185H	1	NS3_Q185H
NSP6	Q30E	1	NSP6_Q30E
NSP12	Q822H	1	NSP12_Q822H
NSP3	Q995H	1	NSP3_Q995H
NSP1	M85V	1	NSP1_M85V
NSP14	C208del	1	NSP14_C208del
E	C40del	1	E_C40del
NSP10	C41S	1	NSP10_C41S
NSP14	C210del	1	NSP14_C210del
Spike	L585F	1	Spike_L585F
NSP3	L862F	1	NSP3_L862F
NSP14	L209del	1	NSP14_L209del
Spike	L18F	1	Spike_L18F
NSP3	L72F	1	NSP3_L72F
NSP12	L8C	1	NSP12_L8C
E	L39del	1	E_L39del
NSP6	L33M	1	NSP6_L33M
NS7b	L32F	1	NS7b_L32F
NSP3	A1321V	1	NSP3_A1321V
NSP3	A1105T	1	NSP3_A1105T
NSP3	A1280V	1	NSP3_A1280V
NSP8	A21V	1	NSP8_A21V
NS3	A23S	1	NS3_A23S
E	A41S	1	E_A41S
M	A2S	1	M_A2S
NSP6	W31Y	1	NSP6_W31Y
NSP16	P236L	1	NSP16_P236L
NSP3	P1228S	1	NSP3_P1228S
NSP3	P395L	1	NSP3_P395L
NSP13	P326L	1	NSP13_P326L
NSP12	ins9stop	1	NSP12_ins9stop
NSP6	ins35VF	1	NSP6_ins35VF
NSP6	ins171MTARTVYDDG	1	NSP6_ins171MTARTVYDDG
Spike	H1101Y	1	Spike_H1101Y
NSP3	H1880Y	1	NSP3_H1880Y
NSP1	H83del	1	NSP1_H83del
Spike	D936Y	1	Spike_D936Y
Spike	D253N	1	Spike_D253N
NSP14	D496Y	1	NSP14_D496Y
Spike	D80Y	1	Spike_D80Y
NSP14	D211del	1	NSP14_D211del
NSP16	D26H	1	NSP16_D26H
NSP12	D135Y	1	NSP12_D135Y
NS3	D199Y	1	NS3_D199Y
NSP12	R10C	1	NSP12_R10C
NSP14	R212H	1	NSP14_R212H
N	R209K	1	N_R209K
NSP14	R213H	1	NSP14_R213H

NSP3	I967T	1	NSP3_I967T
NSP13	I333V	1	NSP13_I333V
NSP1	G82del	1	NSP1_G82del
Spike	F186L	1	Spike_F186L
NSP13	F373Y	1	NSP13_F373Y
Spike	F92H	1	Spike_F92H
NSP3	S1206L	1	NSP3_S1206L
NSP14	S255I	1	NSP14_S255I
NSP2	S211H	1	NSP2_S211H
Spike	S94P	1	Spike_S94P
NSP3	S1443F	1	NSP3_S1443F
Spike	T63A	1	Spike_T63A
NS6	N39T	1	NS6_N39T
NSP3	N1680D	1	NSP3_N1680D
NSP16	N235R	1	NSP16_N235R
M	K15M	1	M_K15M
M	K14I	1	M_K14I
NS3	Y264H	1	NS3_Y264H
NSP3	Y519N	1	NSP3_Y519N
NSP2	E210D	1	NSP2_E210D
NS8	E106stop	1	NS8_E106stop
NSP3	V1936S	1	NSP3_V1936S
Spike	V1176F	1	Spike_V1176F
N	V72I	1	N_V72I
NSP10	V108del	1	NSP10_V108del
NSP3	V1935L	1	NSP3_V1935L
NSP2	V447F	1	NSP2_V447F
N	V270L	1	N_V270L
NSP13	V371F	1	NSP13_V371F
NSP13	V372A	1	NSP13_V372A
NSP5	Q306R	1	NSP5_Q306R
Spike	Q14H	1	Spike_Q14H
NS8	Q72H	1	NS8_Q72H
N	M210V	1	N_M210V
NSP2	M404V	1	NSP2_M404V
N	M234V	1	N_M234V
NSP12	M380L	1	NSP12_M380L
NSP4	C296F	1	NSP4_C296F
Spike	L841I	1	Spike_L841I
NSP2	L410F	1	NSP2_L410F
M	L16Q	1	M_L16Q
Spike	L1063F	1	Spike_L1063F
NSP4	L353F	1	NSP4_L353F
NSP2	L213S	1	NSP2_L213S
NSP2	L550I	1	NSP2_L550I
NS7a	A79V	1	NS7a_A79V
NSP7	A80V	1	NSP7_A80V
NS7b	A15S	1	NS7b_A15S
NSP14	A425V	1	NSP14_A425V
Spike	A783S	1	Spike_A783S
NSP4	A380V	1	NSP4_A380V

NSP4	A231V	1	NSP4_A231V
Spike	A93Y	1	Spike_A93Y
NSP3	P985L	1	NSP3_P985L
NSP3	P1261S	1	NSP3_P1261S
NSP13	P504S	1	NSP13_P504S
Spike	P1263L	1	Spike_P1263L
NSP10	P107del	1	NSP10_P107del
N	P199S	1	N_P199S
NS7a	P99S	1	NS7a_P99S
NSP16	ins234MstopM	1	NSP16_ins234MstopM
NSP13	D369E	1	NSP13_D369E
N	D128Y	1	N_D128Y
NSP3	I617V	1	NSP3_I617V
NSP2	R222C	1	NSP2_R222C
NS3	G174V	1	NS3_G174V
NSP2	G212T	1	NSP2_G212T
NS7a	G38stop	1	NS7a_G38stop
