# Supplementary material for: Genomic and Epidemiological Analysis of SARS-CoV-2 Viruses in Sri Lanka
Source: Front Microbiol. 2021 Sep 16;12:722838. doi: 10.3389/fmicb.2021.722838 (PMC8483294; doi:10.3389/fmicb.2021.722838)
Supplement: Supplementary file 4 [file Data_Sheet_4.PDF]

Supplementary Table 3. Amino acid mutation counts of 93 sequences belonging to B.1.1.7 lineage.

Protein	AA_Substitution	Count	Mutation_ID
Spike	D614G	93	Spike_D614G
NSP6	F108del	92	NSP6_F108del
NSP6	G107del	92	NSP6_G107del
NSP6	S106del	92	NSP6_S106del
Spike	D1118H	90	Spike_D1118H
N	D3L	88	N_D3L
NSP3	A890D	88	NSP3_A890D
Spike	S982A	88	Spike_S982A
NSP12	P323L	87	NSP12_P323L
NSP3	I1412T	87	NSP3_I1412T
NSP3	T183I	85	NSP3_T183I
Spike	P681H	85	Spike_P681H
NS8	Q27stop	84	NS8_Q27stop
NS8	R52I	84	NS8_R52I
Spike	Y144del	80	Spike_Y144del
NS8	Y73C	78	NS8_Y73C
Spike	T716I	76	Spike_T716I
N	G204R	75	N_G204R
N	R203K	75	N_R203K
Spike	A570D	75	Spike_A570D
Spike	H69del	75	Spike_H69del
Spike	V70del	75	Spike_V70del
Spike	N501Y	72	Spike_N501Y
NS8	K68stop	70	NS8_K68stop
N	S235F	49	N_S235F
NS3	E194D	34	NS3_E194D
NSP15	E260A	28	NSP15_E260A
NSP8	S76F	27	NSP8_S76F
NSP8	T145I	26	NSP8_T145I
NSP3	D110Y	19	NSP3_D110Y
NSP3	R1297del	12	NSP3_R1297del
NSP3	S1296del	12	NSP3_S1296del
NSP3	V1298del	12	NSP3_V1298del
NSP12	P227L	9	NSP12_P227L
NSP3	W1196C	9	NSP3_W1196C
NS3	T89I	8	NS3_T89I
NSP14	P451S	8	NSP14_P451S
NSP3	P67L	8	NSP3_P67L
NSP3	D782N	7	NSP3_D782N
NSP14	A320V	6	NSP14_A320V
NS3	T151I	5	NS3_T151I
NS7a	Q62stop	5	NS7a_Q62stop
NS7a	R78C	4	NS7a_R78C
NSP2	K110R	4	NSP2_K110R
NSP4	A231V	4	NSP4_A231V
Spike	L5F	4	Spike_L5F
N	R195I	3	N_R195I
NSP3	T275A	3	NSP3_T275A

NS3	E261G	2	NS3_E261G
NS3	W131C	2	NS3_W131C
NSP1	V106A	2	NSP1_V106A
NSP13	L581F	2	NSP13_L581F
NSP15	S288F	2	NSP15_S288F
NSP2	L400F	2	NSP2_L400F
NSP3	D339G	2	NSP3_D339G
NSP3	R407I	2	NSP3_R407I
NSP5	A193V	2	NSP5_A193V
NSP5	L75F	2	NSP5_L75F
Spike	N149del	2	Spike_N149del
E	F56A	1	E_F56A
E	ins38CLL	1	E_ins38CLL
E	ins38CLX	1	E_ins38CLX
E	K63T	1	E_K63T
E	L21F	1	E_L21F
E	N64A	1	E_N64A
E	Y57F	1	E_Y57F
N	D402V	1	N_D402V
N	P46S	1	N_P46S
NS3	D250E	1	NS3_D250E
NS3	G100C	1	NS3_G100C
NS3	L15F	1	NS3_L15F
NS3	Q57H	1	NS3_Q57H
NS3	R134C	1	NS3_R134C
NS3	S180P	1	NS3_S180P
NS6	W27L	1	NS6_W27L
NS7	P99S	1	NS7a_P99S
NS7a	T14I	1	NS7a_T14I
NS7a	T28I	1	NS7a_T28I
NS7b	S31L	1	NS7b_S31L
NS8	Q18stop	1	NS8_Q18stop
NSP12	A699S	1	NSP12_A699S
NSP12	F7L	1	NSP12_F7L
NSP12	G13T	1	NSP12_G13T
NSP12	ins772LstopA	1	NSP12_ins772LstopA
NSP12	ins774TLR	1	NSP12_ins774TLR
NSP12	L775M	1	NSP12_L775M
NSP12	L8K	1	NSP12_L8K
NSP12	N9I	1	NSP12_N9I
NSP12	Q5H	1	NSP12_Q5H
NSP12	Q773H	1	NSP12_Q773H
NSP12	R10A	1	NSP12_R10A
NSP12	S6stop	1	NSP12_S6stop
NSP12	V11M	1	NSP12_V11M
NSP12	V14R	1	NSP12_V14R
NSP13	K460R	1	NSP13_K460R
NSP13	S38L	1	NSP13_S38L
NSP15	G246C	1	NSP15_G246C
NSP15	H234Y	1	NSP15_H234Y
NSP15	R138L	1	NSP15_R138L

NSP15	T33I	1	NSP15_T33I
NSP2	G235C	1	NSP2_G235C
NSP2	Q383H	1	NSP2_Q383H
NSP2	S430L	1	NSP2_S430L
NSP3	A274K	1	NSP3_A274K
NSP3	D1208E	1	NSP3_D1208E
NSP3	G145D	1	NSP3_G145D
NSP3	G277F	1	NSP3_G277F
NSP3	I273L	1	NSP3_I273L
NSP3	I441L	1	NSP3_I441L
NSP3	K280N	1	NSP3_K280N
NSP3	L1700F	1	NSP3_L1700F
NSP3	L689F	1	NSP3_L689F
NSP3	M560I	1	NSP3_M560I
NSP3	N276M	1	NSP3_N276M
NSP3	P278T	1	NSP3_P278T
NSP3	P402L	1	NSP3_P402L
NSP3	S699F	1	NSP3_S699F
NSP3	T1501I	1	NSP3_T1501I
NSP3	T1830I	1	NSP3_T1830I
NSP3	T275P	1	NSP3_T275P
NSP3	T779I	1	NSP3_T779I
NSP3	V281M	1	NSP3_V281M
NSP3	Y272H	1	NSP3_Y272H
NSP4	F17L	1	NSP4_F17L
NSP5	L67F	1	NSP5_L67F
NSP5	P96L	1	NSP5_P96L
NSP6	L148F	1	NSP6_L148F
NSP6	L37F	1	NSP6_L37F
NSP8	E20K	1	NSP8_E20K
NSP9	T21I	1	NSP9_T21I
Spike	E748V	1	Spike_E748V
Spike	E96D	1	Spike_E96D
Spike	G1251V	1	Spike_G1251V
Spike	ins370KLVPFWstopSF	1	Spike_ins370KLVPFWstopSF
Spike	ins370LVPFWstopSF	1	Spike_ins370LVPFWstopSF
Spike	L141F	1	Spike_L141F
Spike	M153del	1	Spike_M153del
Spike	N370K	1	Spike_N370K
Spike	N370Y	1	Spike_N370Y
Spike	Q14H	1	Spike_Q14H
Spike	R21I	1	Spike_R21I
Spike	S151del	1	Spike_S151del
Spike	V1264L	1	Spike_V1264L
