# Supplementary material for: Genomic and Epidemiological Analysis of SARS-CoV-2 Viruses in Sri Lanka
Source: Front Microbiol. 2021 Sep 16;12:722838. doi: 10.3389/fmicb.2021.722838 (PMC8483294; doi:10.3389/fmicb.2021.722838)
Supplement: Supplementary file 6 [file Table_1.DOCX]

﻿

|  |
| --- |
| Figure S1. Time-scaled Bayesian phylogeny with posterior support values labelled. |

| 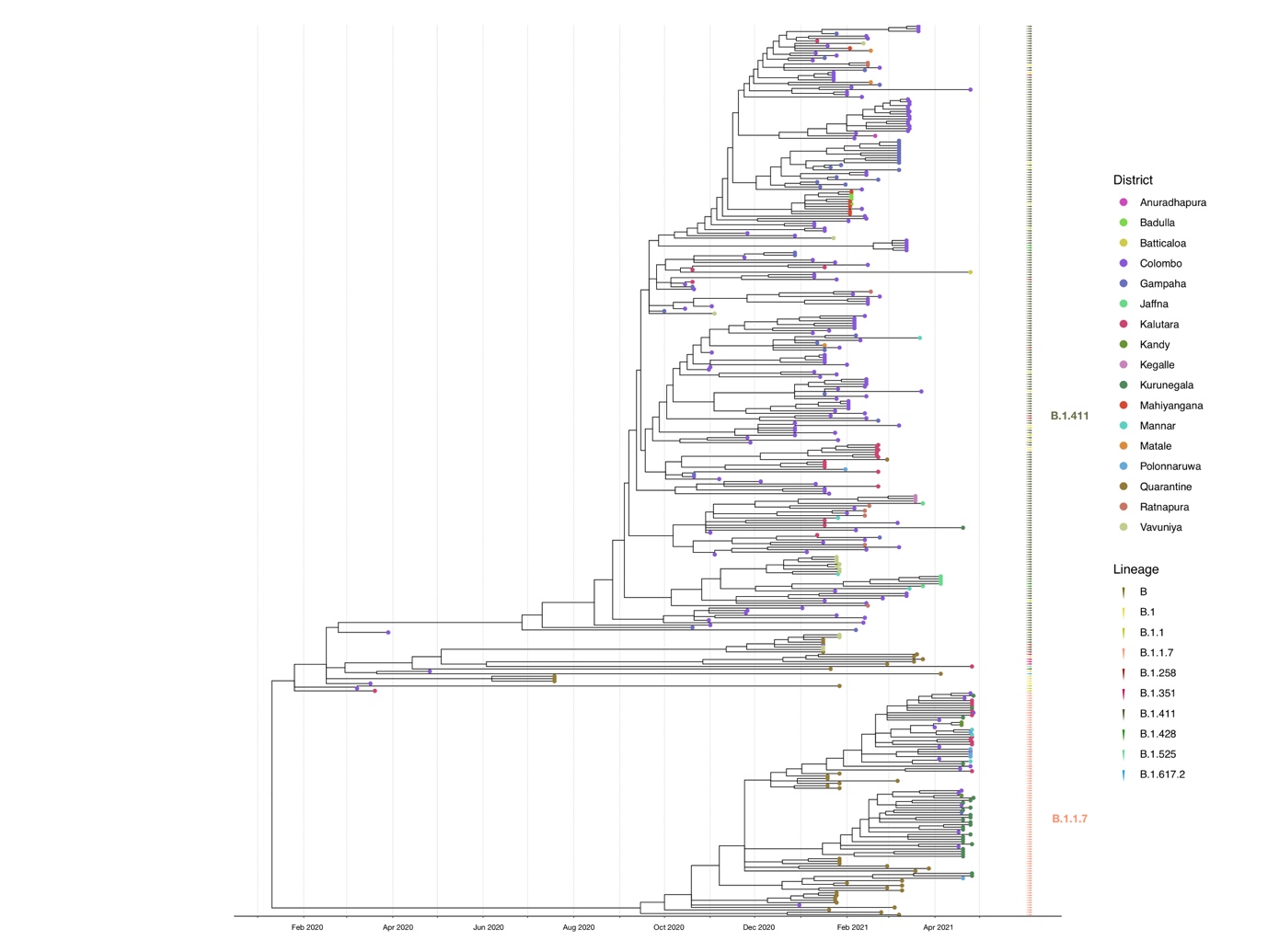 |
| --- |
| Figure S2. Time-scaled Maximum-likelihood of Sri Lanka samples.  Tips are coloured by the sample location, and the external layer on the right shows the sample lineages. The molecular clock was inferred using TreeTime with an evolutionary rate of 1.1 × 10−3 substitutions/site/year (estimated by Duchene et al.[8]) and a standard deviation of 0.00004. The tree was rerooted with least-squares criteria in TreeTime. 1000 replicates of ultrafast bootstrapping (-B 1000) and SH-aLRT branch test (-alrt 1000) were used for the analysis. |
