# Supplementary material for: Genomic and Epidemiological Analysis of SARS-CoV-2 Viruses in Sri Lanka
Source: Front Microbiol. 2021 Sep 16;12:722838. doi: 10.3389/fmicb.2021.722838 (PMC8483294; doi:10.3389/fmicb.2021.722838)
Supplement: Supplementary file 7 [file Table_2.DOCX]

**Supplementary methods**

TruSeq Stranded Total RNA Library Preparation

The starting RNA concentrations of the extracted samples were quantified using a NanoDrop™ 2000c Spectrophotometers (Thermo Fisher Scientific, USA). Ribosomal RNA was removed using biotinylated, target-specific oligos combined with Ribo-Zero rRNA removal beads. Following purification, the RNA was fragmented into small pieces using divalent cations under elevated temperature. All the thermal cycling steps were carried out in a CFX96 Deep Well, Real time System (Bio-Rad, USA). Following first and second strand cDNA synthesis 3’ ends of the blunt fragments were adenylated. Adapter ligation was carried out using IDT for Illumina- TruSeq RNA ud Indexes (Illumina, San Diego, USA). Ligated fragments were cleaned up using Agencourt AMPure XP beads (Beckman Coulter Genomics, USA) followed by DNA fragment enrichment using PCR. The libraries were quantified by using the Qubit dsDNA High Sensitivity assay kit on a Qubit 4.0 instrument (Life Technologies), and the fragment sizes (260bp) were analyzed by gel electrophoresis using a 1% gel. Each sample library was normalized to 10 nM concentration, and the normalized libraries were pooled and denatured with 0.2N NaOH. The denatured library pool was further diluted to 1.5pM and was sequenced on an Illumina NextSeq 550 platform.

The AmpliSeq for Illumina SARS-CoV-2 Community Panel

Input RNA was quantified using the Qubit RNA High Sensitivity assay kit on a Qubit 4.0 instrument (Life Technologies). RNA was reverse transcribed to cDNA using the AMpliSeq cDNA synthesis for Illumina Kit. All the thermal cycling steps were carried out in CFX96 Deep Well, Real time System (Bio-Rad, USA). Target regions of the cDNA were amplified using a panel with a 2-pool design, containing 247 amplicons/primer pairs (242 unique amplicons (Pool 1: 125 amplicon, Pool 2: 122 amplicons): 237 viral specific SARS-CoV-2 targets and 5 human gene expression controls) ranging from 125-275 bps in length that covers >99% of the Coronavirus genome (~30kb) and all potential serotypes of the virus. Ampliseq CD Indexes were ligated to cDNA using DNA ligase, followed by a cleanup using Agencourt AMPure XP beads (Beckman Coulter Genomics, USA). The prepared libraries were amplified and were cleaned up again to ensure sufficient quantity for sequencing on the Illumina system. The libraries were quantified by using the Qubit dsDNA High Sensitivity assay kit on a Qubit 4.0 instrument (Life Technologies), and the fragment sizes (350bp) were analyzed by gel electrophoresis using 1% gel. Each sample library was normalized to 2nM concentration and the normalized libraries were pooled, diluted to 100pM and sequenced on an Illumina iSeq 100 platform. The final 96 samples were normalized to 2nM concentration and the normalized libraries were pooled and denatured with 0.2N NaOH. The denatured library pool was further diluted to 1.8pM and was sequenced on an Illumina NextSeq 550 platform.

Sequence Data Analysis

The first 4 samples were sequenced on Illumina Nextseq 550 platform using shotgun metagenomics workflow. Resultant base calls files were demultiplexed and converted to FASTQ files via BaseSpace Sequence Hub^1^ Fastq generator. Base quality of the sequenced files were checked and confirmed using fastqc^2^ tool. To assess the quantity of host genomic DNA and other contaminants, Kraken 2 (version 2.0.1) Metagenomics pipeline^3^ was used on the BaseSpace Sequence Hub and the abundance data were visualized using Krona^4^ charts. After confirming the quantities of host DNA and viral DNA, FASTQ files were again piped through DRAGEN^5^ RNA Pathogen Detection (version 3.5.14) pipeline to map reads against SARS-CoV-2 Wuhan-Hu-1 isolate (Genbank accession number: MN908947.3). The DRAGEN pipeline generated coverage plots, binary alignment files (BAM)^6^ and variant call files (VCF)^7^ against the accession MN908947.3. Since all four metagenomic sequences had very high depth over the SARS-CoV2 genome, consensus sequences were directly generated from the hard-filtered VCF files using bcftools (version 1.10.2)^6^ consensus algorithm.

Amplicon based targeted sequencing of 150bp paired end library was performed on Illumina iSeq100 and Nextseq 550 platforms. Raw base calls of 236 samples were converted to FASTQ files using BaseSpace Sequencing Hub Fastq generator and inspected with fastqc for low quality reads. FASTQ files were aligned against SARS-CoV-2 Wuhan-Hu-1 isolate (Genbank accession number: MN908947.3) and BAM and VCF files were generated via DRAGEN RNA Pathogen Detection (version 3.5.14) pipeline. Resulting BAM files were checked using SAMTOOLS^6^ (version 1.10) flagstat algorithm for alignment quality and samples with more than 86% genome coverage and moderate to low coverage depth were proceeded to obtain consensus sequences using DRAGEN pipeline. The variants were visualized and further analyzed with the VCF files alongside the BAM alignments by Interactive Genomics Viewer^8^ (IGV) in order to confirm frameshift mutations.

**Reference**

1 BaseSpace Sequence Hub (2020).

2 Andrews, S. (2010). FastQC: A Quality Control Tool for High Throughput Sequence Data [Online]. Available online at: http://www.bioinformatics.babraham.ac.uk/projects/fastqc/

3 Wood, D. E. & Salzberg, S. L. Kraken: ultrafast metagenomic sequence classification using exact alignments. Genome Biol 15, R46, doi:10.1186/gb-2014-15-3-r46 (2014).

4 Ondov, B. D., Bergman, N. H. & Phillippy, A. M. Interactive metagenomic visualization in a Web browser. BMC Bioinformatics 12, 385, doi:10.1186/1471-2105-12-385 (2011).

5 DRAGEN-RNA-Pathogen-Detection (Illumina, 2019).

6 Li, H. et al. The Sequence Alignment/Map format and SAMtools. Bioinformatics 25, 2078-2079, doi:10.1093/bioinformatics/btp352 (2009).

7 Danecek, P. et al. The variant call format and VCFtools. Bioinformatics 27, 2156-2158, doi:10.1093/bioinformatics/btr330 (2011).

8 Robinson, J. T. et al. Integrative genomics viewer. Nature biotechnology 29, 24-26, doi:10.1038/nbt.1754 (2011).
